# Gold-catalyzed oxycyclization of allenic carbamates: expeditious synthesis of 1,3-oxazin-2-ones

**DOI:** 10.3762/bjoc.9.93

**Published:** 2013-04-26

**Authors:** Benito Alcaide, Pedro Almendros, M Teresa Quirós, Israel Fernández

**Affiliations:** 1 Grupo de Lactamas y Heterociclos Bioactivos, Departamento de Química Orgánica I, Unidad Asociada al CSIC, Facultad de Química, Universidad Complutense de Madrid, 28040-Madrid, Spain; 2 Instituto de Química Orgánica General (IQOG), Consejo Superior de Investigaciones Científicas (CSIC), Juan de la Cierva 3, 28006-Madrid, Spain; 3 Departamento de Química Orgánica I, Facultad de Química, Universidad Complutense de Madrid, 28040-Madrid, Spain

**Keywords:** allenes, computational chemistry, gold, gold catalysis, heterocycles, reaction mechanisms

## Abstract

A combined experimental and computational study on regioselective gold-catalyzed synthetic routes to 1,3-oxazinan-2-ones (kinetically controlled products) and 1,3-oxazin-2-one derivatives (thermodynamically favored) from easily accessible allenic carbamates has been carried out.

## Introduction

The search for new synthetic routes to 1,3-oxazin-2-one derivatives [1] is of interest because of the biological activity of these molecules [2–7]. Carretero and colleagues have published the Au(I)-catalyzed cyclization of *N*-Boc-3-butyn-1-amines to afford six-membered 2-oxazinones, namely, 6-methylene-1,3-oxazinan-2-ones involving a 6-*exo*-*dig* cyclization [1]. This interesting method avoids complicated prefunctionalization of starting materials and minimizes the formation of byproducts; however, it is used just for the preparation of four simple examples. 1,3-Oxazin-2-ones are also used as valuable intermediates in organic synthesis [8–14]. Recently, allenes have attracted much attention as they have been used for the preparation of both biologically relevant drugs as well as advanced materials [15–27]. However, regioselectivity problems are significant (*endo*-*trig* versus *endo*-*dig* versus *exo*-*dig* versus *exo*-*trig* cyclization). Great effort is currently being made in the search for a variety of reactions promoted by gold salts due to their impressive catalytic properties [28–41]. The Boc protective group has been widely used in allene chemistry, being an inert and recommended partner in gold- and palladium-catalyzed aminocyclizations of allenes [42]. On the other hand, reports of gold-catalyzed cyclizations leading to heterocycles that contain more than one heteroatom are rare [43–48]. Besides, it has been reported very recently that the Au(I)-catalyzed cyclization of a *N*-phenethyl-*N*-Boc-protected allenamide failed [49]. Despite the above precedents, but in continuation of our interest in heterocyclic and allene chemistry [50–55], we decided to examine the gold-catalyzed cyclization of *N*-Boc-allenes with the aim of establishing a protocol for the synthesis of 1,3-oxazin-2-one derivatives in which the carbamate group should serve as the source of CO_2_.

## Results and Discussion

**Scheme 1:**
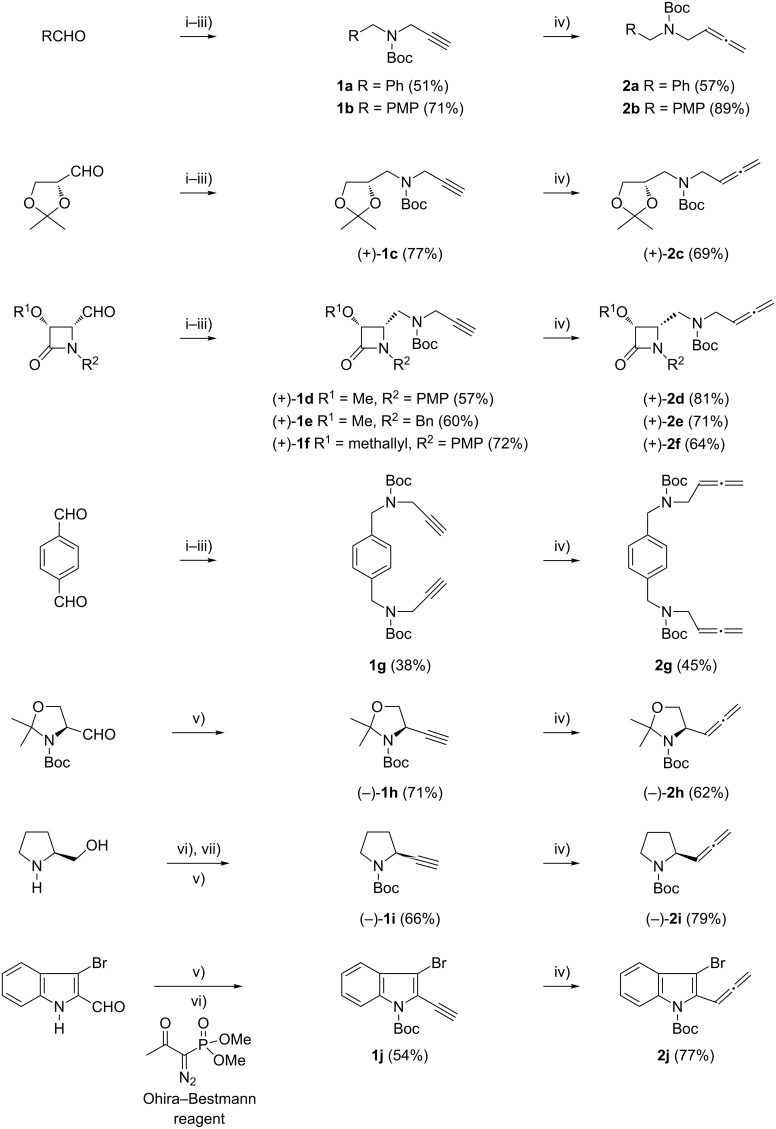
Preparation of allenic carbamates **2a**–**j**. Reagents and conditions: (i) Propargylamine, MgSO_4_, CH_2_Cl_2_, rt, 15 h. (ii) NaBH_4_, MeOH, rt, 0.5 h. (iii) Boc_2_O, Et_3_N, CH_2_Cl_2_, rt, 2–15 h. (iv) (CH_2_O)*_n_*, iPr_2_NH, CuBr, 1,4-dioxane, reflux, 1 h. (v) Ohira–Bestmann reagent, K_2_CO_3_, MeOH, rt, 15 h. (vi) Boc_2_O, DMAP, CH_3_CN, rt, 2 h. (vii) Dess–Martin periodinane, CH_2_Cl_2_, rt. PMP = 4-MeOC_6_H_4_.

To explore the effects of various substrates on gold-catalyzed oxycyclization reactions, a number of new allenic carbamates were synthesized as shown in Scheme 1. Starting materials, *tert*-butyl (prop-2-ynyl)carbamates **1a**–**j**, were obtained both in the racemic form and in optically pure form by using standard methodologies. Thus, alkynylcarbamates **1a**–**g** were prepared through reductive amination of the appropriate aldehyde with propargylamine, followed by Boc_2_O treatment of the corresponding N-substituted prop-2-yn-1-amine. Alkynylcarbamate **1h** was prepared from Garner’s aldehyde following a literature report [56–57]. Alkynylcarbamate **1i** was readily accessed from (*S*)-prolinol by using a modified known procedure [58]. Alkynylcarbamate **1j** was achieved through the reaction of 3-bromo-1*H*-indole-2-carbaldehyde with the Ohira–Bestmann reagent followed by the addition of Boc_2_O. Terminal alkynes **1** were conveniently converted into allenic carbamates **2** by treatment with paraformaldehyde in the presence of diisopropylamine and copper(I) bromide (Crabbé reaction) [59–60].

**Scheme 2:**
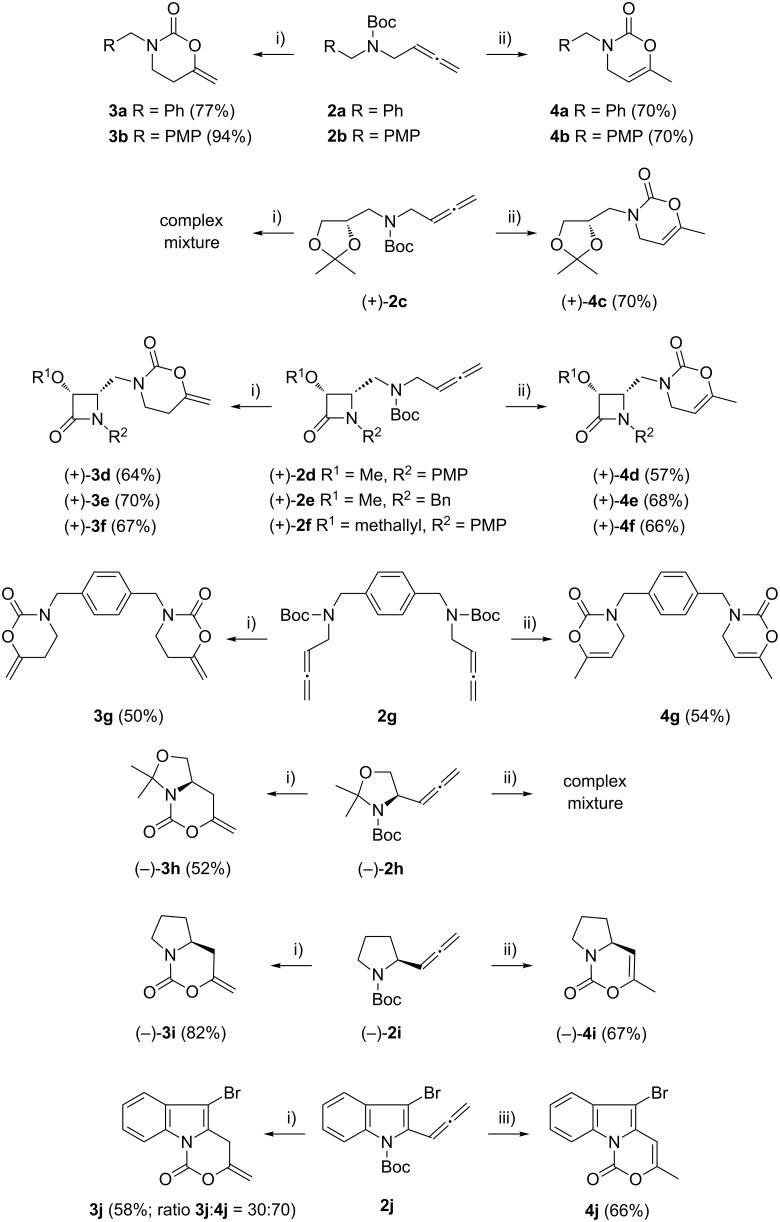
Controlled oxycyclization reactions of allenic carbamates **2** to 1,3-oxazinan-2-ones **3** and 1,3-oxazin-2-ones **4** under selective gold-catalyzed conditions. Reagents and conditions: (i) 2.5 mol % [AuClPPh_3_], 2.5 mol % AgOTf, 10 mol % PTSA, CH_2_Cl_2_, rt, **3a**: 6 h; **3b**: 7 h, **3d**: 6 h; **3e**: 8 h; **3f**: 7 h; **3g**: 5.5 h; **3h**: 5 h; **3i**: 7 h; **3j**: 2 h. (ii) 2.5 mol % [AuClPPh_3_], 2.5 mol % AgOTf, 10 mol % PTSA, CH_2_Cl_2_, sealed tube, 130 °C, **4a**: 1.5 h; **4b**: 2 h, **4c**: 4 h; **4d**: 6 h; **4e**: 4 h; **4f**: 0.5 h; **4g**: 5.5 h; **4i**: 2 h. (iii) 2.5 mol % [AuClPPh_3_], 2.5 mol % AgOTf, 10 mol % PTSA, CH_2_Cl_2_, sealed tube, 80 °C, **4j**: 2 h. PMP = 4-MeOC_6_H_4_.

We employed three different gold salts in our initial screening of catalysts for the model system, allenic carbamate **2a**. Initially, the use of AuCl_3_ and AuCl were tested, but both failed to catalyze the reaction. Fortunately, we found that [AuClPPh_3_]/AgOTf was an excellent catalyst for our purpose. To our delight, the reaction of allenic carbamate **2a** at room temperature afforded 3-benzyl-6-methylene-1,3-oxazinan-2-one (**3a**) bearing an exocyclic double bond as the sole product (Scheme 2). Adding a catalytic amount of Brønsted acid (PTSA) into the reaction system did slightly improve the yield of **3a**. Solvent screening demonstrated that dichloromethane was the best choice in the reaction.

As revealed in Scheme 2, a variety of allenic carbamates **2** were also suitable for such heterocyclization reactions to afford 1,3-oxazinan-2-ones **3**. To increase the molecular diversity by incorporating more 1,3-oxazin-2-ones in the molecule, compound **2g** having two allenic carbamate units was used. Notably, bis(allenic carbamate) **2g** also undergoes this interesting transformation to give bis(6-methylene-1,3-oxazinan-2-one) **3g** through a two-fold cyclization. This product particularly underlines the power of the present cyclization reaction, as none of the conventional methods would allow its synthesis with such great ease.

Interestingly, as a first try, we were pleased to notice that the reaction of allenyl derivative **2a** in dichloromethane at 90 °C, afforded 1,3-oxazin-2-one **4a** bearing an endocyclic double bond as the major component, and 1,3-oxazinan-2-one **3a** was also isolated as a minor component. Notably, starting from allenic carbamates **2a–j** and performing the reaction in dichloromethane at 130 °C, a series of 6-methyl-3-substituted 3,4-dihydro-2*H*-1,3-oxazin-2-ones **4a–j** were exclusively formed (Scheme 2) [61–68]. The observed regioselectivity is worthy of note, because under our reaction conditions only 1,3-oxazinan-2-ones **3** (arising from 6-*endo*-*dig* cyclization) or 3,4-dihydro-2*H*-1,3-oxazin-2-ones **4** (arising from 6-*exo*-*dig* cyclization) were achieved, with the nucleophilic oxygen attacking the central allene carbon atom in each case. This is an interesting result, because the available examples on related metal-catalyzed allene heterocyclizations usually lead to 5-*exo*-*trig* cyclization [69–70]; only Hashmi et al. have recently reported an attack at the central position of the allene in allenylamides [44].

**Scheme 3:**
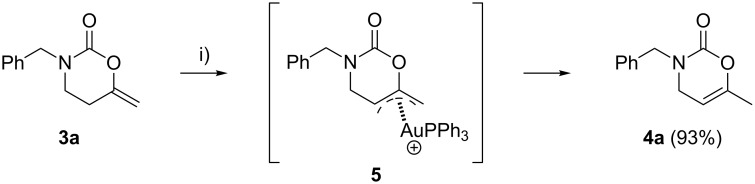
Possible explanation for the gold-catalyzed isomerization reaction of 1,3-oxazinan-2-one **3a** into 1,3-oxazin-2-one **4a**. Reagents and conditions: (i) 2.5 mol % [AuClPPh_3_], 2.5 mol % AgOTf, 10 mol % PTSA, CH_2_Cl_2_, sealed tube, 130 °C, 1.5 h.

Thus, it is possible to suppress the formation of the 1,3-oxazinan-2-one ring by performing the reaction at higher temperature, yielding the 1,3-oxazin-2-one as the exclusive product. A general trend can be deduced on the basis of these results: heterocycle **4** is the thermodynamically controlled product while heterocycle **3** is the kinetically controlled product [71–73]. Probably, double-bond migration in compounds **3** results in the formation of the 1,3-oxazin-2-one **4**. In order to verify the role of the Au(I) catalyst in the double-bond migration process, we set up two experiments. Heating a mixture of **3a** with Au(OTf)PPh_3_ at a loading of 2.5 mol % in dichloromethane for 1.5 h at 130 °C resulted in full conversion into **4a**. Running the same reaction in the absence of any catalyst resulted in 30% conversion after two days, as determined by ^1^H NMR. Treatment of 1,3-oxazinan-2-one **3a** with 5 mol % TfOH in CH_2_Cl_2_ at room temperature did not proceed to give an appreciable amount of 3,4-dihydro-2*H*-1,3-oxazin-2-one **4a** after 2 h. This indicates that the Au(I) catalyst might participate in the double-bond migration process; being a possible intermediate, the π-allyl complex **5** is depicted in Scheme 3 [74]. Despite that, the isomerization process can be also viewed as an intramolecular 1,3-H shift assisted by gold.

**Scheme 4:**
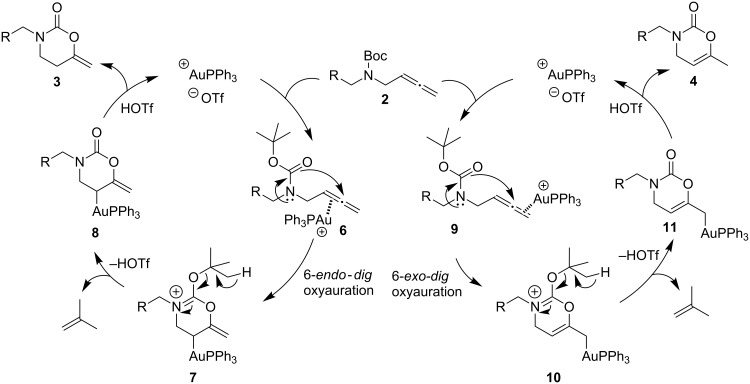
Mechanistic explanation for the gold-catalyzed oxycyclization reactions of allenic carbamates **2** into 6-methylene-1,3-oxazinan-2-ones **3** or into 3-substituted-3,4-dihydro-2*H*-1,3-oxazin-2-ones **4**.

A possible pathway for the gold-catalyzed achievement of heterocycles **3** from allenyl-tethered carbamates **2** may initially involve the formation of a complex **6** through coordination of the gold salt to the proximal allenic double bond. Next, chemo- and regioselective 6-*endo*-*dig* oxyauration of the carbamate carbonyl moiety forms species **7**. Attack of the carbamate carbonyl group occurs as a result of the stability of the intermediate ammonium cation type **7**. Loss of proton linked to 2-methylprop-1-ene release [75–78], generates neutral species **8**, which followed by protonolysis of the carbon–gold bond affords 6-methylene-1,3-oxazinan-2-ones **3** with concurrent regeneration of the gold catalyst (Scheme 4, left catalytic cycle). In line with the above mechanistic proposal, the easy breakage of the *tert*-butyl group at species **7** is essential for the formation of 1,3-oxazinan-2-ones **3**. Besides, the replacement of the *tert*-butyl group in allenic carbamates **2** by other alkyl functions, such as methyl, did not allow the preparation of heterocycles **3**. In addition to the double-bond isomerization that transforms products **3** into the thermodynamically more favored compounds **4**, a mechanistic scenario involving the initial coordination of the gold to the distal allenic double bond leading to complex **9**, followed by a 6-*exo*-*dig* oxyauration is likely for the achievement of 1,3-oxazin-2-ones **4** from allenic carbamates **2** (Scheme 4, right-hand catalytic cycle).

**Figure 1:**
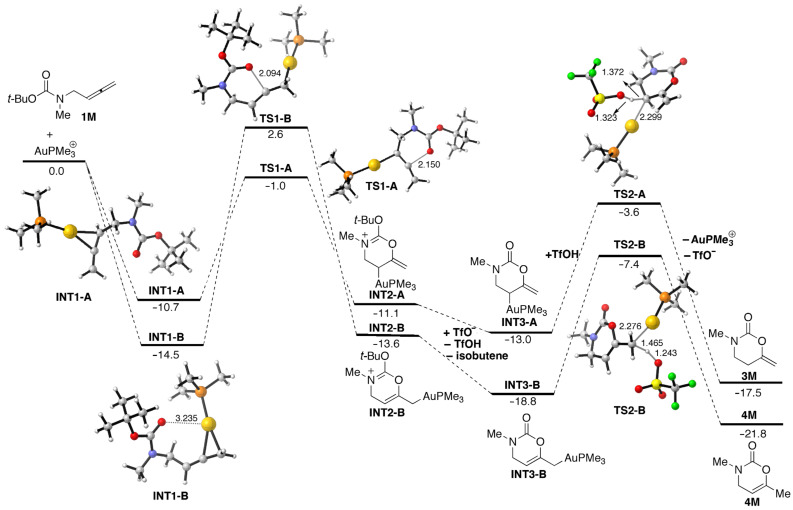
Computed reaction profile for the reaction of AuPMe_3_^+^ and **1M**. Numbers indicate the corresponding PCM-corrected Δ*G*_298_ energies (in kcal/mol) using dichloromethane as solvent. Bond distances are given in angstroms. All data have been computed at the PCM-M06/def2-SVP//B3LYP/def2-SVP level.

Density functional theory (DFT) calculations (see Supporting Information File 1) have been carried out at the PCM-M06/def2-SVP//B3LYP/def2-SVP level to gain more insight into the reaction mechanism of the above discussed gold-catalyzed divergent oxycyclization reaction. The corresponding computed reaction profiles of the model allene **1M** with the model gold catalyst AuPMe_3_(OTf) are shown in Figure 1, which gathers the respective free energies (computed at 298 K) in CH_2_Cl_2_ solution.

As initially envisaged, two different coordination modes of the metal fragment to the allenic double bond of **1M**, i.e., distal versus proximal, are possible. Our calculations indicate that the distal coordination leading to **INT1-B** is favored over the proximal coordination mode, which forms **INT1-A** (ΔΔ*G* = 3.8 kcal/mol). This is mainly due to the presence of a two-electron stabilizing interaction established by donation of electronic density from the lone pair of the oxygen atom of the carbonyl moiety to a vacant d atomic orbital of the gold atom in **INT1-B** [79]. Both complexes can undergo the corresponding oxyauration cyclization reaction. Thus, **INT1-A** is converted into **INT2-A** in a slightly exergonic process (Δ*G*_R,298_ = −0.4 kcal/mol) through the saddle point **TS1-A**, which is associated with the 6-*endo*-*dig* cyclization. Similarly, **INT1-B** is transformed into **INT2-B** in a slightly endergonic process (Δ*G*_R,298_ = +0.9 kcal/mol) via **TS1-B**, associated with the 6-*exo*-*dig* cyclization reaction.

From the data in Figure 1, it becomes obvious that the 6-*endo*-*dig* transformation is kinetically favored over the 6-*exo*-*dig* reaction in view of the computed lower activation barrier of the former process (ΔΔ*G*^≠^_298_ = +7.4 kcal/mol). However, the cyclic reaction product **INT2-B** is thermodynamically more stable than the counterpart **INT2-A** (ΔΔ*G* = 2.5 kcal/mol), which is in agreement with the experimental findings (see above). The next step of the process involves the TfO^−^ promoted elimination of isobutene to form the corresponding **INT3** complexes. The driving force of this process is clearly related to the thermodynamically favored release of isobutene (Δ*G*_R,298_ = −1.9 and −5.2 kcal/mol from **INT3-A** and **INT3-B**, respectively). Finally, the protonolysis reaction of the carbon–gold bond by TfOH renders the final products **3M** and **4M** regenerating the catalyst. This step occurs through the transition states **TS2-A** and **TS2-B**, respectively, in an exergonic transformation (Δ*G*_R,298_ = −4.5 and −3.0 kcal/mol from **INT3-A** and **INT3-B**, respectively). Again, the data in Figure 1 indicate that the final product **4M** is thermodynamically more stable than **3M**, which is in line with the experimentally observed conversion of **3a** into **4a** by heating in the presence and also in the absence of the gold-catalyst. From the computed reaction profile, it can be concluded that the observed divergent cyclization finds its origin in the initial 6-*endo* versus 6-*exo* oxyauration reaction steps, with the former being kinetically favored whereas the latter is thermodynamically favored. At this point, it cannot be safely discarded that the formation of the thermodynamically more stable 6-*exo*-*dig* products is the result of the simple thermally promoted isomerization of the less stable 6-*endo*-*dig* species.

## Conclusion

In conclusion, efficient gold-catalyzed synthetic routes to 1,3-oxazinan-2-one and 1,3-oxazin-2-one derivatives from easily accessible allenic carbamates under mild conditions have been reported. The oxycyclization reactions were found to proceed with complete control of regioselectivity. The mechanism of these processes has additionally been investigated by a computational study showing that heterocycles **3** are the kinetically controlled products whereas heterocycles **4** are thermodynamically favored.

## Experimental

### General Information

^1^H NMR and ^13^C NMR spectra were recorded on 700, 500, 300, or 200 MHz spectrometers. NMR spectra were recorded in CDCl_3_ solutions, except were otherwise stated. Chemical shifts are given in parts per million relative to TMS (^1^H, 0.0 ppm) or CDCl_3_ (^13^C, 76.9 ppm). Low- and high-resolution mass spectra were taken on a QTOF LC–MS spectrometer using the electronic impact (EI) or electrospray modes (ES) unless otherwise stated. Specific rotation [α]_D_ is given in 10^−1^ deg cm^2^ g^−1^ at 20 °C, and the concentration (*c*) is expressed in grams per 100 mL. All commercially available compounds were used without further purification.

### Typical procedure for the Au(I)-catalyzed preparation of 1,3-oxazin-2-ones, **4**

[AuClPPh_3_] (0.00475 mmol), AgOTf (0.00475 mmol), and *p*-toluenesulfonic acid (0.019 mmol) were sequentially added to a stirred solution of the allenic carbamate **2a** (50 mg, 0.19 mmol) in dichloromethane (1.9 mL). The resulting mixture was heated in a sealed tube at 130 °C until disappearance of the starting material (TLC, 1.5 h). The reaction was allowed to cool to room temperature and filtered through a pack of celite. The filtrate was extracted with dichloromethane (3 × 5 mL), and the combined extracts were washed twice with brine. The organic layer was dried (MgSO_4_), concentrated under reduced pressure, and purified by flash column chromatography on silica gel (hexanes/ethyl acetate 4:1) to afford product **4a** (27 mg, 70%) as a colorless oil. ^1^H NMR (300 MHz, CDCl_3_, 25 °C) δ 7.33 (m, 2H), 4.76 (m, 1H), 4.58 (s, 2H), 3.68 (dq, *J =* 3.2, 1.9 Hz, 2H), 1.86 (td, *J =* 1.9, 1.2 Hz, 3H); ^13^C NMR (75 MHz, CDCl_3_, 25 °C) δ 150.9, 148.1, 135.6, 128.7, 128.2, 127.9, 94.4, 52.1, 44.6, 18.3; IR (CHCl_3_) ν: 1685 cm^−1^; HRMS–ES (*m*/*z*): [M]^+^ calcd for C_12_H_13_NO_2_, 203.0946; found, 203.0952.

## Supporting Information

File 1 Experimental details, analytical data of new compounds, copies of ^1^H NMR and ^13^C NMR spectra and computational details.
