# Supplementary material for: Gold-catalyzed oxycyclization of allenic carbamates: expeditious synthesis of 1,3-oxazin-2-ones
Source: Beilstein J Org Chem. 2013 Apr 26;9:818–26. doi: 10.3762/bjoc.9.93 (PMC3678844; doi:10.3762/bjoc.9.93)
Supplement: File 1 — Experimental details, analytical data of new compounds, copies of 1H NMR and 13C NMR spectra and computational details. [file Beilstein_J_Org_Chem-09-818-s001.pdf]

Supporting Information
for
Gold-catalyzed oxycyclization of allenic carbamates: expeditious synthesis of 1,3-oxazin-2-ones

Benito Alcaide*[1], Pedro Almendros*[2], M. Teresa Quirós[1] and Israel Fernández[3]

Address: [1]Grupo de Lactamas y Heterociclos Bioactivos, Departamento de Química Orgánica I, Unidad Asociada al CSIC, Facultad de Química, Universidad Complutense de Madrid, 28040-Madrid, Spain, [2]Instituto de Química Orgánica General (IQOG), Consejo Superior de Investigaciones Científicas (CSIC), Juan de la Cierva 3, 28006-Madrid, Spain and [3]Departamento de Química Orgánica I, Facultad de Química, Universidad Complutense de Madrid, 28040-Madrid, Spain

Email: Benito Alcaide - alcaideb@quim.ucm.es and Pedro Almendros - Palmendros@iqog.csic.es

* Corresponding author

Experimental details, analytical data of new compounds, copies of ^{1}H NMR and ^{13}C NMR spectra and computational details

Experimental section S2
Computational details.. S17
Figure 1S.. S26
^{1}H NMR and ^{13}C NMR spectra S27
References.. S64

Experimental section

General methods: ^{1}H NMR and ^{13}C NMR spectra were recorded on 700, 500, 300, or 200 MHz spectrometers. NMR spectra were recorded in $CDCl_3$ solutions, except were otherwise stated. Chemical shifts are given in ppm relative to TMS (^{1}H, 0.0 ppm) or $CDCl_3$ (^{13}C, 76.9 ppm). Low- and high-resolution mass spectra were taken on a QTOF LC–MS spectrometer using the electronic impact (EI) or electrospray modes (ES) unless otherwise stated. Specific rotation $[\alpha]_D$ is given in 10^{-1} deg cm^{2} g^{-1} at 20 °C, and the concentration (*c*) is expressed in grams per 100 mL. All commercially available compounds were used without further purification.

General procedure for the synthesis of *tert*-butyl (prop-2-ynyl)carbamates 1a–g. A solution of propargylamine (1.0 mmol) in CH_2Cl_2 (0.1 mL) was added to a well stirred suspension of the corresponding aldehyde (1.0 mmol) and $MgSO_4$ (8.0 mmol) in CH_2Cl_2 (1.0 mL) at RT. After disappearance of the starting material (TLC, typically 20 h), the solid was removed by filtration. The organic filtrate was concentrated under reduced pressure and used for the next step.

Sodium borohydride (2.0 mmol) was slowly added over a solution of the appropriate propargylic imine (1.0 mmol) in methanol (10.0 mL) at –20 °C. The reaction mixture was stirred at –20 °C until disappearance of the starting material (TLC, typically 30 min). The crude was diluted with acetone (10.0 mL). The resulting mixture was filtered through a pad of celite and the filtrate was extracted with dichloromethane (4 × 20 mL). The organic layer was washed with brine, dried ($MgSO_4$), concentrated under reduced pressure, and used for the next step.

A solution of di-*tert*-butyl dicarbonate (1.1 mmol) in CH_2Cl_2 (1.0 mL) was added to a solution of the appropriate propargylic amine (1.0 mmol) and triethylamine (1.1 mmol) in CH_2Cl_2 (5.0 mL) at 0 °C. The mixture was stirred until disappearance of the starting material (TLC, 2–20 h) at RT. Afterwards the resulting mixture was extracted with DCM (4 × 20 mL), washed with brine, dried ($MgSO_4$) and concentrated under reduced pressure. Chromatography of the residue using ethyl acetate/hexanes or ethyl acetate/dichloromethane mixtures gave analytically pure compounds **1**. Spectroscopic and analytical data for *tert*-butyl (prop-2-ynyl)carbamates **1a–g** follow.

1a. From 500 mg (4.71 mmol) of the appropriate aldehyde, and after chromatography of the residue using hexanes/ethyl acetate (4:1) as eluent gave compound **1a** (589 mg, 51%) as a pale

yellow oil; ^{1}H NMR (300 MHz, $CDCl_3$, 25 °C): δ = 1.50 (s, 9H, $3CH_3$ Boc), 2.21 (t, 1H, *J* = 2.5 Hz, C≡C*H*), 4.02 (br s, 2H, NCH_2), 4.56 (s, 2H, CH_2-C≡CH), 7.32 (m, 5H, ArH); ^{13}C NMR (75 MHz, $CDCl_3$, 25 °C): δ = 155.0 (C=O), 136.6 (C^q), 128.5 (4CH PMP), 127.4 (CH Ph), 85.2 (≡CH), 80.6 (C^q Boc), 79.3 (≡C), 49.2 (NCH_2), 35.0 (NCH_2), 28.4 ($3CH_3$ Boc); IR ($CHCl_3$): ν = 2362 (≡CH), 1700 (C=O) cm^{-1}; HRMS (ES): calcd for $C_{14}H_{15}NO$ $[M]^+$: 213.1154; found: 213.1157.

1b. From 1.0 g (7.35 mmol) of the appropriate aldehyde, and after chromatography of the residue using hexanes/ethyl acetate (6:1) as eluent gave compound **1b** (1.44 g, 71%) as a pale yellow oil; ^{1}H NMR (300 MHz, $CDCl_3$, 25 °C): δ = 1.50 (s, 9H, $3CH_3$ Boc), 2.21 (t, 1H, *J* = 2.3 Hz, C≡C*H*), 3.80 (s, 3H, OCH_3), 3.96 (br s, 2H, NCH_2), 4.49 (s, 2H, CH_2-C≡CH), 6.86 (d, 2H, *J* = 8.7 Hz, ArH), 7.21 (d, 2H, *J* = 8.4 Hz, ArH); ^{13}C NMR (75 MHz, $CDCl_3$, 25 °C): δ = 158.9 (O-C^q), 154.9 (C=O), 129.4 (C^q), 129.2 (2 CH PMP), 113.9 (2CH PMP), 80.4 (C^q Boc), 79.4 (≡CH), 71.5 (≡C), 55.2 (OCH_3), 48.4 (NCH_2), 34.8 (NCH_2), 28.3 ($3CH_3$ Boc); IR ($CHCl_3$): ν = 2360 (≡CH), 1698 (C=O) cm^{-1}; HRMS (ES): calcd for $C_{16}H_{21}NO_3$ $[M]^+$: 275.1521; found: 275.1507.

(+)-1c. From 396 mg (3.05 mmol) of the appropriate aldehyde, and after chromatography of the residue using dichloromethane/ethyl acetate (9:1) as eluent gave compound (+)-**1c** (599 mg, 77%) as a pale yellow oil; $[\alpha]_D$ +1.60 (*c* 1.4, $CHCl_3$); ^{1}H NMR (300 MHz, $CDCl_3$, 25 °C): δ = 1.38 (s, 3H, CH_3), 1.45 (s, 3H, CH_3), 1.50 (s, 9H, $3CH_3$ Boc), 2.23 (t, 1H, *J* = 2.2 Hz, C≡C*H*), 3.5 (m, 2H, NCH_2), 3.71 (m, 1H, OC*H*H), 4.07 (dd, 1H, *J* = 8.4, 6.2 Hz, OCH*H*), 4.20 (br s, 1H, NC*H*H-alkyne), 4.32 (m, 1H, NCH*H*-alkyne); ^{13}C NMR (75 MHz, $CDCl_3$, 25 °C): δ = 157.1 (C=O), 109.3 (C^q acetonide), 86.3 (≡CH), 80.6 (C^q Boc), 75.6 (≡C), 73.9 (OCH), 67.2 (OCH_2), 48.3 (NCH_2), 37.5 (NCH_2-alkyne), 28.3 ($3CH_3$ Boc), 26.8 (CH_3), 25.5 (CH_3); IR ($CHCl_3$): ν = 2358 (≡CH), 1696 (C=O) cm^{-1}; HRMS (ES): calcd for $C_{14}H_{23}NO_4$ $[M]^+$: 269.1627; found: 269.1636.

(+)-1d. From 500 mg (2.12 mmol) of the appropriate aldehyde, and after chromatography of the residue using hexanes/ethyl acetate (5:1) as eluent gave compound (+)-**1d** (437 mg, 57%) as a pale yellow oil; $[\alpha]_D$ +37.2 (*c* 0.8, $CHCl_3$); ^{1}H NMR (300 MHz, $CDCl_3$, 25 °C): δ = 1.52 (s, 9H, 3 CH_3 Boc), 2.18 (s, 1H, C≡C*H*), 3.40 (br s, 1H, NC*H*H), 3.68 (s, 3H, OCH_3), 3.79 (s, 3H, OCH_3), 3.96 (d, 1H, *J* = 4.1 Hz, C*H*H-C≡CH), 4.01 (d, 1H, *J* = 4.1 Hz, CH*H*-C≡CH), 4.09 (br s, 1H,

NCH*H*), 4.47 (br s, 1H, H4), 4.59 (d, 1H, *J* = 5.1 Hz, H3), 6.88 (d, 2H, *J* = 8.1 Hz, ArH), 7.47 (m, 2H, ArH); ^{13}C NMR (75 MHz, $CDCl_3$, 25 °C): δ = 164.5 (C=O lactam), 154.8 (O-C^q), 153.0 (C=O Boc), 130.4 (C^q), 118.7 (2CH ArH), 114.5 (2CH ArH), 82.7 (≡CH), 80.2 (C-H3), 80.2 (C^q), 75.3 (≡C), 59.2 (OCH_3), 55.5 (OCH_3), 53.3 (C-H4), 44.7 (2NCH_2), 28.4 (3CH_3 Boc); IR ($CHCl_3$): ν = 1745 (C=O lactam), 2354 (≡CH), 1693 (C=O) cm^{-1}; HRMS (ES): calcd for $C_{19}H_{24}N_2O_5$ $[M]^+$: 360.1685; found: 360.1691.

(+)-1e. From 912 mg (4.16 mmol) of the appropriate aldehyde, and after chromatography of the residue using hexanes/ethyl acetate (2:1) as eluent gave compound (+)-**1e** (860 mg, 60%) as a colorless oil; $[\alpha]_D$ +39.6 (*c* 0.7, $CHCl_3$); ^{1}H NMR (300 MHz, $CDCl_3$, 25 °C): δ = 1.45 (s, 9H, 3 CH_3 Boc), 2.15 (t, 1H, *J* = 2.1 Hz, C≡C*H*), 3.34 (dd, 1H, *J* = 14.7, 6.3 Hz, NC*H*H), 3.59 (s, 3H, OCH_3), 3.68 (dd, 1H, *J* = 14.9, 4.9 Hz, NCH*H*), 3.86 (m, 3H, CH_2-C≡CH + H4), 4.14 (br s, 1H, C*H*H-Ph), 4.48 (d, 1H, *J* = 3.9 Hz, H3), 4.66 (br s, 1H, CH*H*-Ph), 7.31 (m, 5H, ArH); ^{13}C NMR (75 MHz, $CDCl_3$, 25 °C): δ = 167.6 (C=O lactam), 155.1 (C=O Boc), 136.5 (C^q), 128.8 (2CH Ph), 128.4 (2CH Ph), 128.2 (CH Ph), 83.6 (≡CH), 80.8 (C-H3), 80.5 (C^q), 76.0 (≡C), 65.8(CH_2-Ph), 64.5 (C-H4), 59.1 (OCH_3), 45.5 (NCH_2), 44.5 (NCH_2), 28.3 (3CH_3 Boc); IR ($CHCl_3$): ν = 1746 (C=O lactam), 2355 (≡CH), 1692 (C=O) cm^{-1}; HRMS (ES): calcd for $C_{20}H_{26}N_2O_4$ $[M]^+$: 358.1893; found: 358.1887.

(+)-1f. From 640 mg (2.32 mmol) of the appropriate aldehyde, and after chromatography of the residue using hexanes/ethyl acetate (6:1) as eluent gave compound (+)-**1f** (670 mg, 72%) as a colorless oil; $[\alpha]_D$ +56.0 (*c* 1.6, $CHCl_3$); ^{1}H NMR (300 MHz, $CDCl_3$, 25 °C): δ = 1.52 (s, 9H, 3 CH_3 Boc), 1.81 (s, 3H, Me), 2.18 (2, 1H, C≡C*H*), 3.48 (br s, 1H, NC*H*H), 3.79 (s, 1H, OCH_3), 3.98 (m, 1H, H4), 4.01 (dd, 1H, *J* = 14.8, 4.1, NCH*H*), 4.17 (br s, 1H, NC*H*H-alkyne), 4.20 (d, 1H, *J* = 12.2 Hz, OC*H*H), 4.36 (d, 1H, *J* = 12.3 Hz, OCH*H*), 4.49 (br s, 1H, NCH*H*-alkyne), 4.73 (d, 1H, *J* = 5.0 Hz, H3), 4.98 (s, 1H, =CHH), 5.07 (s, 1H, =CHH), 6.89 (m, 2H, ArH), 7.49 (m, 2H, ArH); ^{13}C NMR (75 MHz, $CDCl_3$, 25 °C): δ = 164.6 (C=O lactam), 155.5 (C=O Boc + O-C^q), 141.0 (=C^q), 130.5 (C^q), 118.7 (2CH PMP), 114.5 (2CH PMP), 113.3 (=CH_2), 82.6 (≡CH), 80.4 (C-H3), 79.5 (C^q), 76.0 (≡C), 75.1 (OCH_2), 63.3 (C-H4), 55.5 (OCH_3), 44.8 (NCH_2), 34.7 (NCH_2), 28.4 (3CH_3 Boc), 19.6 (CH_3); IR ($CHCl_3$): ν = 1747 (C=O lactam), 2356 (≡CH), 1694 (C=O) cm^{-1}; HRMS (ES): calcd for $C_{23}H_{30}N_2O_5$ $[M]^+$: 414.2155; found: 414.2148.

1g. From 500 mg (3.73 mmol) of the appropriate aldehyde, and after chromatography of the residue using hexanes/ethyl acetate (6:1) as eluent gave compound **1g** (624 mg, 38%) as a pale yellow oil; ^{1}H NMR (300 MHz, $CDCl_3$, 25 °C): δ = 1.49 (s, 18H, $6CH_3$ Boc), 2.21 (t, 2H, J = 2.4 Hz, 2C≡C*H*), 4.00 (br s, 4H, $2NCH_2$), 4.54 (s, 4H, 2C*H*$_2$-C≡CH), 7.23 (s, 4H, ArH); ^{13}C NMR (75 MHz, $CDCl_3$, 25 °C): δ = 155.1 (2C=O), 136.7 (2C^q), 128.0 (4CH Ar), 85.2 (2≡CH), 80.6 (2C^q Boc), 79.3 (2≡C), 49.5 ($2NCH_2$), 34.9 ($2NCH_2$), 28.4 ($6CH_3$ Boc); IR ($CHCl_3$): ν = 2365 (≡CH), 1701 (C=O) cm^{-1}; HRMS (ES): calcd for $C_{24}H_{32}N_2O_4$ $[M]^+$: 412.2362; found: 412.2355.

1j. The Ohira–Bestmann reagent (1.76 mmol) was added to a well stirred suspension of 3-bromo-1*H*-indole-2-carbaldehyde (330 mg, 1.47 mmol) and K_2CO_3 (2.94 mmol) in anhydrous methanol (22 mL) at RT. After disappearance of the starting material (TLC, 15 h), the solid was removed by filtration, and the filtrate was diluted with diethyl ether (15 mL). The organic extract was washed with $NaHCO_3$ (5% aq.), dried ($MgSO_4$), and concentrated under reduced pressure. Chromatography of the residue using hexanes/ethyl acetate (5:1) as eluent gave 3-bromo-2-ethynyl-1*H*-indole (228 mg, 63%) as a pale brown oil; ^{1}H NMR (300 MHz, $CDCl_3$, 25 °C): δ = 3.59 (s, 1H, ≡CH), 7.23 (m, 1H, indole), 7.32 (d, 2H, J = 3.6 Hz, indole), 7.56 (dd, 1H, J = 7.9, 0.8 Hz, indole), 8.34 (br s, 1H, NH); ^{13}C NMR (75 MHz, $CDCl_3$, 25 °C): δ = 135.0 (C^q), 126.6 (C^q), 125.0 (CH indole), 121.3 (CH indole), 119.7 (CH indole), 117.3 (C^q), 111.2 (CH indole), 99.1 (C^q), 84.6 (≡CH), 74.25 (≡C); HRMS (ES): calcd for $C_{15}H_{14}BrNO_2$ $[M]^+$: 319.0208; found: 319.0200.

A solution of di-*tert*-butyl dicarbonate (0.902 mmol) in acetonitrile (0.82 mL) was added to a solution of 3-bromo-2-ethynyl-1*H*-indole (180 mg, 0.82 mmol) and DMAP (0.902 mmol) in acetonitrile (4.1 mL) at 0 °C. The mixture was stirred until disappearance of the starting material (TLC, 2 h) at RT. Afterwards the resulting mixture was extracted with ethyl acetate (4 × 15 mL), washed with brine, dried ($MgSO_4$) and concentrated under reduced pressure. Chromatography of the residue using hexanes/ethyl acetate (4:1) as eluent gave compound **1j** (224 mg, 86%) as a pale brown solid; mp 177–178 °C; ^{1}H NMR (300 MHz, $CDCl_3$, 25 °C): δ = 1.70 (s, 9H, 3CH3 Boc), 3.79 (s, 1H, ≡CH), 7.34 (m, 1H, CH indole), 7.43 (m, 1H, CH indole), 7.54 (ddd, 1H, J = 7.7, 1.5, 0.8 Hz, CH indole), 8.16 (dt, 1H, J = 8.3, 0.9 Hz, CH indole); ^{13}C NMR (75 MHz, $CDCl_3$, 25 °C): δ = 148.8 (C=O), 135.0 (C^q), 127.9 (C^q), 127.1 (CH indole), 123.9 (CH indole), 119.9 (CH indole),

115.7 (CH indole), 109.0 (C^q), 87.8 (≡CH), 85.5 (C^q Boc), 74.8 (≡C), 28.1 ($3CH_3$ Boc); IR ($CHCl_3$): ν = 2356 (≡CH), 1692 (C=O) cm^{-1}.

General procedure for the synthesis of allenic carbamates 2a–i. CuBr (0.50 mmol), $(CH_2O)_n$ (2.55 mmol), and diisopropylamine (1.80 mmol) were sequentially added under an argon atmosphere to a solution of the corresponding alkynylcarbamate **1** (1.0 mmol) in dioxane (5.0 mL). The mixture was stirred under reflux until disappearance of the starting material (TLC, typically 1 h). Afterwards, it was cooled to RT and diluted with water (5.0 mL). The resulting mixture was extracted with ethyl acetate (4 × 15 mL), washed with brine, dried ($MgSO_4$) and concentrated under reduced pressure. Chromatography of the residue using ethyl acetate/hexanes mixtures gave analytically pure compounds **2**. Spectroscopic and analytical data for allenic carbamates **2a–i** follow.

2a. From 395 mg (1.61 mmol) of the (prop-2-ynyl)carbamate **1a**, and after chromatography of the residue using hexanes/ethyl acetate (5:1) as eluent gave compound **2a** (238 mg, 57%) as a colorless oil; ^{1}H NMR (300 MHz, $CDCl_3$, 25 °C): δ = 1.51 (s, 9H, $3CH_3$ Boc), 3.79 (br s, 2H, NCH_2), 4.48 (s, 2H, CH_2-C=·=), 4.78 (dt, 2H, *J* = 6.6, 2.6 Hz, =·=CH_2), 5.12 (br s, 1H, CH=·=), 7.34 (m, 5H, ArH); ^{13}C NMR (75 MHz, $CDCl_3$, 25 °C): δ = 202.1 (=·=), 158.9 (C=O), 130.8 (C^q), 128.4 (4CH Ph), 127.1 (CH Ph), 86.9 (CH=·=), 80.0 (C^q Boc), 76.1 (=·=CH_2), 49.4 (NCH_2), 44.9 (NCH_2), 28.4 ($3CH_3$ Boc); IR ($CHCl_3$): ν = 1955 (=·=), 1692 (C=O) cm^{-1}; HRMS (ES): calcd for $C_{16}H_{21}NO_2$ $[M]^+$: 259.1572; found: 259.1579.

2b. From 664 mg (2.41 mmol) of the (prop-2-ynyl)carbamate **1b**, and after chromatography of the residue using hexanes/ethyl acetate (4:1) as eluent gave compound **2b** (624 mg, 89%) as a pale yellow oil; ^{1}H NMR (300 MHz, $CDCl_3$, 25 °C): δ = 1.49 (s, 9H, $3CH_3$ Boc), 3.76 (br s, 2H, NCH_2), 3.80 (s, 3H, OCH_3), 4.38 (s, 2H, CH_2-C=·=), 4.75 (dt, 2H, *J* = 6.6, 2.8 Hz, =·=CH_2), 5.08 (br s, 1H, CH=·=), 6.86 (d, 2H, *J* = 8.7 Hz, ArH), 7.19 (d, 2H, *J* = 8.1 Hz, ArH); ^{13}C NMR (75 MHz, $CDCl_3$, 25 °C): δ = 206.3 (=·=), 158.8 (O-C^q), 158.8 (C=O), 130.3 (C^q), 129.4 (2CH PMP), 113.9 (2CH PMP), 86.9 (CH=·=), 79.8 (C^q Boc), 76.1 (=·=CH_2), 55.2 (OCH_3), 48.9 (NCH_2), 44.6 (NCH_2), 28.4 ($3CH_3$ Boc); IR ($CHCl_3$): ν = 1954 (=·=), 1692 (C=O) cm^{-1}; HRMS (ES): calcd for $C_{17}H_{23}NO_3$ $[M]^+$: 289.1678; found: 289.1675.

(+)-2c. From 329 mg (1.22 mmol) of the (prop-2-ynyl)carbamate (+)-**1c**, and after chromatography of the residue using hexanes/ethyl acetate (4:1) as eluent gave compound (+)-**2c** (238 mg, 69%) as a colorless oil; $[\alpha]_D$ +14.6 (*c* 1.2, in $CHCl_3$); ^1H NMR (300 MHz, $CDCl_3$, 25 °C): δ = 1.34 and 1.41 (s, each 3H, $2CH_3$), 1.45 (s, 9H, $3CH_3$ Boc), 3.39 (m, 2H, NCH_2), 3.67 (m, 1H, NCH*H*-allene), 3.93 (m, 2H, OCH*H* + NC*H*H-allene), 4.02 (dd, 1H, *J* = 8.3, 6.2 Hz, OC*H*H), 4.25 (br s, 1H, OCH), 4.77 (dt, 2H, *J* = 6.6, 2.8 Hz, CH=·=CH_2), 5.12 (q, 1H, *J* = 6.4 Hz, C*H*=·=CH_2); ^{13}C NMR (75 MHz, $CDCl_3$, 25 °C): δ = 208.8 (=·=), 151.8 (C=O), 109.1 (C^q acetonide), 87.1 (*C*H=·=CH_2), 79.9 (C^q Boc), 76.3 (CH=·=*C*H_2), 75.3 (OCH), 67.3 (OCH_2), 49.1 (NCH_2), 47.3 (NCH_2-allene), 28.4 ($3CH_3$ Boc), 26.8 (CH_3), 25.5 (CH_3); IR ($CHCl_3$): ν = 1957 (=·=), 1694 (C=O) cm^{-1}; HRMS (ES): calcd for $C_{15}H_{25}NO_4$ $[M]^+$: 283.1784; found: 283.1786.

(+)-2d. From 200 mg (0.53 mmol) of the (prop-2-ynyl)carbamate (+)-**1d**, and after chromatography of the residue using hexanes/ethyl acetate (4:1) as eluent gave compound (+)-**2d** (168 mg, 81%) as a pale yellow oil; $[\alpha]_D$ +15.6 (*c* 0.7, $CHCl_3$); ^1H NMR (300 MHz, $CDCl_3$, 25 °C): δ = 1.49 (s, 9H, $3CH_3$ Boc), 3.35 (dd, 1H, *J* = 14.4, 7.2 Hz, NC*H*H), 3.63 (s, 3H, OCH_3), 3.74 (m, 1H, NCH*H*), 3.77 (s, 3H, OCH_3), 3.78 (m, 1H, CH*H*-C=·=), 3.82 (d, 1H, *J* = 4.4 Hz, H4), 4.46 (m, 1H, C*H*H-C=·=), 4.56 (d, 1H, *J* = 5.1 Hz, H3), 4.76 (m, 2H, =·=CH_2), 5.03 (br s, 1H, CH=·=), 6.85 (d, 2H, *J* = 8.3 Hz, ArH), 7.44 (m, 2H, ArH); ^{13}C NMR (75 MHz, $CDCl_3$, 25 °C): δ = 208.5 (=·=), 164.5 (C=O lactam), 155.7 (C=O Boc), 130.4 (C^q), 118.7 (2CH PMP), 114.3 (2CH PMP), 87.4 (CH=·=), 82.5 (C-H3), 80.5 (O-C^q), 79.9 (=·=CH_2), 59.1 (OCH_3), 55.9 (C-H4), 55.4 (OCH_3), 47.4 (NCH_2), 45.1 (NCH_2), 28.4 ($3CH_3$ Boc); IR ($CHCl_3$): ν = 1957 (=·=), 1754 (C=O), 1694 (C=O) cm^{-1}; HRMS (ES): calcd for $C_{21}H_{28}N_2O_5$ $[M]^+$: 388.1998; found: 388.2021.

(+)-2e. From 209 mg (0.58 mmol) of the (prop-2-ynyl)carbamate (+)-**1e**, and after chromatography of the residue using hexanes/ethyl acetate (4:1) as eluent gave compound (+)-**2e** (154 mg, 71%) as a colorless oil; $[\alpha]_D$ +13.9 (*c* 1.1, in $CHCl_3$); ^1H NMR (300 MHz, $CDCl_3$, 25 °C): δ = 1.45 (s, 9H, $3CH_3$ Boc), 3.28 (dd, 1H, *J* = 14.3, 5.3 Hz, NC*H*H), 3.57 (s, 3H, OCH_3), 3.57 (m, 1H, NCH*H*), 3.78 (m, 3H, NC*H*$_2$-C=·= + H4), 4.12 (m, 1H, C*H*H-Ph), 4.45 (d, 1H, *J* = 4.2 Hz, H3), 4.67 (m, 1H, CH*H*-Ph), 4.75 (m, 2H, =·=CH_2), 5.04 (br s, 1H, CH=·=CH_2), 7.31 (m, 5H, ArH); ^{13}C NMR (75 MHz, $CDCl_3$, 25 °C): δ = 205.3 (=·=), 169.8 (C=O lactam), 155.9 (C=O Boc), 135.4 (C^q), 128.8 (2CH Ph), 128.5 (2CH Ph), 128.0 (CH Ph), 91.8 (CH=·=), 84.5 (O-C^q), 83.5 (C-

H3), 76.7 (=·=CH_2), 64.8 (C-H4), 59.1 (OCH_3), 46.2 (NCH_2), 44.6 (NCH_2), 28.4 ($3CH_3$ Boc); IR ($CHCl_3$): ν = 1956 (=·=), 1755 (C=O), 1693 (C=O) cm^{-1}; HRMS (ES): calcd for $C_{21}H_{28}N_2O_4$ $[M]^+$: 372.2049; found: 372.2041.

(+)-2f. From 359 mg (0.87 mmol) of the (prop-2-ynyl)carbamate (+)-**1f**, and after chromatography of the residue using hexanes/ethyl acetate (4:1) as eluent gave compound (+)-**2f** (237 mg, 64%) as a colorless oil; $[\alpha]_D$ +105.5 (*c* 0.5, $CHCl_3$); ^1H NMR (300 MHz, $CDCl_3$, 25 °C): δ = 1.50 (s, 9H, $3CH_3$ Boc), 1.79 (s, 3H, Me), 3.42 (dd, 1H, *J* = 14.6, 7.4 Hz, NC*H*H), 3.79 (s, 3H, OCH_3), 3.84 (m, 1H, NC*H*H-C=·= + NCH*H*), 3.85 (d, 1H, *J* = 4.2 Hz, H4), 4.17 (d, 1H, *J* = 12.5 Hz, OC*H*H), 4.31 (d, 1H, *J* = 12.4 Hz, OCH*H*), 4.50 (m, 1H, NCH*H*-C=·=), 4.72 (d, 1H, *J* = 5.2 Hz, H3), 4.77 (m, 2H, =·=CH_2), 4.97 (br s, 1H, =C*H*H), 5.06 (br s, 1H, C*H*=·=CH_2 + =CH*H*), 6.88 (d, 2H, *J* = 7.9 Hz, ArH), 7.46 (m, 2H, ArH); ^{13}C NMR (75 MHz, $CDCl_3$, 25 °C): δ = 208.7 (=·=), 164.7 (C=O lactam), 156.4 (C=O Boc + O-C^q), 140.7 (=C^q), 130.7 (C^q), 118.7 (2CH PMP), 114.4 (2CH PMP), 114.0 (=CH_2), 94.5 (CH=·=), 81.0 (O-C^q), 80.2 (C-H3), 76.7 (=·=CH_2), 75.0 (OCH_2), 64.0 (C-H4), 55.5 (OCH_3), 45.1 (NCH_2), 44.1 (NCH_2), 28.4 ($3CH_3$ Boc), 19.5 (CH_3); IR ($CHCl_3$): ν = 1955 (=·=), 1755 (C=O), 1694 (C=O) cm^{-1}; HRMS (ES): calcd for $C_{24}H_{32}N_2O_5$ $[M]^+$: 428.2311; found: 428.2328.

2g. From 310 mg (0.80 mmol) of the (prop-2-ynyl)carbamate **1g**, and after chromatography of the residue using hexanes/ethyl acetate (4:1) as eluent gave compound **2g** (191 mg, 45%) as a colorless oil; ^1H NMR (300 MHz, $CDCl_3$, 25 °C): δ = 1.48 (s, 18H, $6CH_3$ Boc), 3.76 (br s, 4H, $2NCH_2$), 4.43 (s, 4H, $2CH_2$-C=·=), 4.76 (dt, 4H, *J* = 6.6, 2.5 Hz, 2=·=CH_2), 5.09 (br s, 2H, 2CH=·=), 7.20 (s, 4H, ArH); ^{13}C NMR (75 MHz, $CDCl_3$, 25 °C): δ = 209.0 (=·=), 155.4 (C=O), 137.2 (C^q), 128.1 (4CH Ph), 86.9 (2CH=·=), 79.9 ($2C^q$ Boc), 76.2 (2=·=CH_2), 49.3 ($2NCH_2$), 44.9 ($2NCH_2$), 28.4 ($6CH_3$ Boc); IR ($CHCl_3$): ν = 1955 (=·=), 1692 (C=O) cm^{-1}; HRMS (ES): calcd for $C_{26}H_{36}N_2O_4$ $[M]^+$: 440.2675; found: 440.2677.

(–)-2h. From 313 mg (1.40 mmol) of the (prop-2-ynyl)carbamate (–)-**1h**, and after chromatography of the residue using hexanes/ethyl acetate (8:1) as eluent gave compound (–)-**2h** (207 mg, 62%) as a colorless oil; $[\alpha]_D$ –34.1 (*c* 1.0, $CHCl_3$); ^1H NMR (300 MHz, $CDCl_2$-$CDCl_2$, 70 °C): δ = 1.45 (s, 9H, $3CH_3$ Boc), 1.47 (s, 3H, CH_3), 1.55 (s, 3H, CH_3), 3.84 (dd, 1H, *J* = 8.8, 2.1

Hz, OC*H*H), 3.99 (dd, 1H, *J* = 8.7, 5.9 Hz, OCH*H*), 4.38 (br s, 1H, NCH), 4.82 (dd, 2H, *J* = 6.6, 2.4 Hz, =·=CH_2), 5.25 (q, 1H, *J* = 6.3 Hz, CH=·=); ^{13}C NMR (75 MHz, $CDCl_2$-$CDCl_2$, 70 °C): δ = 209.4 (=·=), 153.2 (C=O), 95.4 (N-C^q-O), 95.4 (CH=·=), 81.3 (OC^q), 79.2 (=·=CH_2), 69.5 (OCH_2), 57.4 (NCH), 30.1 (3CH_3 Boc), 28.3 (2CH_3); IR ($CHCl_3$): ν = 1958 (=·=), 1698 (C=O) cm^{-1}; HRMS (ES): calcd for $C_{13}H_{21}NO_3$ $[M]^+$: 239.1521; found: 239.1532.

(–)-2i. From 150 mg (0.77 mmol) of the (prop-2-ynyl)carbamate (–)-**1i**, and after chromatography of the residue using hexanes/ethyl acetate (4:1) as eluent gave compound (–)-**2i** (127 mg, 79%) as a colorless oil; $[\alpha]_D$ –64.3 (*c* 0.7, $CHCl_3$); ^{1}H NMR (300 MHz, $CDCl_2$-$CDCl_2$, 70 °C): δ = 1.43 (s, 9H, 3CH_3 Boc), 1.87 (m, 4H, 2CH_2), 3.32 (m, 2H, NCH_2), 4.31 (br s, 1H, NCH), 4.77 (dd, 2H, *J* = 6.7, 2.8 Hz, =·=CH_2), 5.22 (td, 1H, *J* = 6.6, 5.2 Hz, CH=·=); ^{13}C NMR (75 MHz, $CDCl_2$-$CDCl_2$, 70 °C): δ = 208.7 (=·=), 155.7 (C=O Boc), 94.6 (CH=·=), 80.5 (C^q Boc), 75.9 (=·=CH_2), 56.8 (NCH), 47.6 (NCH_2), 32.9 (CH_2), 30.2 (3CH_3 Boc), 24.8 (CH_2); IR ($CHCl_3$): ν = 1957 (=·=), 1696 (C=O) cm^{-1}; HRMS (ES): calcd for $C_{12}H_{19}NO_2$ $[M]^+$: 209.1416; found: 209.1409.

Preparation of 2j. From 224 mg (0.70 mmol) of the (prop-2-ynyl)carbamate **1j**, and after chromatography of the residue using hexanes/ethyl acetate (8:1) as eluent gave compound **2j** (183 mg, 77%) as a colorless oil. After the standard treatment for 1 h, a 1:1 mixture of the intermediate alkyne *tert*-butyl 3-bromo-2-[3-(diisopropylamino)prop-1-ynyl]-1*H*-indole-1-carboxylate and allene **2j** were obtained. The alkyne was resubmitted to the standard Crabbé conditions for an additional hour in order to get the optimal yield. *tert-Butyl 3-bromo-2-[3-(diisopropylamino)prop-1-ynyl]-1H-indole-1-carboxylate:* ^{1}H NMR (300 MHz, $CDCl_3$, 25 °C): δ = 1.20 (d, 12H, *J* = 6.5 Hz, 4CH_3), 1.69 (s, 9H, 3CH_3 Boc), 3.37 (m, 2H, *J* = 6.6 Hz, 2CH *i*Pr), 3.81 (s, 2H, CH_2-N), 7.34 (m, 2H, indole), 7.50 (m, 1H, indole), 8.13 (d, 1H, *J* = 8.3 Hz, indole); ^{13}C NMR (75 MHz, $CDCl_3$, 25 °C): δ = 148.8 (C=O), 134.8 (C^q), 128.1 (C^q), 126.3 (CH indole), 123.5 (CH indole), 120.2 (C^q), 119.4 (CH indole), 115.5 (CH indole), 107.1 (C^q-Br), 99.7 (O-C^q), 84.5 (*C*≡C), 74.9 (C≡*C*), 48.5 (2NCH), 35.2 (NCH_2), 28.1 (3CH_3 Boc), 20.8 (2CH_3). *tert-Butyl 3-bromo-2-(propa-1,2-dienyl)-1H-indole-1-carboxylate **2j**:* ^{1}H NMR (300 MHz, $CDCl_3$, 25 °C): δ = 5.18 (d, 2H, *J* = 7.1 Hz, =·=CH_2), 6.74 (t, 1H, *J* = 7.0 Hz, CH=·=), 7.31 (m, 2H, Ar), 7.52 (m, 1H, Ar), 8.07 (m, 1H, Ar); ^{13}C NMR (75 MHz, $CDCl_3$, 25 °C): δ = 210.7 (=·=), 151.4 (C=O), 136.6 (C^q), 135.3 (C^q), 128.9 (C^q), 125.2 (CH indole), 123.4 (CH indole), 119.2 (CH indole), 115.44 (CH indole), 105.1 (C^q), 85.4 (CH=·=), 84.9

(C^q Boc), 78.9 (=·=CH_2), 28.2 (3CH_3 Boc); IR ($CHCl_3$): ν = 1954 (=·=), 1690 (C=O) cm^{-1}; HRMS (ES): calcd for $C_{16}H_{16}BrNO_2$ $[M]^+$: 333.0364; found: 333.0365.

General procedure for the gold-catalyzed heterocyclisation reaction of allenic carbamates 2 to 1,3-oxazinan-2-ones 3. [$AuClPPh_3$] (0.025 mmol), AgOTf (0.025 mmol), and *p*-toluenesulfonic acid (0.1 mmol) were sequentially added to a stirred solution of the corresponding allenic carbamate **2** (1.0 mmol) in dichloromethane (10 mL). The resulting mixture was stirred at RT until disappearance of the starting material (TLC, 2–8 h). Next, the reaction was filtered through a pack of celite. The filtrate was extracted with dichloromethane (3 × 25 mL), and the combined extracts were washed twice with brine. The organic layer was dried ($MgSO_4$) and concentrated under reduced pressure. Chromatography of the residue gave analytically pure adducts **3**. Spectroscopic and analytical data for pure forms of **3** follow.

3a. From 50 mg (0.19 mmol) of the allenic carbamate **2a**, and after chromatography of the residue using hexanes/ethyl acetate (4:1) as eluent gave compound **3a** (30 mg, 77%) as a colorless oil; ^1H NMR (300 MHz, $CDCl_3$, 25 °C): δ = 2.55 (t, 2H, J = 6.2 Hz, CH_2-CH=), 3.22 (t, 2H, J = 6.2 Hz, NCH_2), 4.24 (dt, 1H, J = 1.8, 1.0 Hz, NC*H*H-Ph), 4.59 (s, 2H, =CH_2), 4.67 (d, 1H, J = 1.6 Hz, NCH*H*-Ph), 7.32 (m, 5H, Ph); ^{13}C NMR (75 MHz, $CDCl_3$, 25 °C): δ = 152.7 (C=O), 151.1 (C^q), 136.1 (C^q), 128.8 (2CH Ph), 128.0 (2CH Ph), 127.9 (CH Ph), 92.8 (=CH_2), 52.7 (NCH_2-Ph), 43.1 (NCH_2), 26.2 (CH_2); IR ($CHCl_3$): ν = 1694 (C=O) cm^{-1}; HRMS (ES): calcd for $C_{12}H_{13}NO_2$ $[M]^+$: 203.0946; found: 203.0952.

3b. From 50 mg (0.17 mmol) of the allenic carbamate **2b**, and after chromatography of the residue using hexanes/ethyl acetate (4:1) as eluent gave compound **3a** (37 mg, 94%) as a pale brown solid; mp 48–49 °C; ^1H NMR (300 MHz, $CDCl_3$, 25 °C): δ = 2.53 (t, 2H, J = 6.2 Hz, CH_2-CH=), 3.20 (t, 2H, J = 6.2 Hz, NCH_2), 3.81 (s, 3H, OMe), 4.23 (dt, 1H, J = 1.5, 1.0 Hz, NC*H*H-Ar), 4.53 (s, 2H, =CH_2), 4.66 (d, 1H, J = 1.5 Hz, NCH*H*-Ar), 6.88 (d, 2H, J = 8.7 Hz, ArH), 7.24 (d, 2H, J = 8.7 Hz, ArH); ^{13}C NMR (75 MHz, $CDCl_3$, 25 °C): δ = 159.4 (C^q), 152.7 (C=O), 129.6 (2 CH Ar), 129.5 (C^q), 128.2 (C^q), 114.1 (2CH Ar), 92.7 (=CH_2), 55.3 (OCH_3), 52.1 (NCH_2-Ar), 42.8 (NCH_2), 26.2 (CH_2); IR ($CHCl_3$): ν = 1690 (C=O) cm^{-1}; HRMS (ES): calcd for $C_{13}H_{15}NO_3$ $[M]^+$: 233.1052; found: 233.1048.

(+)-3d. From 45 mg (0.12 mmol) of the allenic carbamate (+)-**2d**, and after chromatography of the residue using hexanes/ethyl acetate (4:1) as eluent gave compound (+)-**3d** (25 mg, 64%) as a pale brown solid; mp 89–90 °C; $[\alpha]_D$ +15.6 (*c* 1.2, $CHCl_3$); ^1H NMR (300 MHz, $CDCl_3$, 25 °C): δ = 2.43 (m, 2H, CH_2-CH=), 3.16 (m, 1H, NC*H*H-CH_2), 3.44 (m, 1H, NCH*H*-CH_2), 3.48 (dd, 1H, *J* = 14.2, 6.7 Hz, NC*H*H), 3.66 (s, 3H, OCH_3), 3.78 (s, 3H, OCH_3), 3.91 (dd, 1H, *J* = 14.3, 4.3 Hz, NCH*H*), 4.24 (s, 1H, H4), 4.62 (m, 2H, =CH_2), 4.66 (m, 1H, H3), 6.89 (d, 2H, *J* = 9.1 Hz, ArH), 7.49 (d, 2H, *J* = 9.0 Hz, ArH); ^{13}C NMR (75 MHz, $CDCl_3$, 25 °C): δ = 164.2 (C=O lactam), 156.6 (C^q), 152.5 (C=O), 151.2 (C^q), 130.1 (C^q), 118.8 (2CH Ar), 114.6 (2CH Ar), 93.1 (=CH_2), 82.4 (C-H3), 59.2 (OMe), 55.5 (OMe Ar), 54.9 (C-H4), 48.1 (NCH_2), 46.2 (NCH_2), 26.1 (CH_2); IR ($CHCl_3$): ν = 1754 (C=O lactam), 1690 (C=O oxazinanone) cm^{-1}; HRMS (ES): calcd for $C_{17}H_{20}N_2O_5$ $[M]^+$: 332.1372; found: 332.1381.

(+)-3e. From 100 mg (0.27 mmol) of the allenic carbamate (+)-**2e**, and after chromatography of the residue using hexanes/ethyl acetate (2:1) as eluent gave compound (+)-**3e** (60 mg, 70%) as a pale brown solid; mp 83–84 °C; $[\alpha]_D$ +49.8 (*c* 2.6, in $CHCl_3$); ^1H NMR (300 MHz, $CDCl_3$, 25 °C): δ = 2.43 (m, 2H, CH_2), 3.16 (m, 3H, NCH_2 + NC*H*H), 3.59 (s, 3H, OMe), 3.82 (m, 1H, NCH*H*), 3.91 (dt, 1H, *J* = 6.2, 5.0 Hz, H4), 4.23 (d, 1H, *J* = 1.5 Hz, =C*H*H), 4.24 (d, 1H, *J* = 15.0 Hz, C*H*H-Ph), 4.49 (d, 1H, *J* = 5.0 Hz, H3), 4.59 (d, 1H, *J* = 15.0 Hz, CH*H*-Ph), 4.64 (d, 1H, *J* = 1.3 Hz, =CH*H*), 7.30 (m, 5H, Ph); ^{13}C NMR (75 MHz, $CDCl_3$, 25 °C): δ = 167.3 (C=O lactam), 152.5 (C=O), 150.7 (=C^q), 135.3 (C^q Ph), 128.9 (2CH Ph), 128.4 (2CH Ph), 127.8 (CH Ph), 92.9 (=CH_2), 83.2 (C-H3), 59.0 (C-H4), 55.2 (OMe), 48.3 (CH_2-Ph), 45.3 (NCH_2). 44.5 (*C*H_2-C=), 26.1 (CH_2); IR ($CHCl_3$): ν = 1752 (C=O lactam), 1691 (C=O oxazinanone) cm^{-1}; HRMS (ES): calcd for $C_{17}H_{20}N_2O_4$ $[M]^+$: 316.1423; found: 316.1421.

(+)-3f. From 60 mg (0.14 mmol) of the allenic carbamate (+)-**2f**, and after chromatography of the residue using hexanes/ethyl acetate (5:1) as eluent gave compound (+)-**3f** (35 mg, 67%) as a brown solid; mp 96–97 °C; $[\alpha]_D$ +39.5 (*c* 1.5, $CHCl_3$); ^1H NMR (300 MHz, $CDCl_3$, 25 °C): δ = 1.78 (s, 3H, CH_3), 2.41 (m, 2H, CH_2-C=), 3.16 (ddd, 1H, *J* = 11.8, 6.8, 5.3 Hz, NC*H*H-CH_2), 3.46 (ddd, 1H, *J* = 12.8, 7.7, 5.3 Hz, NCH*H*-CH_2), 3.55 (dd, 1H, *J* = 14.3, 7.2 Hz, C*H*HN), 3.79 (s, 3H, OCH_3), 3.91 (dd, 1H, *J* = 14.2, 4.8 Hz, CH*H*N), 4.24 (d, 1H, *J* = 1.4 Hz, =C*H*H oxazinan-2-one), 4.25 (m, 2H, OCH_2), 4.67 (d, 1H, *J* = 1.3 Hz, =CH*H* oxazinan-2-one), 4.68 (m, 1H, H4), 4.76 (d,

1H, J = 5.2 Hz, H3), 4.98 (s, 1H, =C*H*H), 5.04 (s, 1H, =CH*H*), 6.89 (d, 2H, J = 9.0 Hz, ArH), 7.50 (d, 2H, J = 9.0 Hz, ArH); ^{13}C NMR (75 MHz, $CDCl_3$, 25 °C): δ = 164.4 (C=O lactam), 156.6 (O-C^q Ar), 152.4 (C=O), 151.2 (=C^q oxazinan-2-one), 140.7 (=C^q), 130.2 (C^q Ar), 118.8 (2CH Ar), 114.6 (2CH Ar), 113.4 (=CH_2), 93.2 (=CH_2 oxazinan-2-one), 80.1 (C-H3), 75.0 (OCH_2), 55.5 (OMe Ar), 54.9 (C-H4), 48.4 (NCH_2), 46.3 (NCH_2), 26.1 (CH_2), 19.5 (CH_3); IR ($CHCl_3$): ν = 1752 (C=O lactam), 1688 (C=O oxazinanone) cm^{-1}; HRMS (ES): calcd for $C_{20}H_{24}N_2O_5$ $[M]^+$: 372.1685; found: 372.1670.

3g. From 75 mg (0.18 mmol) of the bis(allenic carbamate) **2g**, and after chromatography of the residue using hexanes/ethyl acetate (4:1) as eluent gave compound **3g** (30 mg, 50%) as a brown solid; mp 93–94 °C; ^{1}H NMR (300 MHz, $CDCl_3$, 25 °C): δ = 2.57 (t, 4H, J = 6.2 Hz, C*H₂*-CH=), 3.24 (td, 4H, J = 6.3, 1.8 Hz, CH_2N), 4.26 (m, 4H, NC*H*H-Ar), 4.58 (s, 4H, =CH_2), 4.68 (s, 2H, NCH*H*-Ar), 7.28 (s, 4H, ArH); ^{13}C NMR (75 MHz, $CDCl_3$, 25 °C): δ = 152.6 (2C=O), 151.1 (2C^q), 135.8 (2C^{4q}), 128.4 (4CH Ar), 93.0 (2=CH_2), 52.4 (2NCH_2-Ar), 43.3 (2 CH_2N), 26.1 (2CH_2); IR ($CHCl_3$): ν = 1687 (C=O) cm^{-1}; HRMS (ES): calcd for $C_{18}H_{20}N_2O_4$ $[M]^+$: 328.1423; found: 328.1439.

(–)-3h. From 90 mg (0.38 mmol) of the allenic carbamate (–)-**2h**, and after chromatography of the residue using hexanes/ethyl acetate (4:1) as eluent gave compound (–)-**3h** (36 mg, 52%) as a colorless oil; $[\alpha]_D$ –16.4 (*c* 0.6, $CHCl_3$); ^{1}H NMR (300 MHz, $CDCl_3$, 25 °C): δ = 1.61 (s, 3H, Me), 1.65 (s, 3H, Me), 2.23 (ddt, 1H, J = 13.8, 11.9, 1.9 Hz, NCH-C*H*H), 2.75 (dd, 1H, J = 13.8, 3.4 Hz, NCH-CH*H*), 3.57 (dd, 1H, J = 9.5, 8.8 Hz, OC*H*H), 3.77 (m, 1H, NCH), 4.24 (dd, 1H, J = 8.7, 5.7 Hz, OCH*H*), 4.28 (t, 1H, J = 1.8 Hz, =C*H*H), 4.72 (t, 1H, J = 1.4 Hz, =CH*H*); ^{13}C NMR (75 MHz, $CDCl_3$, 25 °C): δ = 151.7 (C=O), 146.7 (=C^q), 95.2 (C^q), 93.5 (=CH_2), 69.3 (OCH_2), 53.6 (NCH), 30.1 (CH_2), 25.7 (CH_3), 23.7 (CH_3); IR ($CHCl_3$): ν = 1712 (C=O), 1216 (C-O) cm^{-1}; HRMS (ES): calcd for $C_9H_{13}NO_3$ $[M]^+$: 183.0895; found: 183.0902.

(–)-3i. From 50 mg (0.24 mmol) of the allenic carbamate (–)-**2i**, and after chromatography of the residue using hexanes/ethyl acetate (3:1) as eluent gave compound (–)-**3i** (30 mg, 82%) as a pale brown solid; mp 134–135 °C; $[\alpha]_D$ –22.9 (*c* 1.4 in $CHCl_3$); ^{1}H NMR (300 MHz, $CDCl_3$, 25 °C): δ = 1.52 (m, 1H, C*H*H), 1.84 (m, 1H, CH*H*), 2.02 (m, 1H, C*H*H), 2.19 (m, 2H, CH*H* + =C-

C*H*H), 2.78 (dd, 1H, J = 13.9, 3.6 Hz, =C-CH*H*), 3.49 (1H, CHN), 3.55 (dd, 2H, J = 9.7, 5.1 Hz, NCH_2), 4.24 (t, 1H, J = 1.7 Hz, =C*H*H), 4.66 (t, 1H, J = 1.7 Hz, =CH*H*); ^{13}C NMR (75 MHz, $CDCl_3$, 25 °C): δ = 152.5 (C=O), 150.0 (=C^q), 92.8 (=CH_2), 55.2 (CHN), 46.4(NCH_2), 33.2 (=C-*C*H_2), 32.8 (CHN-*C*H_2), 22.9 (NCH_2-*C*H_2); IR ($CHCl_3$): ν = 1705 (C=O) cm^{-1}; HRMS (ES): calcd for $C_8H_{11}NO_2$ $[M]^+$: 153.0790; found: 153.0788.

3j. From 70 mg (0.21 mmol) of the allenic carbamate **2j**, and after chromatography of the residue using hexanes/ethyl acetate (6:1) as eluent gave an inseparable mixture (30:70) of compounds **3j** and **4j** (34 mg, 58%) as a yellow solid; mp 133–134 °C; ^1H NMR (300 MHz, $CDCl_3$, 25 °C): δ = 2.30 (d, 3H, J = 1.0 Hz, Me M), 3.86 (t, 2H, J = 1.2 Hz, CH_2 m), 4.73 (dt, 1H, J = 2.5, 1.4 Hz, =C*H*H m), 5.05 (dt, 1H, J = 2.2, 1.0 Hz, =CH*H* m), 6.35 (q, 1H, J = 1.1 Hz, =CH M), 7.48 (m, 6H, 3 CH indole M + 3 CH indole m), 8.27 (m, 1H, CH indole m), 8.40 (m, 1H, CH indole M); ^{13}C NMR (75 MHz, $CDCl_3$, 25 °C): δ = 152.3 (=C^q M), 152.2 (=C^q m), 143.2 (C=O m), 143.2 (C=O M), 132.5 (C^q m), 132.1 (C^q M), 131.0 (C^q m), 130.3 (C^q M), 130.2 (C^q m), 129.7 (C^q M), 125.8 (CH indole m), 125.2 (CH indole M), 125.2 (CH indole M), 124.8 (CH indole m), 119.0 (CH indole m), 118.8 (CH indole M), 115.5 (CH indole M), 115.2 (CH indole m), 98.6 (=CH_2 m), 95.7 (=CH M), 93.2 (C^q m), 90.2 (C^q M), 25.8 (CH_2 m), 19.0 (CH_3 M); IR ($CHCl_3$): ν = 1693 (C=O), 751 (C-Br) cm^{-1}; HRMS (ES): calcd for $C_{12}H_8BrNO_2$ $[M]^+$: 276.9738; found: 276.9737.

General procedure for the gold-catalyzed heterocyclisation reaction of allenic carbamates 2 to 1,3-oxazin-2-ones 4. [$AuClPPh_3$] (0.025 mmol), AgOTf (0.025 mmol), and *p*-toluenesulfonic acid (0.1 mmol) were sequentially added to a stirred solution of the corresponding allenic carbamate **2** (1.0 mmol) in dichloromethane (10 mL). The resulting mixture was heated in a sealed tube at 130 °C until disappearance of the starting material (TLC, 0.5–6 h). The reaction was allowed to cool to room temperature and filtered through a pack of celite. The filtrate was extracted with dichloromethane (3 × 25 mL), and the combined extracts were washed twice with brine. The organic layer was dried ($MgSO_4$) and concentrated under reduced pressure. Chromatography of the residue gave analytically pure adducts **4**. Spectroscopic and analytical data for pure forms of **4** follow.

4a. From 50 mg (0.19 mmol) of the allenic carbamate **2a**, and after chromatography of the residue using hexanes/ethyl acetate (4:1) as eluent gave compound **4a** (27 mg, 70%) as a colorless oil; ^{1}H NMR (300 MHz, $CDCl_3$, 25 °C): δ = 1.86 (td, 3H, *J* = 1.9, 1.2 Hz, CH_3), 3.68 (dq, 2H, *J* = 3.2, 1.9 Hz, NCH_2), 4.58 (s, 2H, Ph-CH_2-N), 4.76 (m, 1H, =CH), 7.33 (m, 2H, ArH); ^{13}C NMR (75 MHz, $CDCl_3$, 25 °C): δ = 150.9 (C=O), 148.1 (C^q), 135.6 (C^q), 128.7 (2CH Ar), 128.2 (2CH Ar), 127.9 (CH Ar), 94.4 (=CH), 52.1 (CH_2), 44.6 (CH_2), 18.3 (CH_3); IR ($CHCl_3$): ν = 1685 (C=O) cm^{-1}; HRMS (ES): calcd for $C_{12}H_{13}NO_2$ $[M]^+$: 203.0946; found: 203.0952.

4b. From 50 mg (0.17 mmol) of the allenic carbamate **2b**, and after chromatography of the residue using hexanes/ethyl acetate (4:1) as eluent gave compound **4b** (28 mg, 70%) as a pale brown solid; mp 105–106 °C; ^{1}H NMR (300 MHz, $CDCl_3$, 25 °C): δ = 1.85 (td, 3H, *J* = 1.7, 1.3 Hz, CH_3), 3.66 (dq, 2H, *J* = 3.8, 1.9 Hz, NCH_2), 3.81 (s, 3H, OCH_3), 4.52 (s, 2H, Ar-CH_2-N), 4.75 (m, 1H, =CH), 6.88 (d, 2H, *J* = 8.8 Hz, ArH), 7.26 (d, 2H, *J* = 8.8 Hz, ArH); ^{13}C NMR (75 MHz, $CDCl_3$, 25 °C): δ = 159.4 (C^q), 150.9 (C=O), 148.0 (C^q), 129.7 (2CH Ar), 127.7 (C^q), 114.1 (2CH Ar), 94.4 (=CH), 55.3 (OMe), 51.5 (CH_2), 44.4 (CH_2), 18.3 (CH_3); IR ($CHCl_3$): ν = 1681 (C=O) cm^{-1}; HRMS (ES): calcd for $C_{13}H_{15}NO_3$ $[M]^+$: 233.1052; found: 233.1052.

(+)-4c. From 45 mg (0.16 mmol) of the allenic carbamate (+)-**2c**, and after chromatography of the residue using hexanes/ethyl acetate (5:1) as eluent gave compound (+)-**4c** (26 mg, 70%) as a colorless oil; $[\alpha]_D$ +23.4 (*c* 0.8, $CHCl_3$); ^{1}H NMR (300 MHz, $CDCl_3$, 25 °C): δ = 1.35 (s, 3H, Me), 1.43 (s, 3H, Me), 1.85 (m, 3H, Me), 3.17 (dd, 1H, *J* = 14.3, 7.2 Hz, NC*H*H), 3.67 (dd, 1H, *J* = 8.5, 6.6 Hz, NCH*H*), 3.78 (dd, 1H, *J* = 14.3, 3.3 Hz, OC*H*H), 3.93 (dm, 1H, *J* = 14.8 1.9 Hz, OCH*H*), 4.10 (m, 2H, CH_2N), (qd, 1H, *J* = 6.7, 3.2 Hz, OCH), 4.80 (m, 1H, =CH); ^{13}C NMR (75 MHz, $CDCl_3$, 25 °C): δ = 150.9 (C^q), 148.0 (C=O), 109.5 (C^q acetonide), 94.9 (=CH), 74.7 (OCH), 67.1 (OCH_2), 51.6 (NCH_2), 47.4 (NCH_2). 26.8 (CH_3 acetonide), 25.4 (CH_3 acetonide), 18.3 (CH_3); IR ($CHCl_3$): ν = 1689 (C=O) cm^{-1}; HRMS (ES): calcd for $C_{11}H_{17}NO_4$ $[M]^+$: 227.1158; found: 227.1162.

(+)-4d. From 45 mg (0.12 mmol) of the allenic carbamate (+)-**2d**, and after chromatography of the residue using hexanes/ethyl acetate (4:1) as eluent gave compound (+)-**4d** (22 mg, 57%) as a pale brown oil; $[\alpha]_D$ +5.6 (*c* 0.5 in $CHCl_3$); ^{1}H NMR (300 MHz, $CDCl_3$, 25 °C): δ = 1.85 (d, 3H, *J*

= 1.1 Hz, CH_3), 3.38 (dd, 1H, *J* = 14.1, 7.0 Hz, NCH*H*), 3.67 (m, 1H, NC*H*H), 3.67 (s, 3H, OCH_3), 3.79 (s, 3H, Ar-OCH_3), 3.96 (dd, 2H, *J* = 14.2, 3.7 Hz, CH_2N), 4.62 (s, 1H, H3), 4.64 (m, 1H, H4), 4.72 (m, 1H, =CH), 6.90 (d, 2H, *J* = 9.0 Hz, ArH), 7.54 (d, 2H, *J* = 9.0 Hz, ArH); ^{13}C NMR (75 MHz, $CDCl_3$, 25 °C): δ = 164.1 (C=O lactam), 156.6 (C^q), 156.6 (C=O), 148.0 (C^q), 130.2 (C^q), 118.8 (2CH Ar), 114.6 (2CH Ar), 94.9 (=CH), 82.4 (C-H3), 59.2 (OMe), 55.5 (OMe Ar), 54.9 (C-H4), 47.3 (CH_2), 46.8 (CH_2). 18.3 (CH_3); IR ($CHCl_3$): ν = 1752 (C=O lactam), 1692 (C=O oxazinone) cm^{-1}; HRMS (ES): calcd for $C_{17}H_{20}N_2O_5$ $[M]^+$: 332.1372; found: 332.1379.

(+)-4e. From 45 mg (0.12 mmol) of the allenic carbamate (+)-**2e**, and after chromatography of the residue using hexanes/ethyl acetate (4:1) as eluent gave compound (+)-**4e** (26 mg, 68%) as a pale brown oil; $[\alpha]_D$ +20.3 (*c* 0.7, $CHCl_3$); ^1H NMR (300 MHz, $CDCl_3$, 25 °C): δ = 1.85 (m, 3H, CH_3), 3.20 (dd, 1H, *J* = 14.3, 6.3 Hz, NCH*H*), 3.59 (s, 3H, OCH_3), 3.63 (m, 1H, NC*H*H), 3.74 (m, 2H, CH_2N), 3.97 (q, 1H, *J* = 5.5 Hz, H4), 4.23 (d, 1H, *J* = 14.9 Hz, C*H*H-Ph), 4.48 (d, 1H, *J* = 5.5 Hz, H3), 4.61 (d, 1H, *J* = 14.9 Hz, CH*H*-Ph), 4.72 (m, 1H, =CH), 7.37 (m, 5H, ArH); ^{13}C NMR (75 MHz, $CDCl_3$, 25 °C): δ = 167.4 (C=O lactam), 150.8 (C=O), 148.1 (C^q), 135.5 (C^q), 128.9 (2CH Ar), 128.5 (2CH Ar), 127.9 (CH Ar), 94.8 (=CH), 83.4 (C-H3), 59.0 (OMe), 55.1 (C-H4), 47.6 (NCH_2), 46.8 (CH_2N), 44.8 (CH_2-Ph), 18.3 (CH_3); IR ($CHCl_3$): ν = 1752 (C=O lactam), 1690 (C=O oxazinone) cm^{-1}; HRMS (ES): calcd for $C_{17}H_{20}N_2O_4$ $[M]^+$: 316.1423; found: 316.1436.

(+)-4f. From 34 mg (0.08 mmol) of the allenic carbamate (+)-**2f**, and after chromatography of the residue using hexanes/ethyl acetate (4:1) as eluent gave compound (+)-**4f** (20 mg, 66%) as a brown oil; $[\alpha]_D$ +41.2 (*c* 0.8, $CHCl_3$); ^1H NMR (300 MHz, $CDCl_3$, 25 °C): δ = 1.78 (s, 3H, =C-CH_3), 1.85 (d, 3H, *J* = 1.1 Hz, CH_3), 3.44 (dd, 1H, *J* = 14.2, 7.6 Hz, β-lact-NCH*H*), 3.68 (dm, 1H, *J* = 14.8 Hz, NC*H*H-CH=), 3.79 (s, 3H, OCH_3), 3.97 (dd, 2H, *J* = 14.1, 4.2 Hz, β-lact-NC*H*H), 4.00 (dm, 1H, *J* = 13.3 Hz, NCH*H*-CH=), 4.26 (m, 2H, OCH_2), 4.66 (m, 1H, H4), 4.71 (m, 1H, =CH), 4.75 (d, 1H, *J* = 5.2 Hz, H3), 4.97 (s, 1H, =C*H*H), 5.04 (s, 1H, =CHH), 6.90 (d. 2H, *J* = 9.0 Hz, ArH), 7.54 (d, 2H, *J* = 9.0 Hz, ArH); ^{13}C NMR (75 MHz, $CDCl_3$, 25 °C): δ = 164.4 (C=O lactam), 156.5 (O-C^q), 151.2 (C=O), 148.0 (=C^q), 140.8 (=C^q), 130.2 (C^q), 118.7 (2CH Ar), 114.5 (2CH Ar), 113.3 (=CH_2), 94.9 (=CH), 80.0 (C-H3), 74.9 (OCH_2), 55.5 (OMe Ar), 54.9 (C-H4), 47.3 (β-lact-CH_2), 46.9 (NCH_2). 19.5 (CH_3), 18.3 (CH_3); IR ($CHCl_3$): ν = 1755 (C=O lactam), 1686 (C=O oxazinone) cm^{-1}; HRMS (ES): calcd for $C_{20}H_{24}N_2O_5$ $[M]^+$: 372.1685; found: 372.1704.

4g. From 75 mg (0.18 mmol) of the bis(allenic carbamate) **2g**, and after chromatography of the residue using hexanes/ethyl acetate (4:1) as eluent gave compound **4g** (31 mg, 54%) as a brown solid; mp 144–145 °C; ^{1}H NMR (300 MHz, $CDCl_3$, 25 °C): δ = 1.86 (td, 6H, *J* = 1.8, 1.2 Hz, CH_3), 3.69 (dq, 4H, *J* = 3.3, 2.0 Hz, CH_2N), 4.56 (s, 4H, Ph-CH_2-N), 4.77 (m, 2H, =CH), 7.31 (s, 4H, ArH); ^{13}C NMR (75 MHz, $CDCl_3$, 25 °C): δ = 150.9 (2C=O), 148.1 (2C^q), 135.4 (2C^q), 128.5 (4CH Ar), 94.4 (2=CH), 51.8 (CH_2), 44.7 (CH_2). 18.4 (CH_3); IR ($CHCl_3$): ν = 1685 (C=O) cm^{-1}; HRMS (ES): calcd for $C_{18}H_{20}N_2O_4$ $[M]^+$: 328.1423; found: 328.1425.

(–)-4i. From 50 mg (0.24 mmol) of the allenic carbamate (–)-**2i**, and after chromatography of the residue using hexanes/ethyl acetate (4:1) as eluent gave compound (–)-**4i** (25 mg, 67%) as a yellow oil; $[\alpha]_D$ –35.3 (*c* 0.8, $CHCl_3$); ^{1}H NMR (300 MHz, $CDCl_3$, 25 °C): δ = 1.55 (qd, 1H, *J* = 11.4, 7.7 Hz, NCH_2-C*H*H), 1.86 (m, 1H, NCH-C*H*H), 1.87 (dd, 3H, *J* = 1.9, 1.2 Hz, CH_3), 2.02 (m, 2H, NCH_2-CH*H* + NCH-CH*H*), 3.45 (ddd, 1H, J = 11.5, 9.8, 2.9 Hz, NC*H*H), 3.71 (dt, 1H, *J* = 11.5, 8.7 Hz, NCH*H*), 4.02 (m, 1H, NCH), 4.93 (t, 1H, *J* = 1.3 Hz, =CH); ^{13}C NMR (75 MHz, $CDCl_3$, 25 °C): δ = 150.2 (C=O), 148.2 (=C^q), 98.64 (=CH), 56.0 (NCH), 45.6 (NCH_2), 33.3 (NCH_2-*C*H_2), 21.9 (NCH-*C*H_2), 18.3 (CH_3); IR ($CHCl_3$): ν = 1687 (C=O) cm^{-1}; HRMS (ES): calcd for $C_8H_{11}NO_2$ $[M]^+$: 153.0790; found: 153.0786.

4j. From 50 mg (0.15 mmol) of the allenic carbamate **2j**, and after chromatography of the residue using hexanes/ethyl acetate (5:1) as eluent gave compound **4j** (28 mg, 66%) as a yellow solid; mp 141–142 °C; ^{1}H NMR (300 MHz, $CDCl_3$, 25 °C): δ = 2.30 (d, 3H, *J* = 1.0 Hz, Me), 6.35 (q, 1H, *J* = 1.1 Hz, =CH), 7.45 (m, 2H, CH indole), 7.57 (m, 1H, CH indole), 8.40 (m, 1H, CH indole); ^{13}C NMR (75 MHz, $CDCl_3$, 25 °C): δ = 152.3 (=C^q), 143.2 (C=O), 132.1 (C^q), 130.3 (C^q), 129.7 (C^q), 125.2 (CH indole), 125.2 (CH indole), 118.8 (CH indole), 115.5 (CH indole), 95.7 (=CH), 90.2 (C^q), 19.0 (CH_3); IR ($CHCl_3$): ν = 1723 (C=O), 751 (C-Br) cm^{-1}; HRMS (ES): calcd for $C_{12}H_8BrNO_2$ $[M]^+$: 276.9738; found: 276.9756.

Computational details

All the calculations reported in this paper were obtained with the GAUSSIAN 09 suite of programs [1]. Electron correlation was partially taken into account using the hybrid functional usually denoted as B3LYP [2] using the double-ζ quality plus polarization def2-SVP basis set [3] for all atoms. Reactants and products were characterized by frequency calculations [4], and have positive definite Hessian matrices. Transition structures (TS's) show only one negative eigenvalue in their diagonalized force constant matrices, and their associated eigenvectors were confirmed to correspond to the motion along the reaction coordinate under consideration using the Intrinsic Reaction Coordinate (IRC) method [5]. Solvents effects were taken into account using the Polarizable Continuum Model (PCM) [6]. Single point calculations (PCM-M06/def2-SVP) on the gas-phase-optimized geometries were performed to estimate the change in the Gibbs energies in the presence of CH_2Cl_2 as solvent using the dispersion corrected M06 [7] functional. This level is denoted PCM-M06/def2-SVP//B3LYP/def2-SVP.

Cartesian coordinates (in Å) and free energies (in a. u., solvent corrected) of all the stationary points discussed in the text. All calculations have been performed at the PCM-M06/def2-SVP//B3LYP/def2-SVP level of theory.

1M: E= -595.410765

C	-2.114137000	0.899295000	0.771896000
C	-3.058842000	0.470394000	-0.338210000
H	-2.060189000	0.115504000	1.536074000
H	-2.500916000	1.820289000	1.243898000
H	-3.228837000	1.182923000	-1.157143000
N	-0.756255000	1.171207000	0.311004000
C	-0.518044000	2.390371000	-0.441729000
H	0.545166000	2.652287000	-0.411640000
H	-0.818837000	2.303466000	-1.502718000
H	-1.096226000	3.212754000	0.009815000
O	-0.040908000	-0.891449000	0.994844000
O	1.334839000	0.499861000	-0.174408000
C	2.500961000	-0.386084000	-0.148855000
C	3.553850000	0.414873000	-0.918135000
H	3.756015000	1.373473000	-0.416238000
H	4.495601000	-0.151563000	-0.978033000
H	3.208534000	0.626600000	-1.941532000
C	2.952223000	-0.623956000	1.296315000
H	3.137263000	0.336662000	1.802663000
H	2.192639000	-1.180695000	1.859135000
H	3.891959000	-1.198355000	1.302705000
C	2.181221000	-1.696562000	-0.876279000
H	1.413494000	-2.267162000	-0.339146000
H	1.820965000	-1.489091000	-1.896263000
H	3.092598000	-2.310021000	-0.954325000
C	0.170475000	0.160399000	0.421616000
C	-3.674767000	-0.686899000	-0.376734000
C	-4.291451000	-1.841949000	-0.402352000
H	-3.836056000	-2.719109000	-0.875806000
H	-5.279773000	-1.973008000	0.052854000

INT1-A: E= -1191.763908

C	1.095031000	0.998654000	0.523568000
C	0.417613000	0.103825000	-0.524428000
H	1.078900000	0.487270000	1.496976000
H	0.554841000	1.951323000	0.617761000
H	0.413445000	0.487690000	-1.555236000
N	2.454250000	1.294965000	0.118107000
C	2.771037000	2.638912000	-0.348245000
H	3.822189000	2.679835000	-0.648072000
H	2.149953000	2.912697000	-1.218421000
H	2.602927000	3.385712000	0.447169000
O	3.040967000	-0.843517000	0.618245000
O	4.610737000	0.665624000	-0.056563000
C	5.783469000	-0.236628000	0.022468000
C	6.933276000	0.670820000	-0.412932000
H	7.025098000	1.535034000	0.261860000
H	7.881390000	0.113438000	-0.391926000
H	6.773013000	1.042374000	-1.436308000
C	5.971369000	-0.707055000	1.466119000
H	6.052991000	0.155813000	2.145207000
H	5.137156000	-1.340816000	1.792675000
H	6.903641000	-1.287115000	1.543382000
C	5.603473000	-1.400188000	-0.954649000
H	4.772191000	-2.050181000	-0.653593000

H	5.416274000	-1.024400000	-1.972744000
H	6.524838000	-2.001803000	-0.981322000
C	3.377470000	0.270195000	0.253548000
Au	-1.852422000	-0.138744000	-0.149031000
P	-4.159539000	0.184824000	0.167885000
C	-4.701079000	-0.172179000	1.879544000
H	-4.167224000	0.482517000	2.583903000
H	-5.785016000	-0.004072000	1.977951000
H	-4.473307000	-1.218303000	2.131520000
C	-4.677609000	1.906897000	-0.177403000
H	-4.432920000	2.168034000	-1.217443000
H	-5.762972000	2.013077000	-0.023658000
H	-4.148105000	2.598719000	0.493940000
C	-5.186658000	-0.872810000	-0.917407000
H	-4.975969000	-1.933051000	-0.714463000
H	-6.254808000	-0.674800000	-0.735878000
H	-4.953239000	-0.663619000	-1.971705000
C	0.218554000	-1.217931000	-0.334842000
C	0.301671000	-2.508458000	-0.132395000
H	1.289817000	-2.974271000	-0.229684000
H	-0.549767000	-3.138638000	0.135402000

TS1-A: E= -1191.748467

C	1.004382000	1.203531000	0.613501000
C	0.385413000	0.116828000	-0.280986000
H	0.898069000	0.922382000	1.674410000
H	0.488388000	2.160911000	0.468876000
H	0.522487000	0.329577000	-1.354810000
N	2.409917000	1.417552000	0.268005000
C	2.849630000	2.787388000	0.013083000
H	3.887473000	2.786150000	-0.331360000
H	2.218178000	3.250767000	-0.762036000
H	2.782446000	3.396727000	0.929988000
O	2.832389000	-0.815023000	0.428028000
O	4.503525000	0.648813000	-0.024302000
C	5.600796000	-0.351992000	-0.142984000
C	6.813133000	0.527354000	-0.443620000
H	6.989618000	1.243195000	0.372903000
H	7.710584000	-0.098764000	-0.554603000
H	6.667258000	1.090516000	-1.377521000
C	5.768211000	-1.083248000	1.189277000
H	5.923836000	-0.364382000	2.008277000
H	4.895454000	-1.706098000	1.422571000
H	6.655440000	-1.732432000	1.137301000
C	5.305092000	-1.297969000	-1.307083000
H	4.426587000	-1.924221000	-1.106799000
H	5.138031000	-0.730347000	-2.235549000
H	6.171390000	-1.957532000	-1.467551000
C	3.244324000	0.341864000	0.233890000
Au	-1.799048000	-0.024454000	-0.107164000
P	-4.141466000	0.009723000	0.016941000
C	-4.807406000	-0.720751000	1.560778000
H	-4.413270000	-0.174595000	2.430368000
H	-5.907397000	-0.665867000	1.566414000
H	-4.497272000	-1.773091000	1.639148000
C	-4.837927000	1.703629000	-0.054026000
H	-4.546236000	2.186310000	-0.998360000
H	-5.936754000	1.667329000	0.009696000
H	-4.447110000	2.303100000	0.781259000
C	-4.957985000	-0.904751000	-1.345737000
H	-4.649720000	-1.960336000	-1.323488000
H	-6.053083000	-0.845047000	-1.244521000
H	-4.659602000	-0.474269000	-2.312950000
C	0.743559000	-1.173105000	0.064443000
C	0.722347000	-2.444201000	0.360145000

H	1.654391000	-2.995392000	0.501018000
H	-0.230566000	-2.969180000	0.469249000

INT2-A: E= -1191.764575

C	0.921770000	1.487766000	0.347917000
C	0.379266000	0.209344000	-0.281204000
H	0.670321000	1.569952000	1.418537000
H	0.518469000	2.375416000	-0.159498000
H	0.568287000	0.271141000	-1.371073000
N	2.404056000	1.572776000	0.224177000
C	3.007168000	2.906068000	0.224142000
H	4.095611000	2.835142000	0.310455000
H	2.748178000	3.437545000	-0.705324000
H	2.610472000	3.474815000	1.078516000
O	2.599168000	-0.722246000	0.182551000
O	4.405951000	0.576924000	-0.034450000
C	5.421765000	-0.549312000	-0.145989000
C	6.720690000	0.230252000	-0.320968000
H	6.913173000	0.877301000	0.547262000
H	7.559355000	-0.474532000	-0.417427000
H	6.687736000	0.852956000	-1.226849000
C	5.415821000	-1.347276000	1.153959000
H	5.583066000	-0.689048000	2.019755000
H	4.479765000	-1.901809000	1.297058000
H	6.240260000	-2.075616000	1.123225000
C	5.100174000	-1.386544000	-1.380263000
H	4.173444000	-1.962981000	-1.265279000
H	5.023573000	-0.751125000	-2.275440000
H	5.923447000	-2.097861000	-1.545718000
C	3.125520000	0.469644000	0.119285000
Au	-1.736743000	0.035763000	-0.109427000
P	-4.091809000	-0.124421000	0.014959000
C	-4.751157000	-1.792693000	-0.380498000
H	-4.344579000	-2.530050000	0.327438000
H	-5.850763000	-1.803925000	-0.321581000
H	-4.441006000	-2.079838000	-1.396135000
C	-4.792316000	0.269231000	1.666711000
H	-4.513182000	1.294796000	1.950359000
H	-5.890169000	0.182708000	1.659071000
H	-4.380561000	-0.421466000	2.417417000
C	-4.974805000	1.000165000	-1.138018000
H	-4.675337000	0.776905000	-2.172733000
H	-6.065466000	0.881095000	-1.043153000
H	-4.704644000	2.043362000	-0.916968000
C	1.166716000	-0.942793000	0.230859000
C	0.829389000	-2.151282000	0.675199000
H	1.584460000	-2.883759000	0.966367000
H	-0.223398000	-2.422355000	0.741241000

INT3-A: E= -1034.449746

C	2.104040000	-1.064825000	-0.318145000
C	1.366894000	0.084974000	0.369534000
H	1.878529000	-1.093545000	-1.402635000
H	1.778227000	-2.031271000	0.100156000
H	1.558507000	-0.023430000	1.455389000
N	3.548093000	-0.958623000	-0.109168000
C	4.341085000	-2.166168000	-0.224537000
H	5.392809000	-1.917113000	-0.041799000
H	4.012068000	-2.920804000	0.511711000
H	4.248898000	-2.611127000	-1.233057000
O	3.407691000	1.364670000	0.000045000
O	5.385523000	0.376782000	0.168544000
C	4.191175000	0.243108000	0.033049000

Au	-0.729062000	-0.014233000	0.140715000
P	-3.085414000	-0.148532000	-0.093586000
C	-3.856919000	1.295244000	-0.936740000
H	-3.424876000	1.403320000	-1.942772000
H	-4.949070000	1.176737000	-1.020294000
H	-3.633580000	2.211271000	-0.369714000
C	-3.683660000	-1.588181000	-1.073207000
H	-3.351820000	-2.520554000	-0.592485000
H	-4.782324000	-1.591701000	-1.153747000
H	-3.245609000	-1.549809000	-2.081722000
C	-4.026323000	-0.279719000	1.483665000
H	-3.808156000	0.596138000	2.112801000
H	-5.111374000	-0.335336000	1.301220000
H	-3.702429000	-1.179335000	2.027887000
C	2.016329000	1.369491000	-0.051918000
C	1.448074000	2.524823000	-0.421353000
H	2.067333000	3.390766000	-0.664446000
H	0.363444000	2.614481000	-0.465775000

TS2-A: E= -1995.675473

C	2.335222000	-0.227470000	-0.866250000
C	1.453602000	-0.975897000	0.139710000
H	2.471628000	0.826369000	-0.561872000
H	1.848826000	-0.207200000	-1.853566000
H	1.304210000	-1.981397000	-0.318177000
N	3.622466000	-0.899525000	-1.000029000
C	4.381023000	-0.658796000	-2.214838000
H	5.314590000	-1.230704000	-2.166721000
H	3.802089000	-0.975245000	-3.099292000
H	4.624344000	0.413464000	-2.328669000
O	3.536203000	-1.618372000	1.221542000
O	5.340195000	-2.033367000	0.000834000
C	4.238561000	-1.542306000	0.043325000
Au	-0.807654000	-1.193535000	0.005898000
P	-3.102247000	-1.172137000	-0.244712000
C	-3.902362000	-0.246418000	1.117057000
H	-3.445680000	0.755274000	1.163184000
H	-4.986149000	-0.159256000	0.940760000
H	-3.725436000	-0.761715000	2.072551000
C	-3.550888000	-0.253871000	-1.765174000
H	-3.205378000	-0.808618000	-2.649850000
H	-4.640477000	-0.104867000	-1.824466000
H	-3.034798000	0.717921000	-1.742866000
C	-3.977787000	-2.780675000	-0.347957000
H	-3.801677000	-3.356344000	0.572671000
H	-5.060446000	-2.624517000	-0.477233000
H	-3.589425000	-3.358903000	-1.199192000
C	2.221813000	-1.225045000	1.403796000
C	1.766765000	-1.149329000	2.659249000
H	2.418776000	-1.395756000	3.499067000
H	0.745605000	-0.824343000	2.855614000
H	0.691869000	0.210517000	0.536582000
O	0.432551000	1.351307000	0.956748000
S	-0.510212000	2.201401000	0.075117000
O	-1.792394000	2.472284000	0.729195000
O	-0.526250000	1.738568000	-1.321991000
C	0.416299000	3.827616000	0.065934000
F	-0.250718000	4.700127000	-0.683682000
F	0.529326000	4.304927000	1.298511000
F	1.632067000	3.652795000	-0.449853000

3M: E= -438.545433

C	0.224356000	-1.532098000	-0.189351000

C	-1.103620000	-1.254551000	0.506460000
H	0.058124000	-1.767098000	-1.259474000
H	0.716990000	-2.409840000	0.260325000
H	-0.936571000	-1.220327000	1.598363000
N	1.109510000	-0.386509000	-0.039039000
C	2.535425000	-0.619076000	-0.189773000
H	3.063902000	0.330932000	-0.053068000
H	2.890547000	-1.345397000	0.561425000
H	2.769182000	-1.018450000	-1.193480000
O	-0.700678000	1.090821000	0.077586000
O	1.371644000	1.879312000	0.147026000
C	0.662145000	0.908109000	0.067577000
C	-1.627289000	0.069691000	0.043201000
C	-2.871968000	0.345373000	-0.356756000
H	-3.145040000	1.355599000	-0.666309000
H	-3.633574000	-0.434987000	-0.360598000
H	-1.832150000	-2.050617000	0.301042000

INT1-B: E= -1191.770067

C	-0.879299000	2.158298000	1.352449000
H	-0.998384000	2.798774000	2.241013000
H	-0.244461000	1.308967000	1.627718000
N	-2.183498000	1.656008000	0.964942000
C	-3.317076000	2.576140000	0.986765000
H	-4.250955000	2.018912000	1.114347000
H	-3.202127000	3.267311000	1.835160000
H	-3.395236000	3.169578000	0.058520000
O	-1.297644000	-0.372001000	0.370039000
O	-3.502349000	0.106429000	0.042212000
C	-3.925483000	-1.198026000	-0.509508000
C	-5.420294000	-0.989199000	-0.752496000
H	-5.588662000	-0.156638000	-1.451618000
H	-5.863483000	-1.898923000	-1.183632000
H	-5.941671000	-0.763537000	0.189663000
C	-3.193700000	-1.458009000	-1.827730000
H	-3.355946000	-0.625709000	-2.529648000
H	-2.114486000	-1.582369000	-1.672327000
H	-3.588269000	-2.374217000	-2.292620000
C	-3.692579000	-2.300663000	0.525185000
H	-2.623400000	-2.458450000	0.715298000
H	-4.187468000	-2.048071000	1.475517000
H	-4.126246000	-3.243964000	0.159854000
C	-2.262679000	0.385056000	0.440328000
C	-0.196437000	2.984188000	0.268675000
C	0.824496000	2.639032000	-0.474345000
C	1.866440000	2.477587000	-1.313034000
H	1.699332000	2.197136000	-2.361305000
H	2.854437000	2.880645000	-1.056535000
Au	1.762922000	0.435951000	-0.299583000
P	1.921723000	-1.785198000	0.432405000
C	1.243441000	-1.984386000	2.118469000
H	0.203337000	-1.626796000	2.108675000
H	1.272638000	-3.044214000	2.416469000
H	1.827861000	-1.389380000	2.835383000
C	3.624921000	-2.454666000	0.466897000
H	3.615172000	-3.499762000	0.814027000
H	4.062016000	-2.413500000	-0.541609000
H	4.249263000	-1.854082000	1.144614000
C	0.940046000	-2.904442000	-0.627003000
H	1.325541000	-2.885795000	-1.656740000
H	0.984393000	-3.934269000	-0.239499000
H	-0.099509000	-2.546287000	-0.623207000
H	-0.596683000	3.989557000	0.072915000

TS1-B: E= -1191.742739

C	2.690218000	-2.448521000	1.165131000
H	3.380539000	-3.250587000	1.451806000
H	2.007557000	-2.276814000	2.016087000
N	3.478743000	-1.248104000	0.903034000
C	4.916550000	-1.272055000	1.157207000
H	5.354482000	-0.309065000	0.878830000
H	5.114428000	-1.456188000	2.225543000
H	5.397182000	-2.068360000	0.566484000
O	1.586257000	-0.275155000	0.143130000
O	3.537883000	0.898094000	0.230451000
C	3.043172000	2.167646000	-0.367911000
C	4.292754000	3.046177000	-0.352990000
H	5.094787000	2.596699000	-0.957000000
H	4.058864000	4.036077000	-0.771432000
H	4.662724000	3.182577000	0.673934000
C	2.568455000	1.909416000	-1.797830000
H	3.361439000	1.426427000	-2.389033000
H	1.670726000	1.278484000	-1.819072000
H	2.328752000	2.869694000	-2.279197000
C	1.948081000	2.754205000	0.523844000
H	1.051450000	2.120447000	0.527576000
H	2.310335000	2.869248000	1.556855000
H	1.672538000	3.752022000	0.149708000
C	2.806323000	-0.185268000	0.406472000
C	1.891781000	-2.867362000	-0.053782000
C	1.006671000	-2.118784000	-0.664205000
C	-0.078843000	-1.830601000	-1.467382000
H	0.140207000	-1.334280000	-2.423452000
H	-0.753524000	-2.699252000	-1.553193000
Au	-1.495757000	-0.543246000	-0.404464000
P	-3.136224000	0.783998000	0.595224000
C	-3.175392000	0.653910000	2.424272000
H	-2.203483000	0.960696000	2.837880000
H	-3.966815000	1.296728000	2.840696000
H	-3.365220000	-0.389322000	2.716513000
C	-4.836271000	0.367193000	0.050177000
H	-5.571808000	1.021101000	0.544386000
H	-4.916390000	0.489579000	-1.039971000
H	-5.057031000	-0.681081000	0.299614000
C	-2.949501000	2.575477000	0.249244000
H	-2.996288000	2.749420000	-0.835855000
H	-3.749042000	3.149723000	0.743078000
H	-1.972973000	2.923101000	0.616998000
H	2.066438000	-3.855964000	-0.496886000

INT2-B: E= -1191.768517

C	2.947234000	-2.755418000	0.720124000
H	3.677654000	-3.493097000	0.341366000
H	2.705548000	-3.063323000	1.756491000
N	3.631644000	-1.449122000	0.782823000
C	4.915761000	-1.437696000	1.486457000
H	5.314326000	-0.421120000	1.540880000
H	4.772018000	-1.831541000	2.504450000
H	5.629573000	-2.086603000	0.955433000
O	1.965689000	-0.353911000	-0.376450000
O	3.749263000	0.757237000	0.372799000
C	3.363839000	2.116878000	-0.204181000
C	4.536552000	2.978825000	0.248977000
H	5.483892000	2.609304000	-0.169931000
H	4.385578000	4.010648000	-0.100221000
H	4.615234000	2.994480000	1.345788000
C	3.299580000	2.006097000	-1.723654000

H	4.235844000	1.592329000	-2.127418000
H	2.455032000	1.393143000	-2.062690000
H	3.173995000	3.016194000	-2.142072000
C	2.053138000	2.563359000	0.435189000
H	1.204229000	1.940207000	0.124455000
H	2.130386000	2.553445000	1.532951000
H	1.848969000	3.599103000	0.123839000
C	3.106438000	-0.355118000	0.253809000
C	1.732587000	-2.701811000	-0.148257000
C	1.238010000	-1.564512000	-0.650915000
C	0.046887000	-1.291797000	-1.457492000
H	0.305591000	-0.637227000	-2.306330000
H	-0.336498000	-2.241711000	-1.858362000
Au	-1.569111000	-0.372914000	-0.426129000
P	-3.469506000	0.577128000	0.618567000
C	-4.026399000	2.166682000	-0.116722000
H	-4.251054000	2.019297000	-1.183486000
H	-4.926716000	2.542134000	0.394501000
H	-3.224175000	2.914711000	-0.033418000
C	-3.274872000	0.950645000	2.408073000
H	-4.201065000	1.377360000	2.824003000
H	-3.027417000	0.026867000	2.951784000
H	-2.452402000	1.667361000	2.549601000
C	-4.956550000	-0.499574000	0.543276000
H	-4.745536000	-1.455555000	1.044986000
H	-5.818112000	-0.016272000	1.030359000
H	-5.205392000	-0.709625000	-0.507548000
H	1.218535000	-3.639386000	-0.362372000

INT3-B: E= -1034.459029

C	4.076564000	-0.660360000	1.163490000
H	5.000201000	-1.266778000	1.041943000
H	4.054549000	-0.379815000	2.239509000
N	4.200938000	0.552374000	0.364167000
C	5.354183000	1.382425000	0.647864000
H	5.335953000	2.256680000	-0.012055000
H	5.343934000	1.719121000	1.700759000
H	6.290992000	0.819541000	0.482121000
O	2.254668000	0.140982000	-0.857064000
O	3.447533000	1.962473000	-1.268409000
C	3.329526000	0.951440000	-0.615686000
C	2.865520000	-1.449439000	0.789398000
C	2.015183000	-1.037193000	-0.163601000
C	0.765715000	-1.694489000	-0.613062000
H	0.785761000	-1.806667000	-1.710823000
H	0.686881000	-2.695910000	-0.164339000
Au	-0.962712000	-0.569225000	-0.171018000
P	-2.893375000	0.728331000	0.280072000
C	-4.497094000	-0.173188000	0.190400000
H	-4.494580000	-0.994607000	0.922387000
H	-5.348322000	0.495441000	0.396206000
H	-4.612749000	-0.610634000	-0.812479000
C	-3.132436000	2.146214000	-0.870216000
H	-4.035673000	2.724464000	-0.617807000
H	-2.251920000	2.804203000	-0.822668000
H	-3.217488000	1.768748000	-1.900169000
C	-2.939072000	1.515573000	1.944591000
H	-2.057988000	2.163717000	2.063408000
H	-3.853148000	2.115003000	2.082995000
H	-2.895168000	0.735069000	2.718805000
H	2.670948000	-2.383948000	1.317126000

TS2-B: E= -1995.681633

C	-0.626692000	4.218974000	-0.083647000
H	-0.763175000	5.034246000	-0.826942000
H	-0.155853000	4.692401000	0.797036000
N	-1.932049000	3.700432000	0.331880000
C	-2.740640000	4.568779000	1.168246000
H	-3.694804000	4.073449000	1.379709000
H	-2.221050000	4.785679000	2.117658000
H	-2.937769000	5.528487000	0.657926000
O	-1.655880000	1.766772000	-0.917730000
O	-3.550079000	2.082743000	0.195516000
C	-2.455218000	2.518659000	-0.095505000
C	0.254689000	3.130190000	-0.609561000
C	-0.279092000	1.968499000	-0.995447000
C	0.422158000	0.732867000	-1.441653000
H	1.467695000	0.959679000	-1.706866000
H	-0.026948000	0.330260000	-2.367754000
Au	-0.742178000	-0.983329000	-0.450006000
P	-2.400327000	-2.290536000	0.465461000
C	-2.498374000	-4.037015000	-0.087896000
H	-1.562750000	-4.555933000	0.167435000
H	-3.344924000	-4.551653000	0.393708000
H	-2.626796000	-4.069425000	-1.179760000
C	-4.041535000	-1.557965000	0.102968000
H	-4.832962000	-2.063147000	0.678990000
H	-4.023049000	-0.480755000	0.336803000
H	-4.250416000	-1.662022000	-0.972333000
C	-2.300214000	-2.377582000	2.294294000
H	-2.329541000	-1.357628000	2.704722000
H	-3.140522000	-2.960751000	2.703086000
H	-1.348722000	-2.842435000	2.591206000
H	1.337887000	3.258248000	-0.628002000
H	1.034570000	0.140100000	-0.366423000
O	1.817646000	-0.057246000	0.681414000
S	3.201135000	0.617552000	0.697984000
O	3.707623000	0.822292000	2.048118000
O	3.282519000	1.710693000	-0.284831000
C	4.250367000	-0.742485000	-0.045155000
F	3.756420000	-1.091581000	-1.241511000
F	5.503345000	-0.325946000	-0.209962000
F	4.255232000	-1.820382000	0.737884000

4M: E= -438.552335

C	0.427436000	1.593976000	-0.000045000
H	0.725275000	2.198603000	0.882667000
H	0.725256000	2.198504000	-0.882833000
N	1.181077000	0.345768000	0.000024000
C	2.625829000	0.482028000	0.000049000
H	3.079089000	-0.515167000	0.000099000
H	2.964222000	1.036388000	-0.893727000
H	2.964179000	1.036465000	0.893793000
O	-0.731387000	-0.997695000	-0.000048000
O	1.277186000	-1.938727000	-0.000010000
C	0.642743000	-0.913537000	-0.000012000
C	-1.046129000	1.351403000	-0.000013000
C	-1.545959000	0.111850000	-0.000013000
C	-2.987740000	-0.278181000	0.000032000
H	-3.637291000	0.607278000	0.000206000
H	-3.216558000	-0.893283000	0.885949000
H	-1.718505000	2.210190000	0.000004000
H	-3.216674000	-0.893207000	-0.885846000

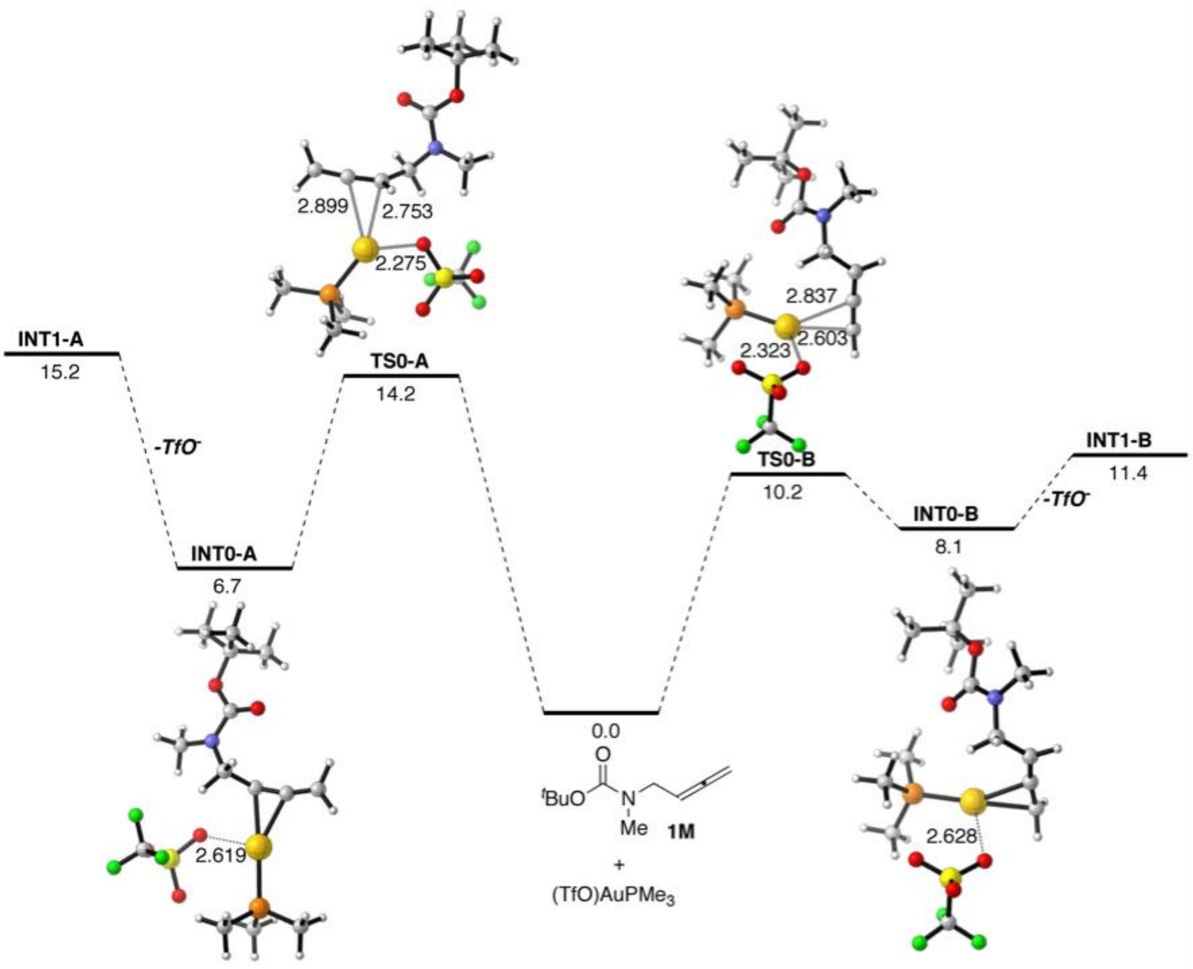

Figure S1: Computed reaction profile for the conversion of the starting material **1M** into **INT1-A** and **INT1-B** in presence of $AuPMe_3^+$.

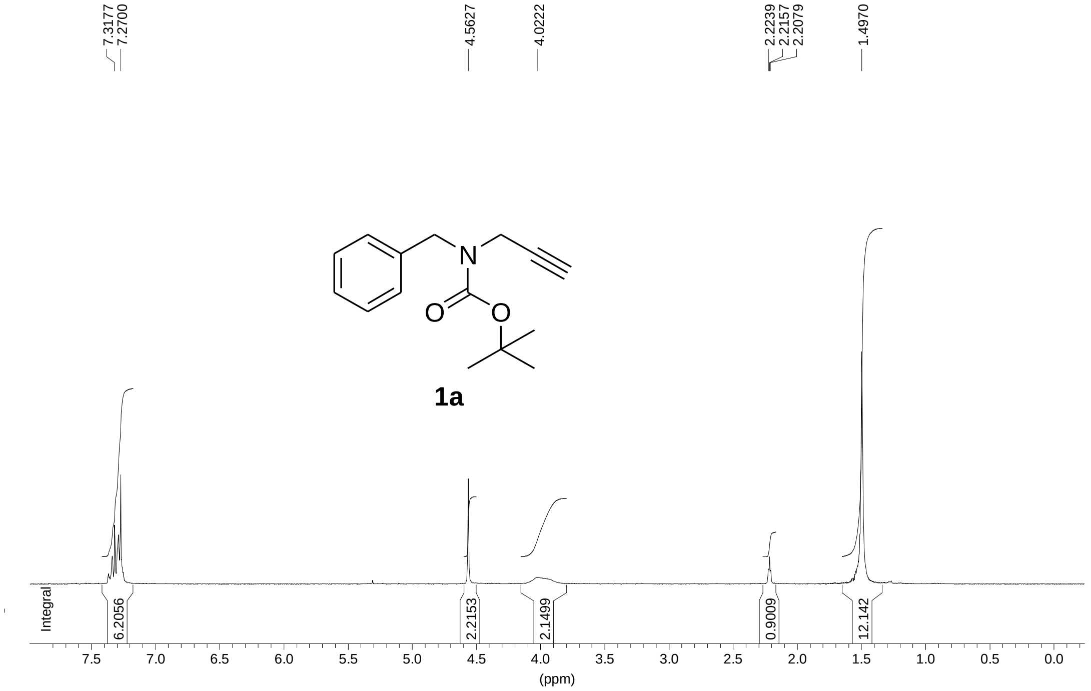
7.3177
7.2700
4.5627
4.0222
2.2239
2.2157
2.2079
1.4970
1a
Integral
6.2056
2.2153
2.1499
0.9009
12.142
(ppm)

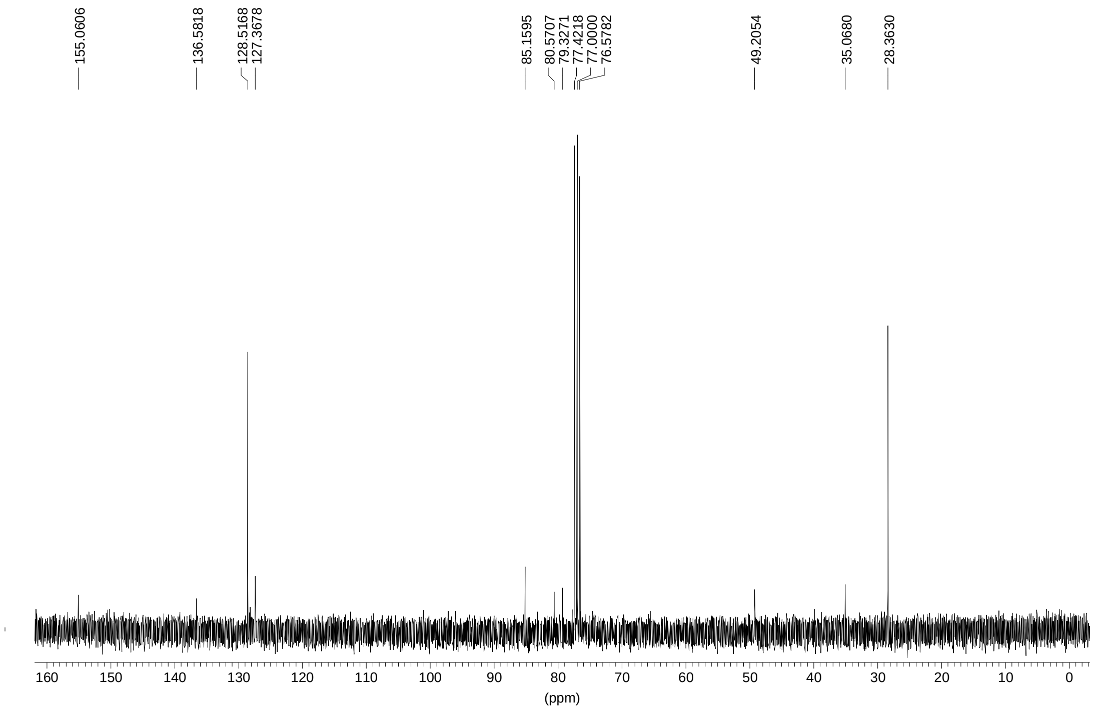
155.0606
136.5818
128.5168
127.3678
85.1595
80.5707
79.3271
77.4218
77.0000
76.5782
49.2054
35.0680
28.3630
(ppm)

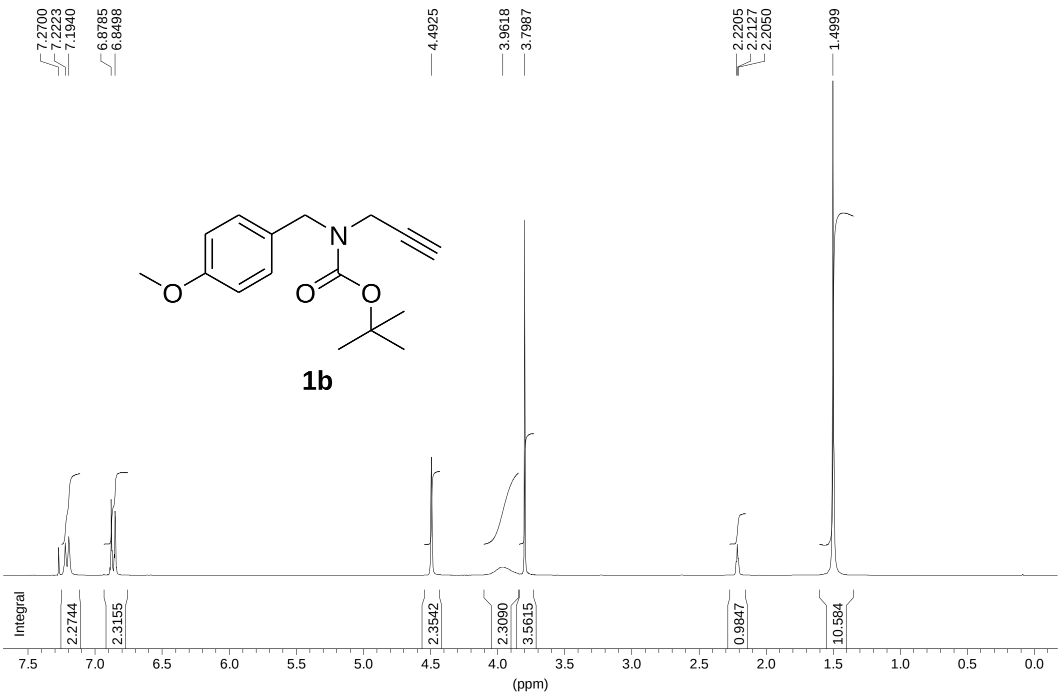
7.2700
7.2223
7.1940
6.8785
6.8498
4.4925
3.9618
3.7987
2.2205
2.2127
2.2050
1.4999
N
O
O
O
1b
Integral
2.2744
2.3155
2.3542
2.3090
3.5615
0.9847
10.584
7.5 7.0 6.5 6.0 5.5 5.0 4.5 4.0 3.5 3.0 2.5 2.0 1.5 1.0 0.5 0.0
(ppm)

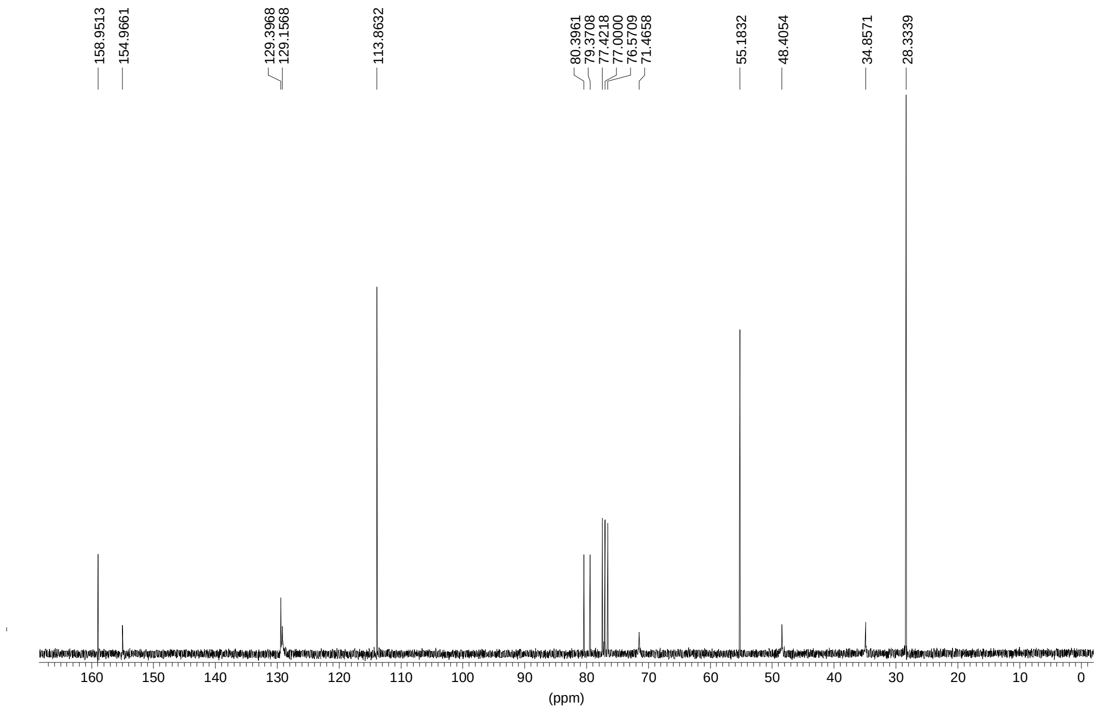
158.9513
154.9661
129.3968
129.1568
113.8632
80.3961
79.3708
77.4218
77.0000
76.5709
71.4658
55.1832
48.4054
34.8571
28.3339
160 150 140 130 120 110 100 90 80 70 60 50 40 30 20 10 0
(ppm)

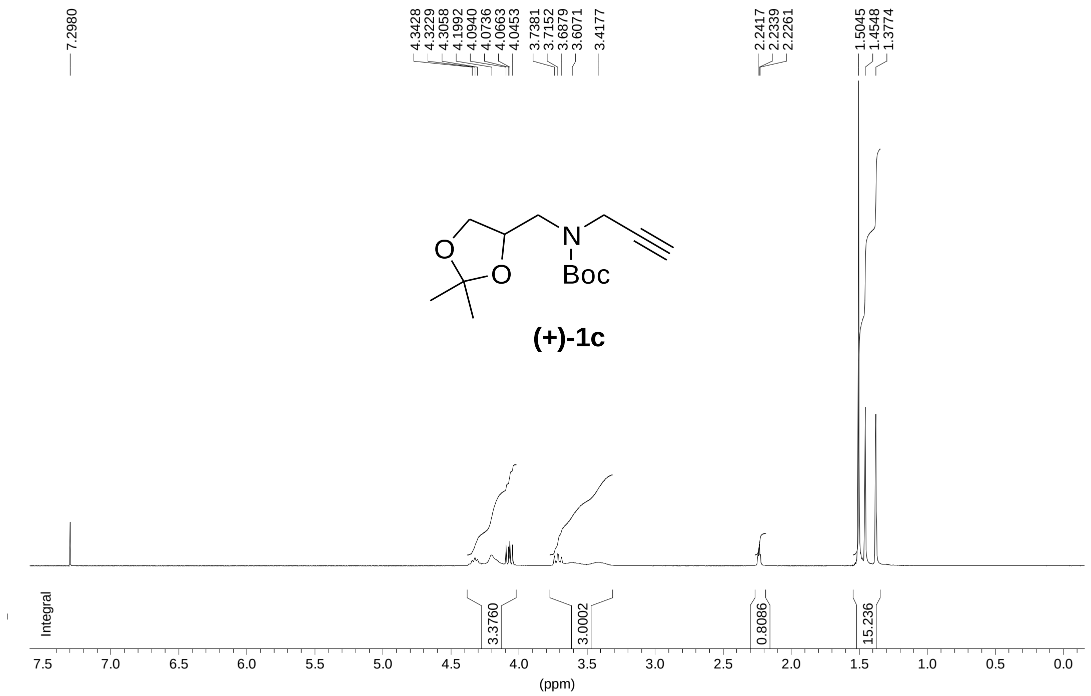
7.2980
4.3428 4.3229 4.3058 4.1992 4.0940 4.0736 4.0663 4.0453 3.7381 3.7152 3.6879 3.6071 3.4177
2.2417 2.2339 2.2261
1.5045 1.4548 1.3774
O
O
N
Boc
(+)-1c
Integral
3.3760 3.0002 0.8086 15.236
(ppm)

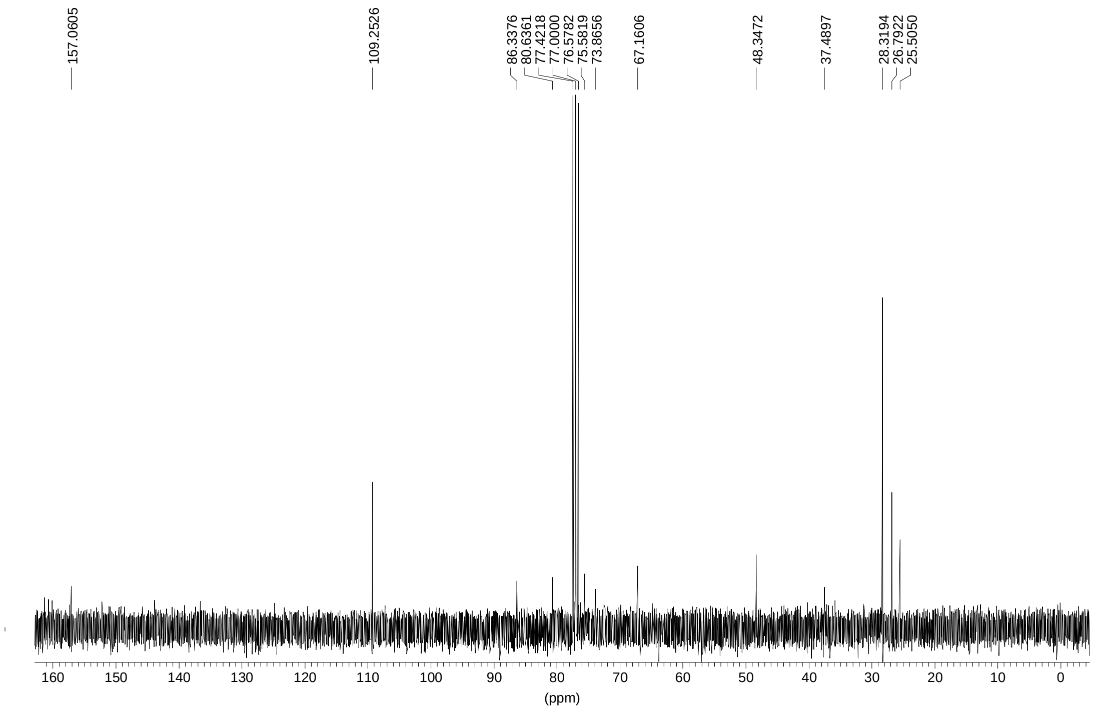
157.0605
109.2526
86.3376 80.6361 77.4218 77.0000 76.5782 75.5819 73.8656
67.1606
48.3472
37.4897
28.3194 26.7922 25.5050
(ppm)

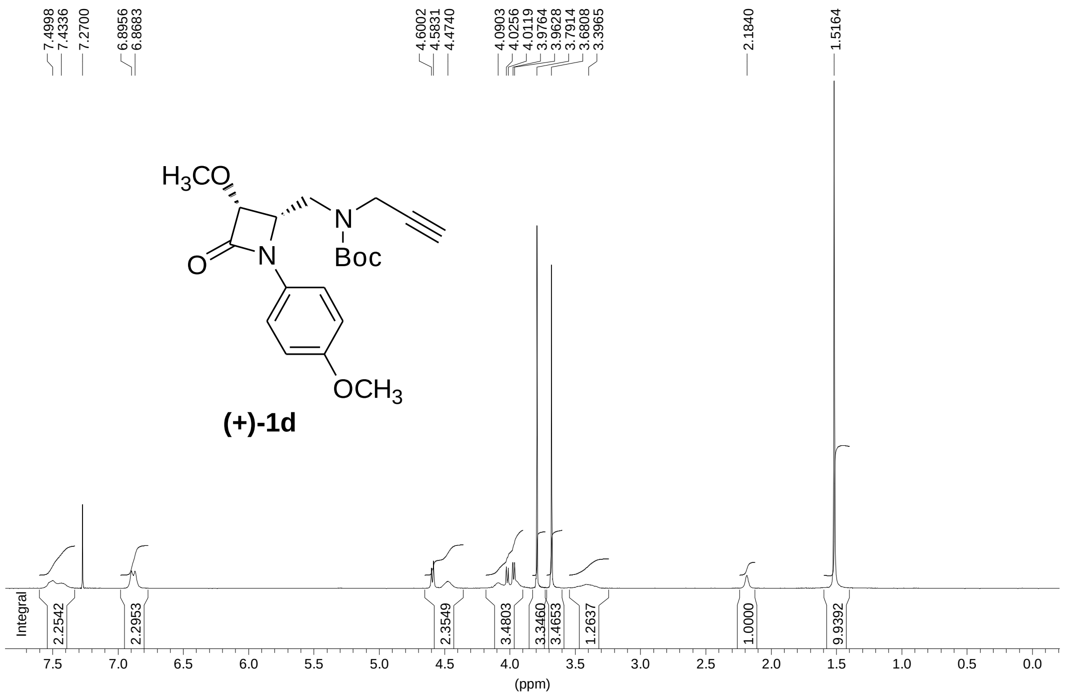
7.4998
7.4336
7.2700
6.8956
6.8683
4.6002
4.5831
4.4740
4.0903
4.0256
4.0119
3.9764
3.9628
3.7914
3.6808
3.3965
2.1840
1.5164
H_3CO
N
Boc
O
N
OCH_3
(+)-1d
Integral
2.2542
2.2953
2.3549
3.4803
3.3460
3.4653
1.2637
1.0000
9.9392
7.5 7.0 6.5 6.0 5.5 5.0 4.5 4.0 3.5 3.0 2.5 2.0 1.5 1.0 0.5 0.0
(ppm)

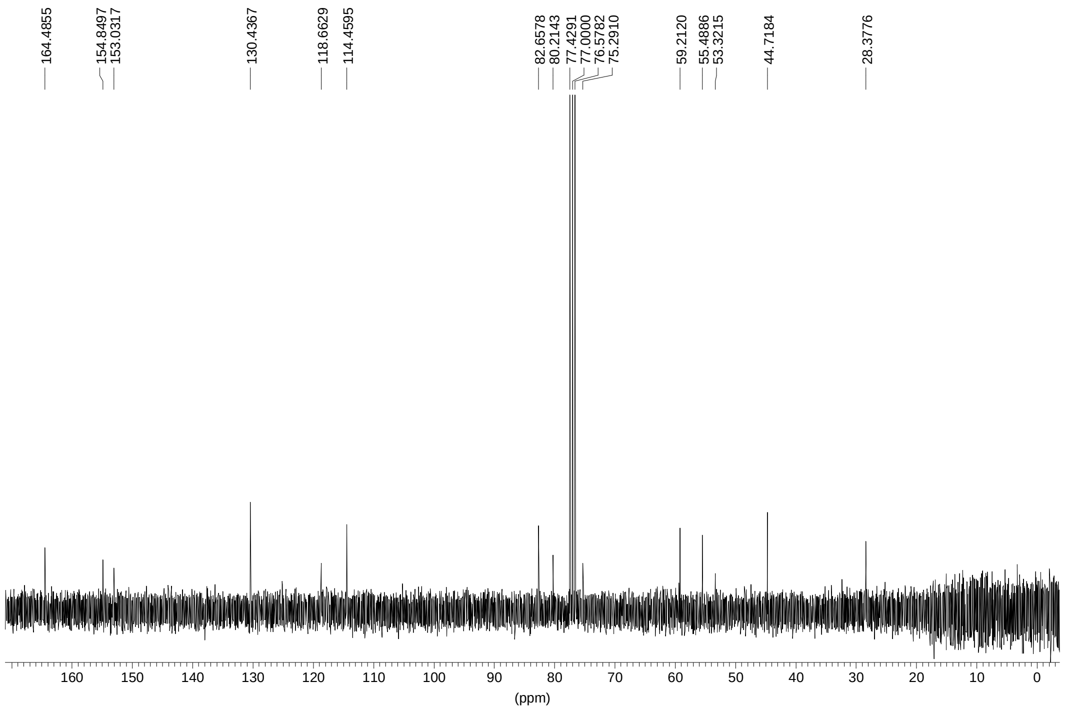
164.4855
154.8497
153.0317
130.4367
118.6629
114.4595
82.6578
80.2143
77.4291
77.0000
76.5782
75.2910
59.2120
55.4886
53.3215
44.7184
28.3776
160 150 140 130 120 110 100 90 80 70 60 50 40 30 20 10 0
(ppm)

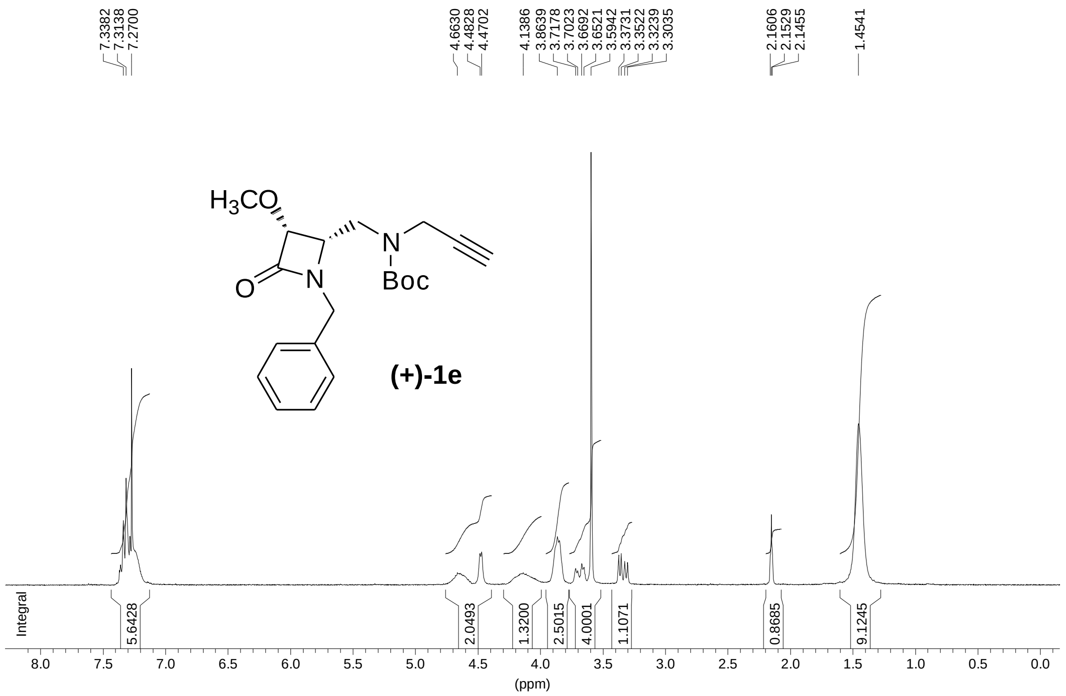
7.3382
7.3138
7.2700
4.6630
4.4828
4.4702
4.1386
3.8639
3.7178
3.7023
3.6692
3.6521
3.5942
3.3731
3.3522
3.3239
3.3035
2.1606
2.1529
2.1455
1.4541
H_3CO
N
Boc
O
N
(+)-1e
Integral
5.6428
2.0493
1.3200
2.5015
4.0001
1.1071
0.8685
9.1245
(ppm)

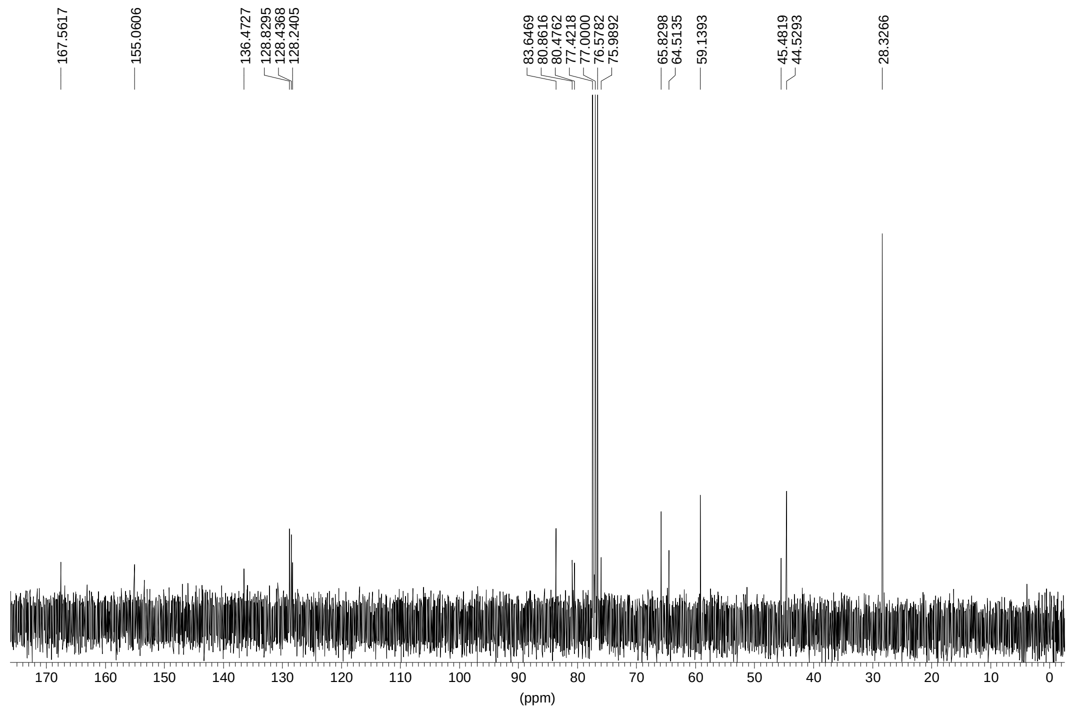
167.5617
155.0606
136.4727
128.8295
128.4368
128.2405
83.6469
80.8616
80.4762
77.4218
77.0000
76.5782
75.9892
65.8298
64.5135
59.1393
45.4819
44.5293
28.3266
(ppm)

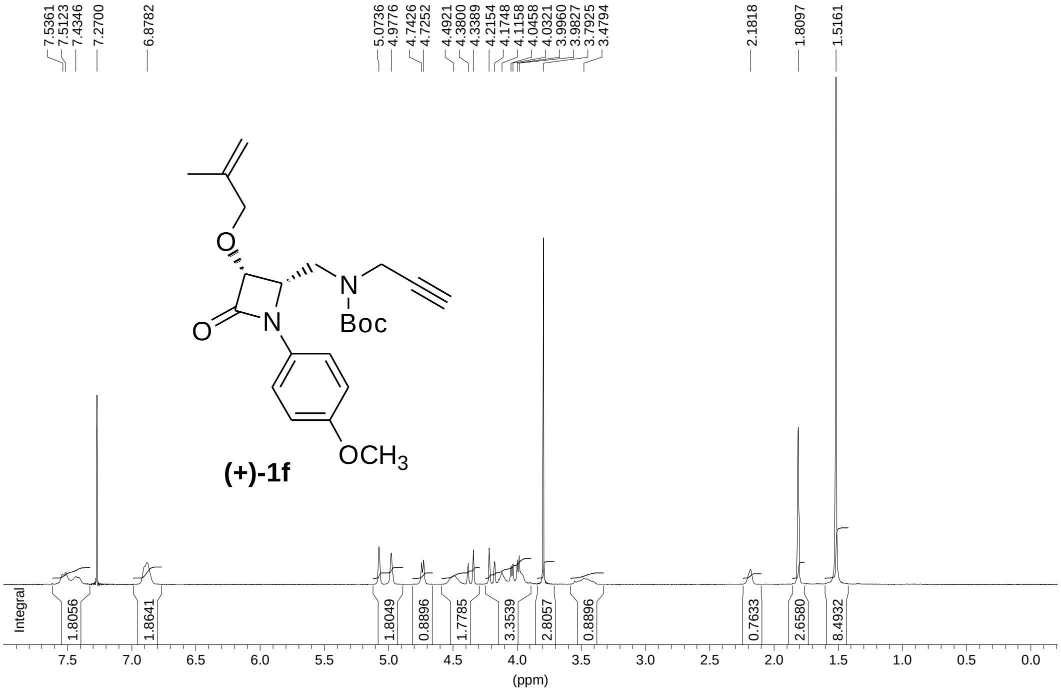
7.5361
7.5123
7.4346
7.2700
6.8782
5.0736
4.9776
4.7426
4.7252
4.4921
4.3800
4.3389
4.2154
4.1748
4.1158
4.0458
4.0321
3.9960
3.9827
3.7925
3.4794
2.1818
1.8097
1.5161
O
N
Boc
OCH3
(+)-1f
Integral
1.8056
1.8641
1.8049
0.8896
1.7785
3.3539
2.8057
0.8896
0.7633
2.6580
8.4932
(ppm)

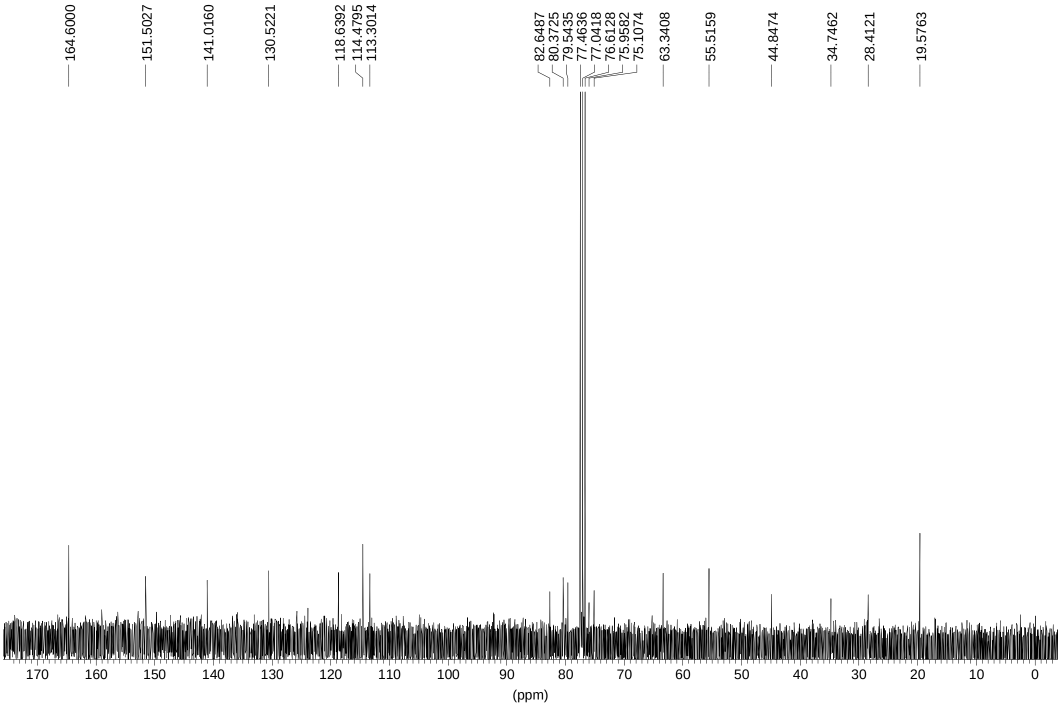
164.6000
151.5027
141.0160
130.5221
118.6392
114.4795
113.3014
82.6487
80.3725
79.5435
77.4636
77.0418
76.6128
75.9582
75.1074
63.3408
55.5159
44.8474
34.7462
28.4121
19.5763
(ppm)

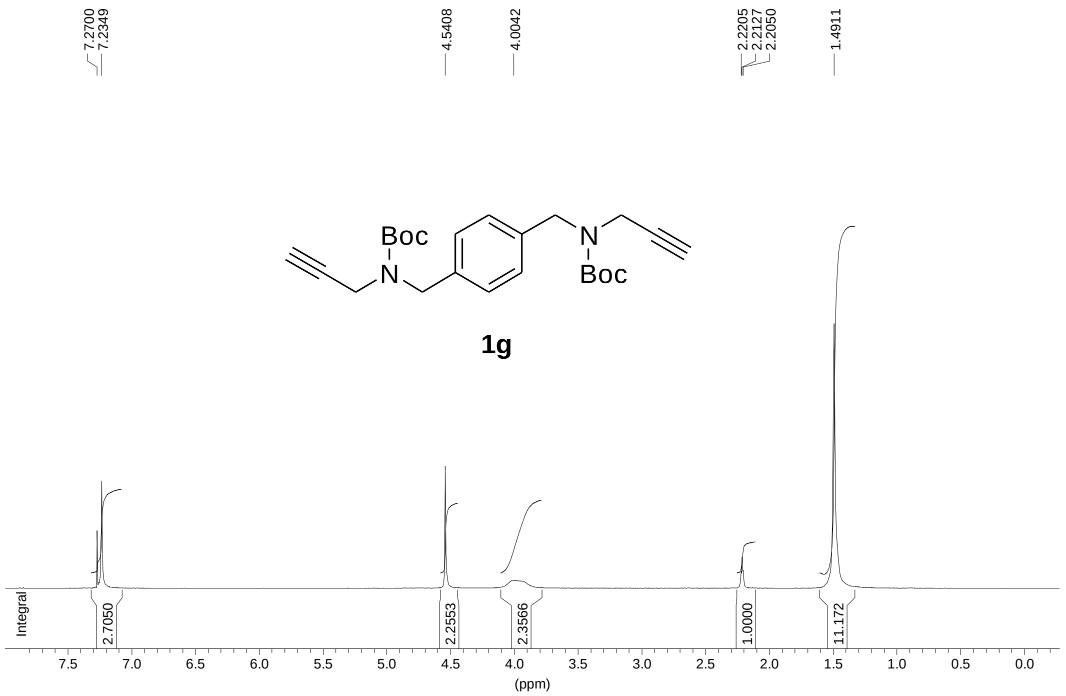

7.2700
7.2349
4.5408
4.0042
2.2205
2.2127
2.2050
1.4911
Boc
N
Boc
1g
Integral
2.7050
2.2553
2.3566
1.0000
11.172
(ppm)

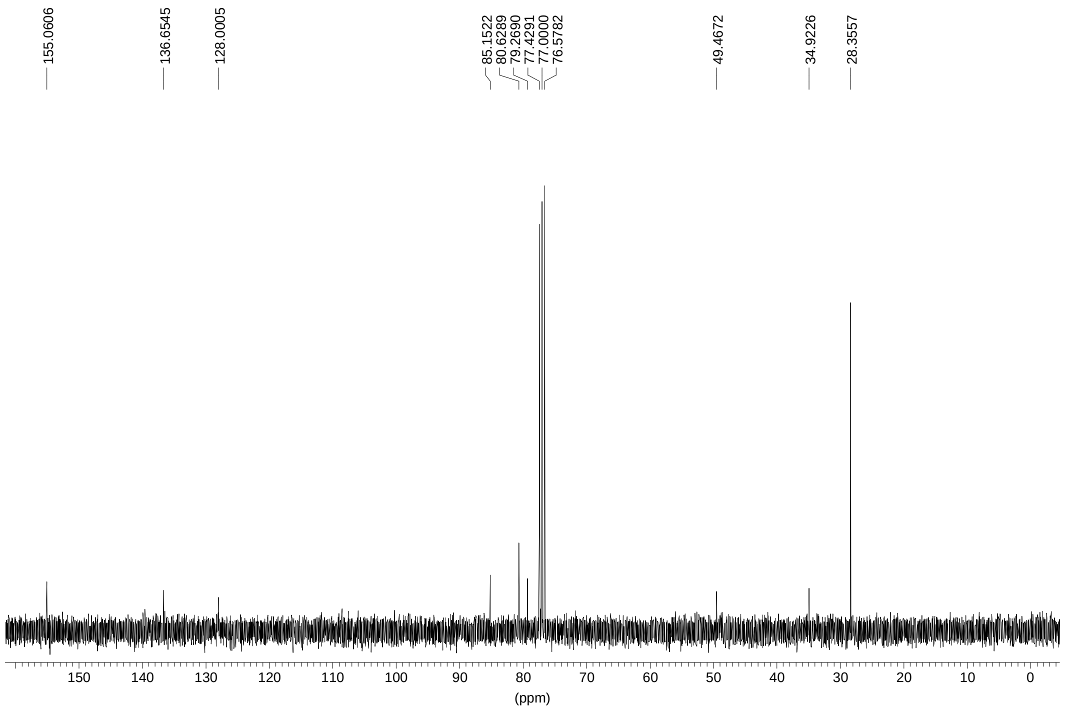

155.0606
136.6545
128.0005
85.1522
80.6289
79.2690
77.4291
77.0000
76.5782
49.4672
34.9226
28.3557
(ppm)

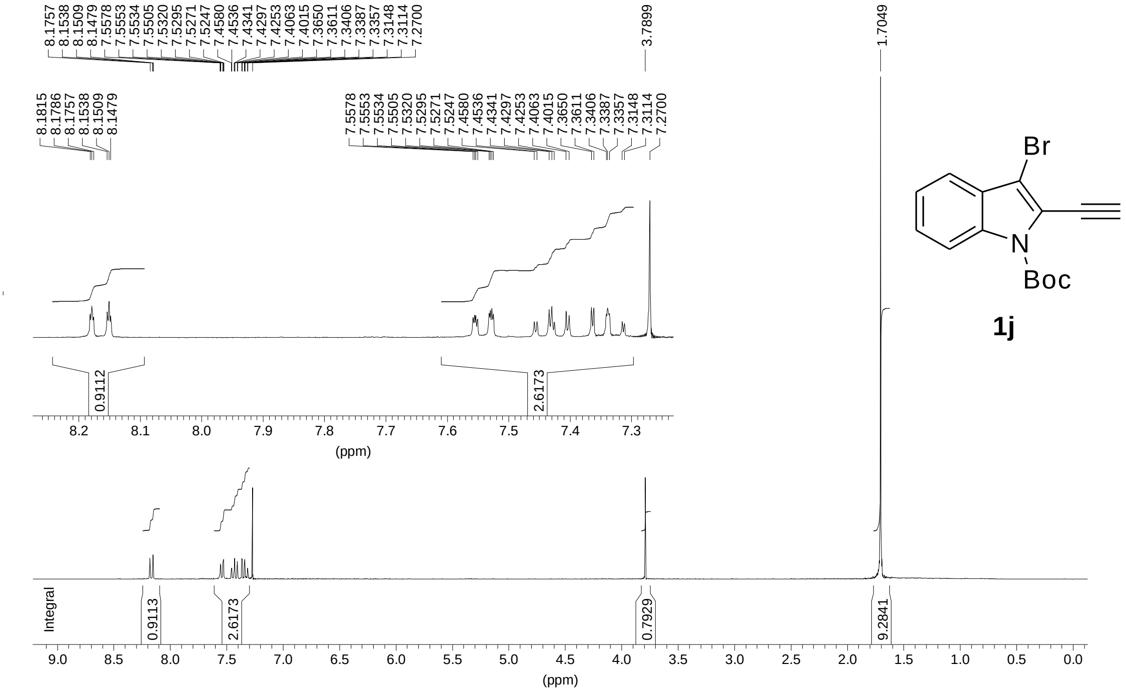
8.1757 8.1538 8.1509 8.1479 7.5578 7.5553 7.5534 7.5505 7.5320 7.5295 7.5271 7.5247 7.4580 7.4536 7.4341 7.4297 7.4253 7.4063 7.4015 7.3650 7.3611 7.3406 7.3387 7.3357 7.3148 7.3114 7.2700
3.7899
1.7049
8.1815 8.1786 8.1757 8.1538 8.1509 8.1479
7.5578 7.5553 7.5534 7.5505 7.5320 7.5295 7.5271 7.5247 7.4580 7.4536 7.4341 7.4297 7.4253 7.4063 7.4015 7.3650 7.3611 7.3406 7.3387 7.3357 7.3148 7.3114 7.2700
0.9112
2.6173
8.2 8.1 8.0 7.9 7.8 7.7 7.6 7.5 7.4 7.3
(ppm)
Integral
0.9113
2.6173
0.7929
9.2841
9.0 8.5 8.0 7.5 7.0 6.5 6.0 5.5 5.0 4.5 4.0 3.5 3.0 2.5 2.0 1.5 1.0 0.5 0.0
(ppm)
Br
N
Boc

1j

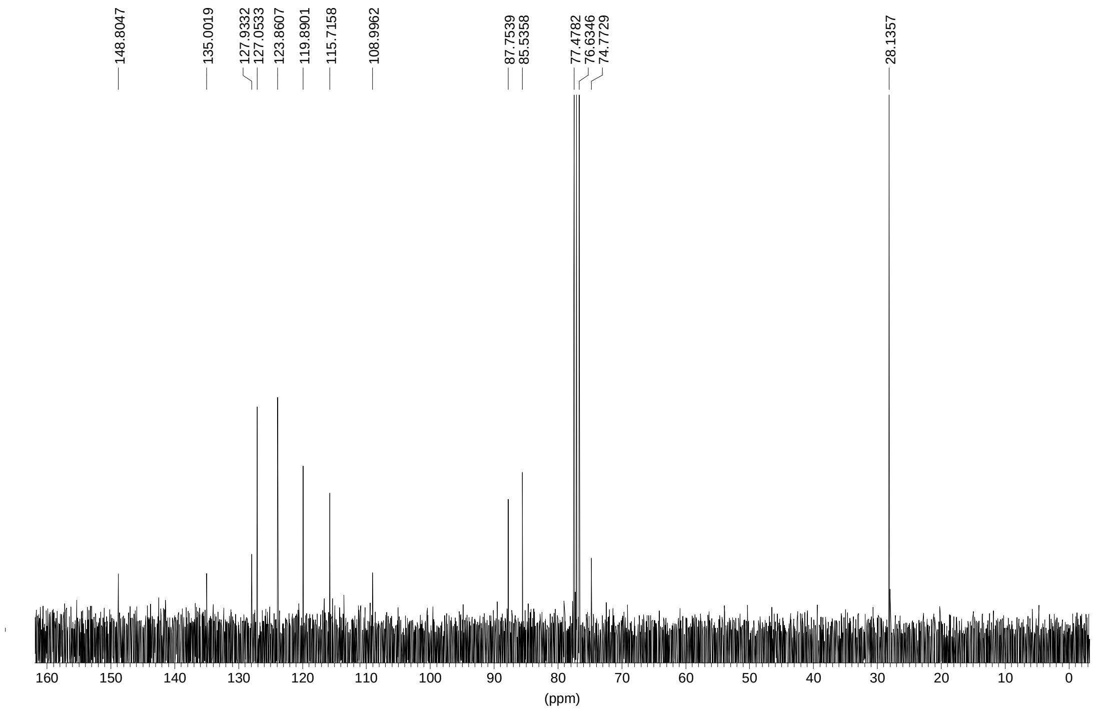
148.8047
135.0019
127.9332
127.0533
123.8607
119.8901
115.7158
108.9962
87.7539
85.5358
77.4782
76.6346
74.7729
28.1357
160 150 140 130 120 110 100 90 80 70 60 50 40 30 20 10 0
(ppm)

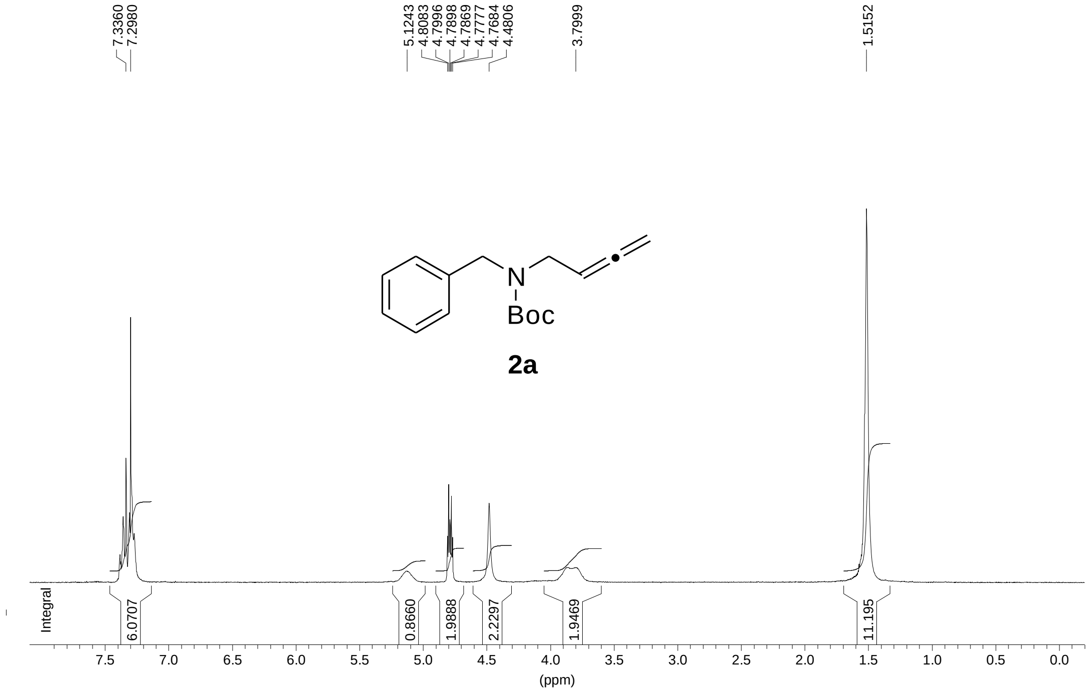
7.3360
7.2980
5.1243
4.8083
4.7996
4.7898
4.7869
4.7777
4.7684
4.4806
3.7999
1.5152
N
Boc
2a
Integral
6.0707
0.8660
1.9888
2.2297
1.9469
11.195
7.5 7.0 6.5 6.0 5.5 5.0 4.5 4.0 3.5 3.0 2.5 2.0 1.5 1.0 0.5 0.0
(ppm)

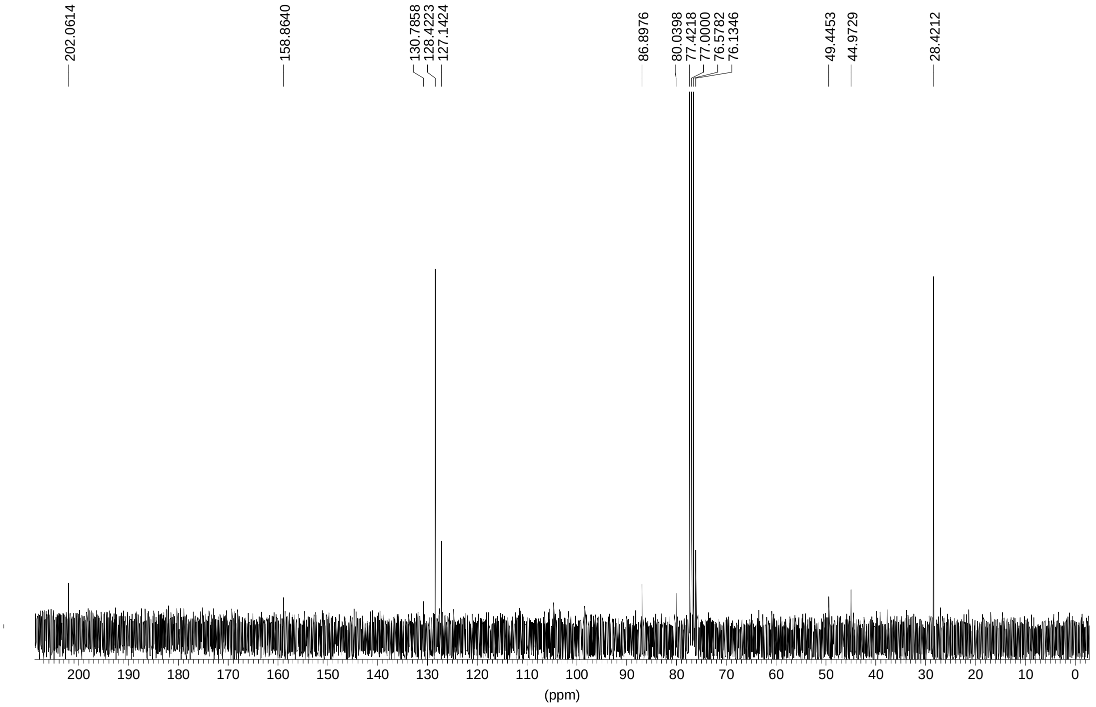
202.0614
158.8640
130.7858
128.4223
127.1424
86.8976
80.0398
77.4218
77.0000
76.5782
76.1346
49.4453
44.9729
28.4212
200 190 180 170 160 150 140 130 120 110 100 90 80 70 60 50 40 30 20 10 0
(ppm)

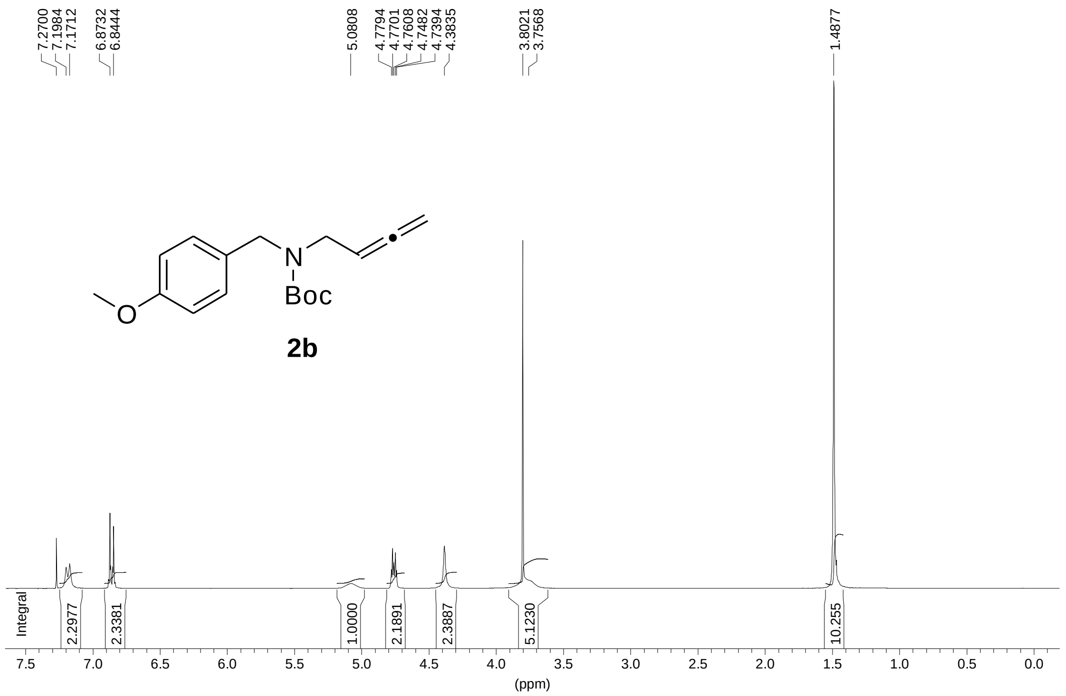
7.2700
7.1984
7.1712
6.8732
6.8444
5.0808
4.7794
4.7701
4.7608
4.7482
4.7394
4.3835
3.8021
3.7568
1.4877
N
Boc
O
2b
Integral
2.2977
2.3381
1.0000
2.1891
2.3887
5.1230
10.255
7.5 7.0 6.5 6.0 5.5 5.0 4.5 4.0 3.5 3.0 2.5 2.0 1.5 1.0 0.5 0.0
(ppm)

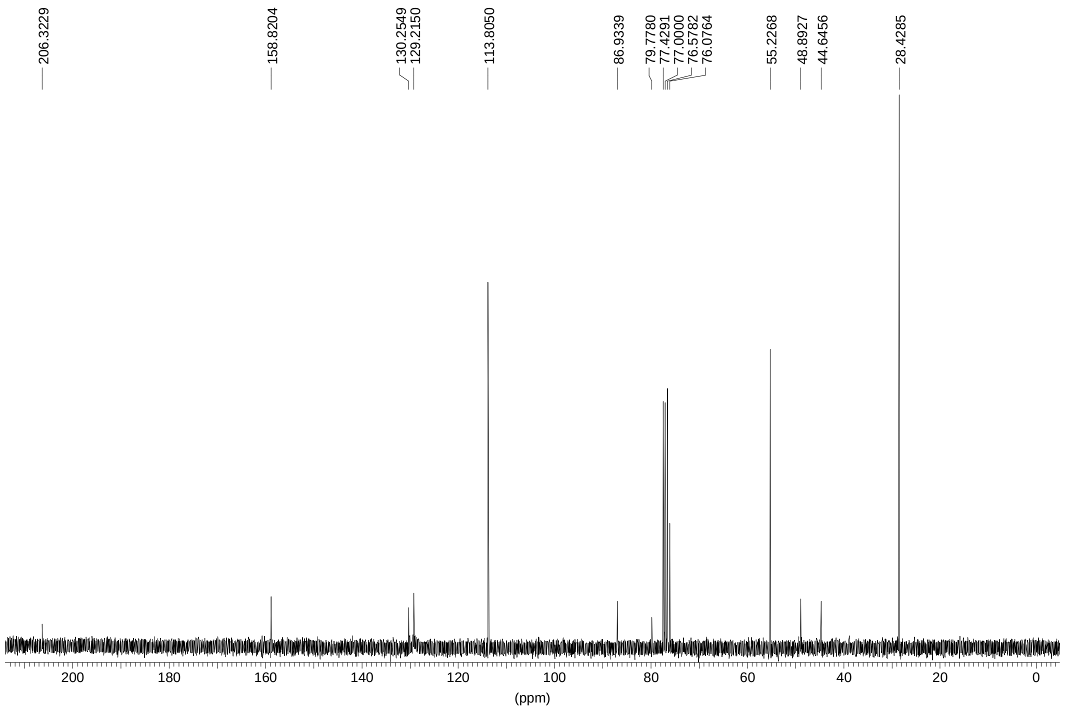
206.3229
158.8204
130.2549
129.2150
113.8050
86.9339
79.7780
77.4291
77.0000
76.5782
76.0764
55.2268
48.8927
44.6456
28.4285
200 180 160 140 120 100 80 60 40 20 0
(ppm)

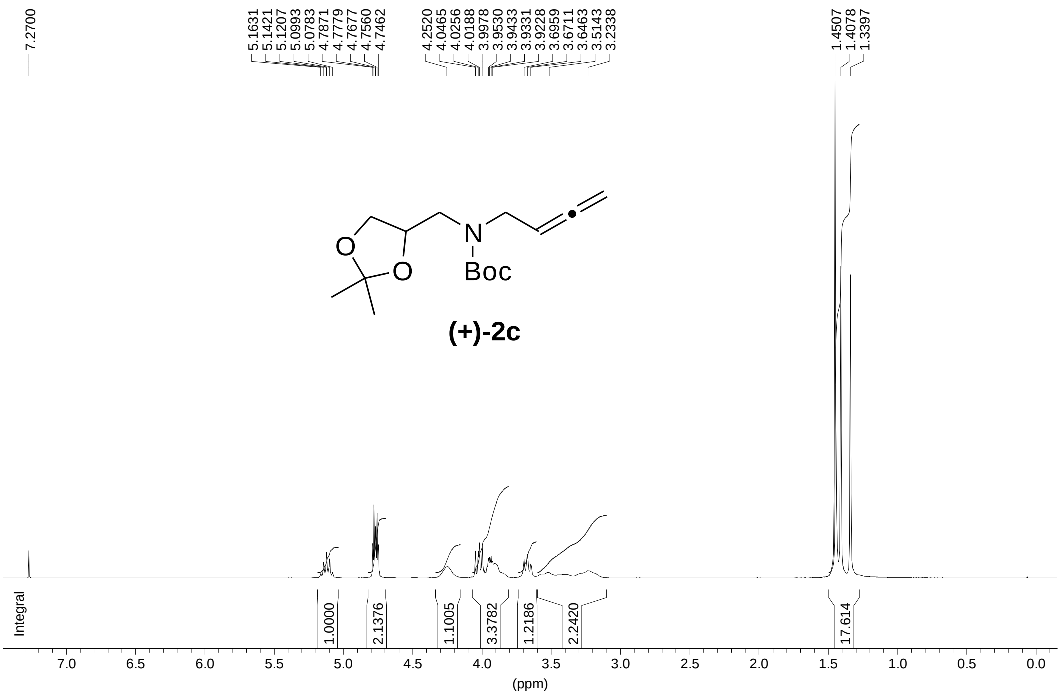

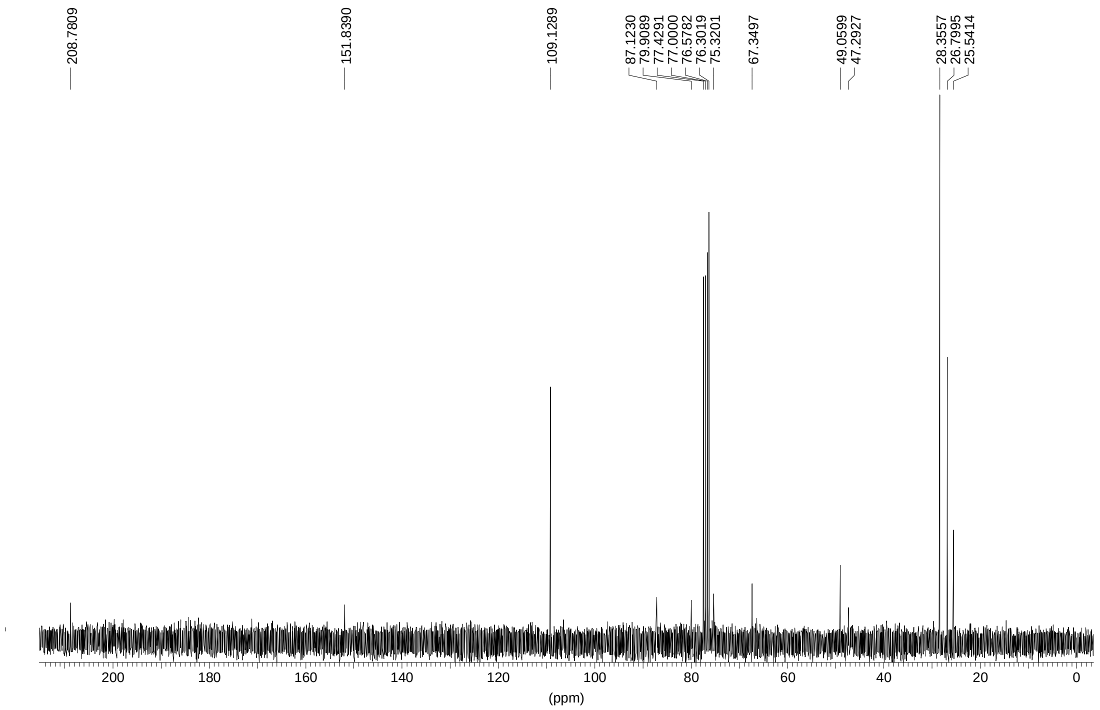

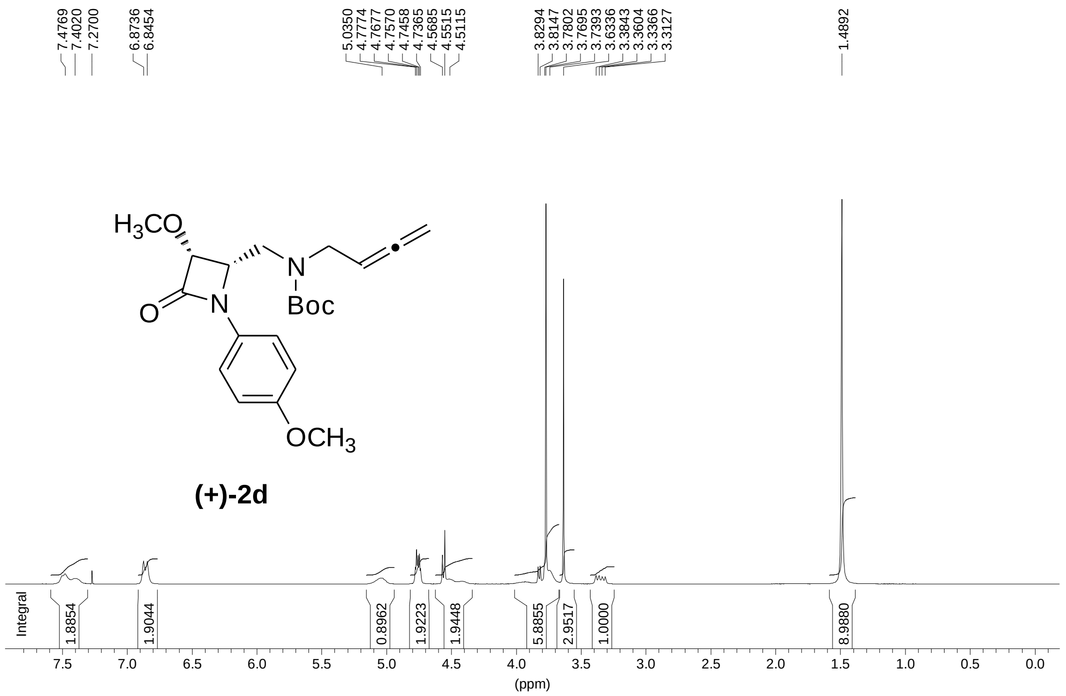
7.4769
7.4020
7.2700
6.8736
6.8454
5.0350
4.7774
4.7677
4.7570
4.7458
4.7365
4.5685
4.5515
4.5115
3.8294
3.8147
3.7802
3.7695
3.7393
3.6336
3.3843
3.3604
3.3366
3.3127
1.4892
H3CO
N
Boc
O
N
OCH3
(+)-2d
Integral
1.8854
1.9044
0.8962
1.9223
1.9448
5.8855
2.9517
1.0000
8.9880
(ppm)

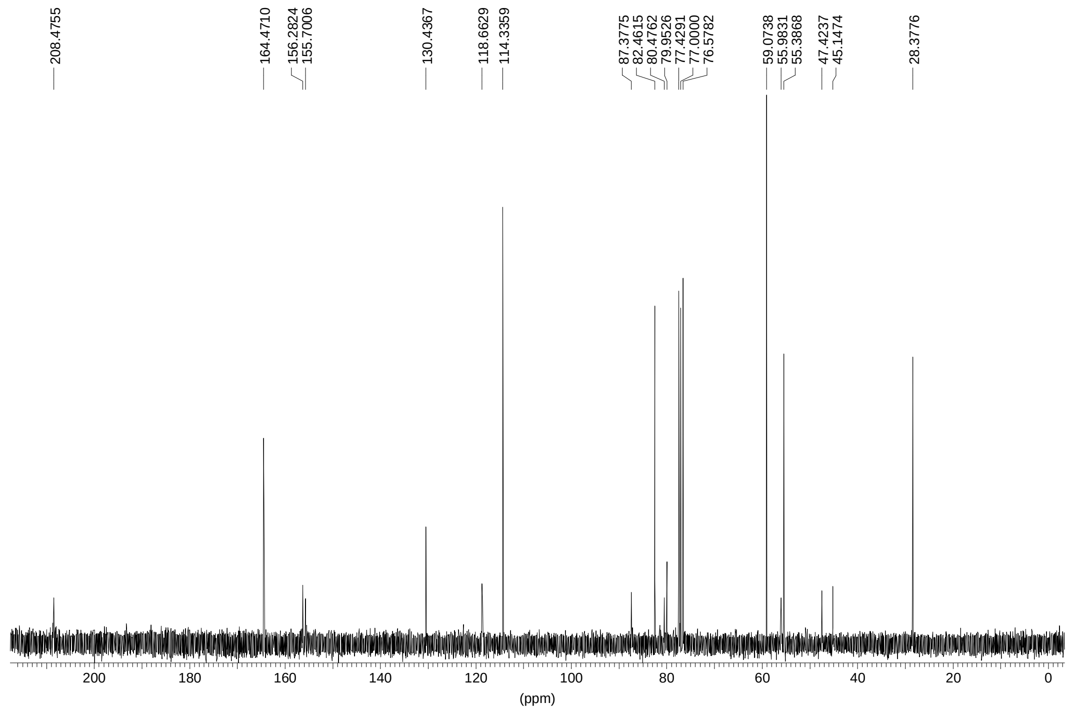
208.4755
164.4710
156.2824
155.7006
130.4367
118.6629
114.3359
87.3775
82.4615
80.4762
79.9526
77.4291
77.0000
76.5782
59.0738
55.9831
55.3868
47.4237
45.1474
28.3776
(ppm)

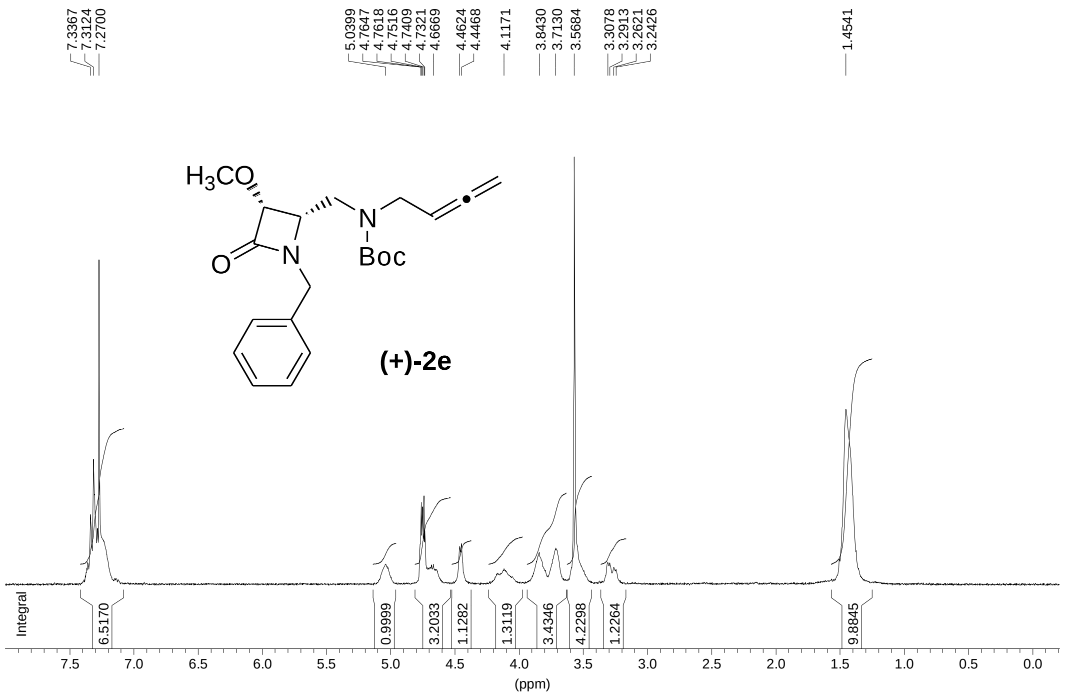

7.3367
7.3124
7.2700
5.0399
4.7647
4.7618
4.7516
4.7409
4.7321
4.6669
4.4624
4.4468
4.1171
3.8430
3.7130
3.5684
3.3078
3.2913
3.2621
3.2426
1.4541
H3CO
N
O
N
Boc
(+)-2e
Integral
6.5170
0.9999
3.2033
1.1282
1.3119
3.4346
4.2298
1.2264
9.8845
(ppm)

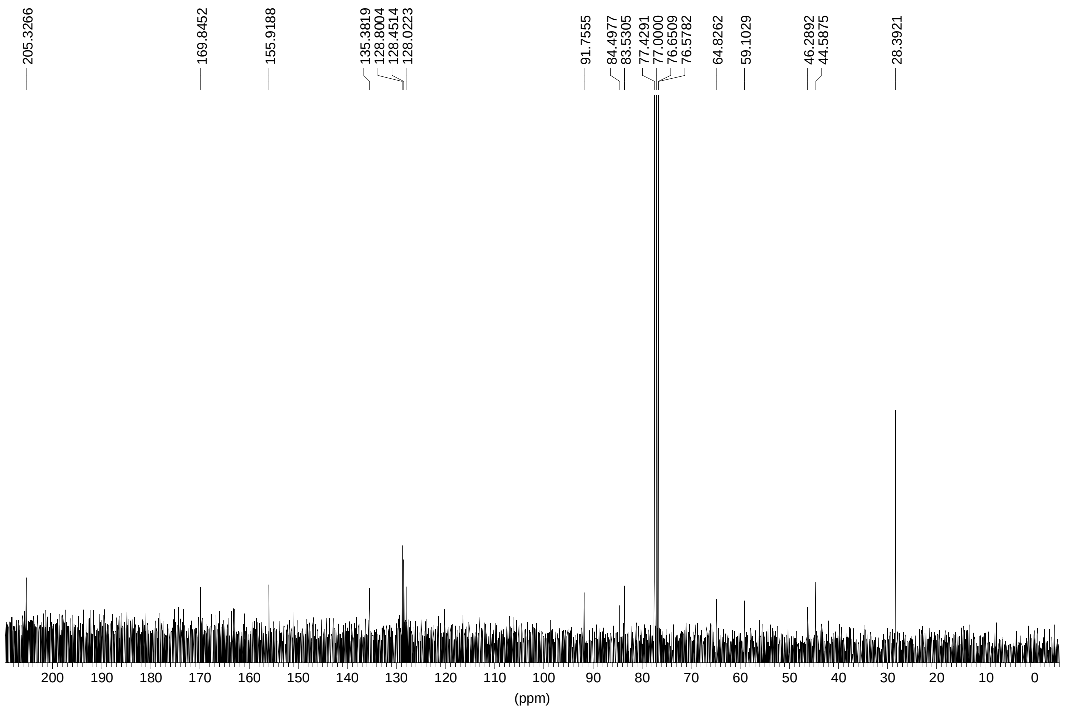

205.3266
169.8452
155.9188
135.3819
128.8004
128.4514
128.0223
91.7555
84.4977
83.5305
77.4291
77.0000
76.6509
76.5782
64.8262
59.1029
46.2892
44.5875
28.3921
(ppm)

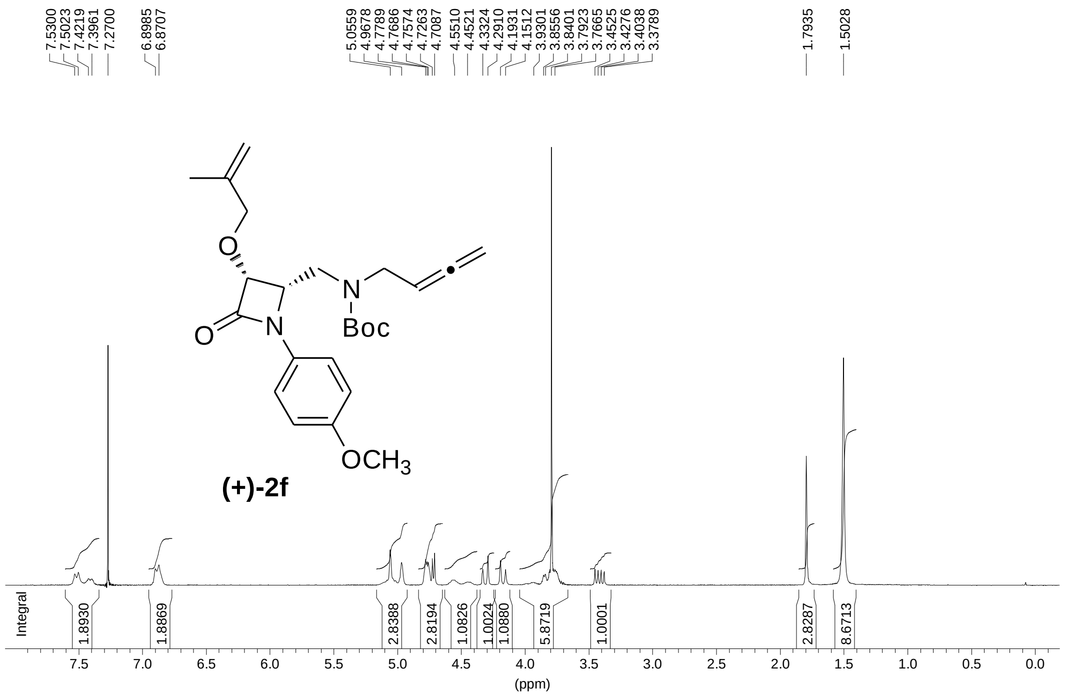
7.5300
7.5023
7.4219
7.3961
7.2700
6.8985
6.8707
5.0559
4.9678
4.7789
4.7686
4.7574
4.7263
4.7087
4.5510
4.4521
4.3324
4.2910
4.1931
4.1512
3.9301
3.8556
3.8401
3.7923
3.7665
3.4525
3.4276
3.4038
3.3789
1.7935
1.5028
O
N
Boc
O
N
OCH3
(+)-2f
Integral
1.8930
1.8869
2.8388
2.8194
1.0826
1.0024
1.0880
5.8719
1.0001
2.8287
8.6713
(ppm)

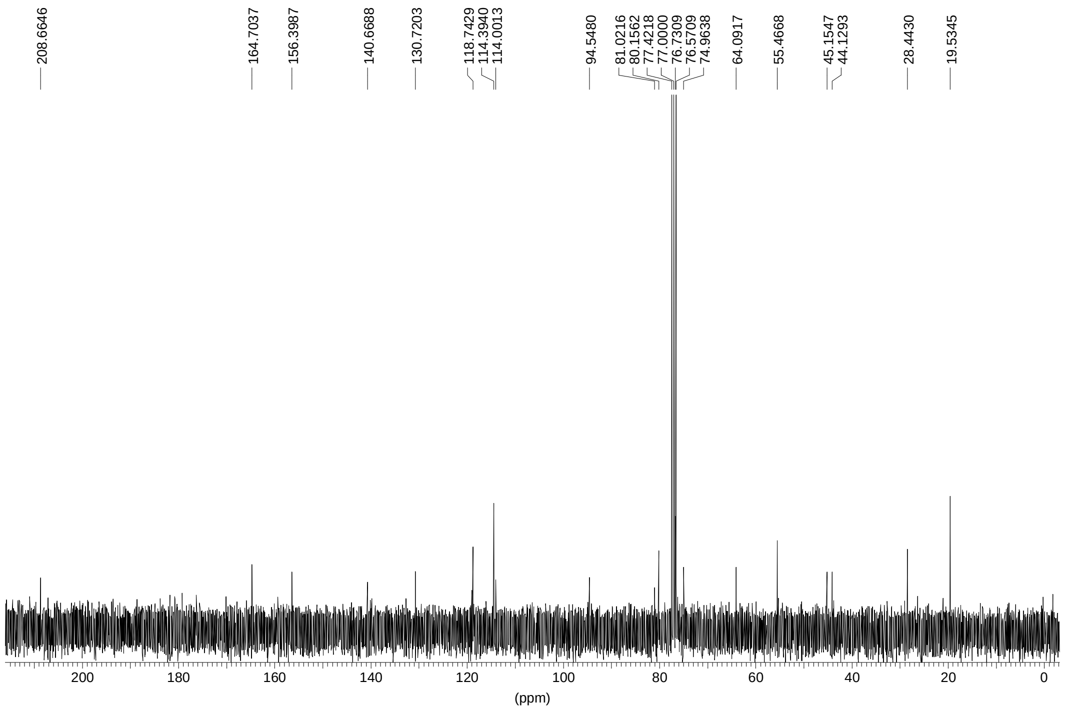
208.6646
164.7037
156.3987
140.6688
130.7203
118.7429
114.3940
114.0013
94.5480
81.0216
80.1562
77.4218
77.0000
76.7309
76.5709
74.9638
64.0917
55.4668
45.1547
44.1293
28.4430
19.5345
(ppm)

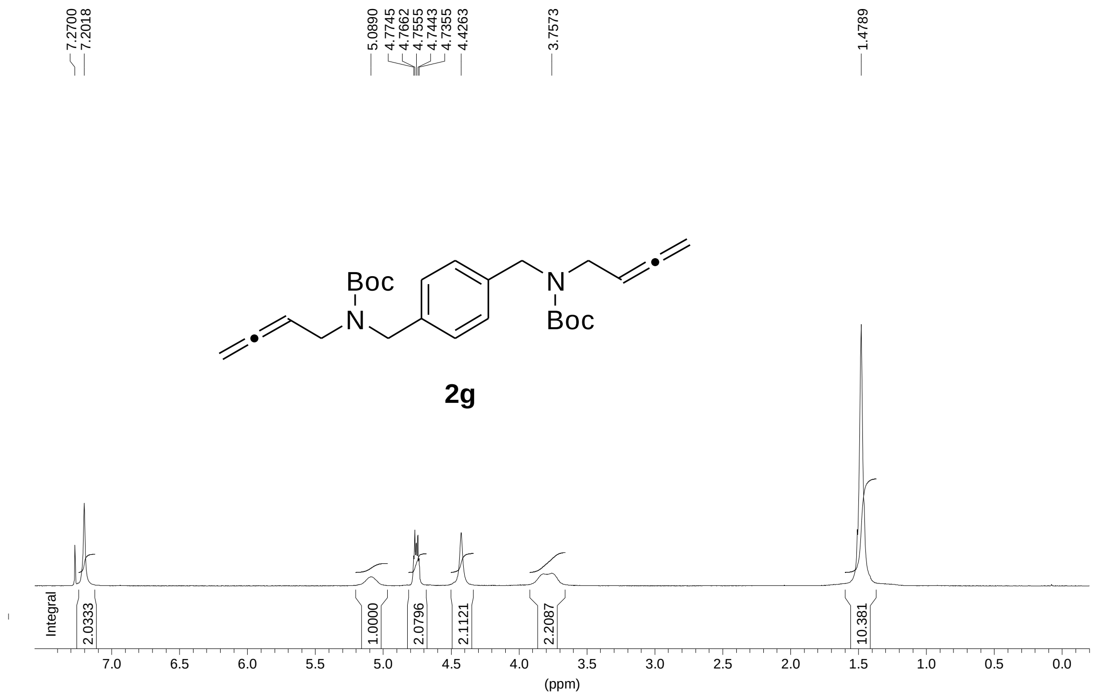
7.2700
7.2018
5.0890
4.7745
4.7662
4.7555
4.7443
4.7355
4.4263
3.7573
1.4789
Boc
N
N
Boc
2g
Integral
2.0333
1.0000
2.0796
2.1121
2.2087
10.381
(ppm)

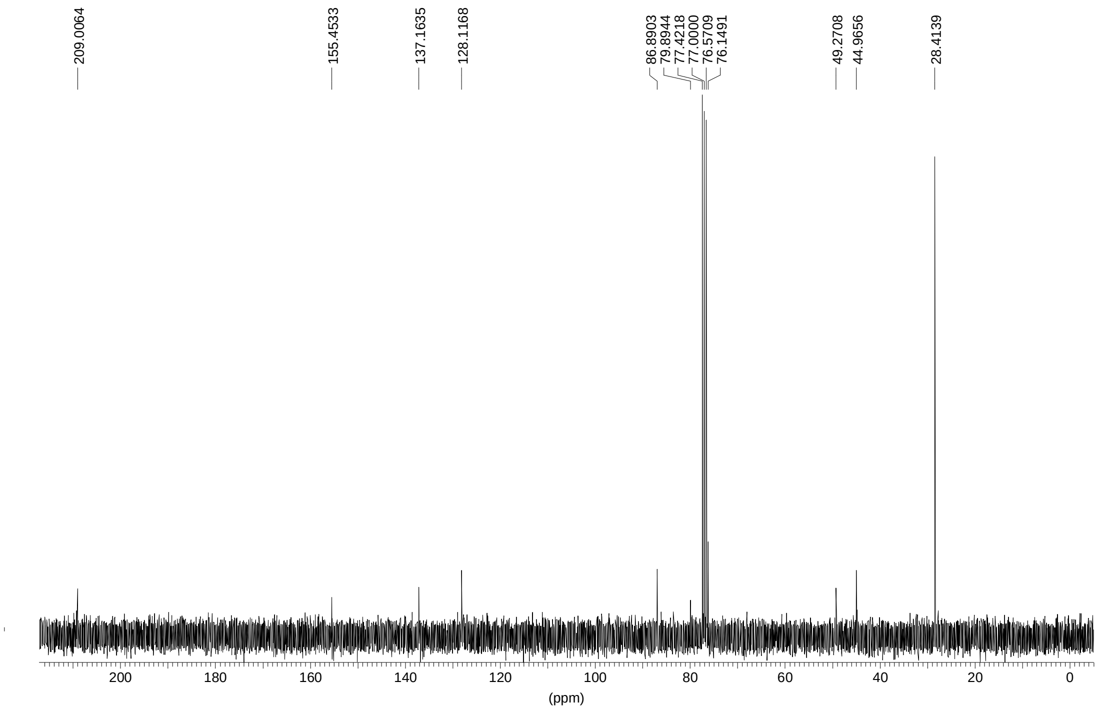
209.0064
155.4533
137.1635
128.1168
86.8903
79.8944
77.4218
77.0000
76.5709
76.1491
49.2708
44.9656
28.4139
(ppm)

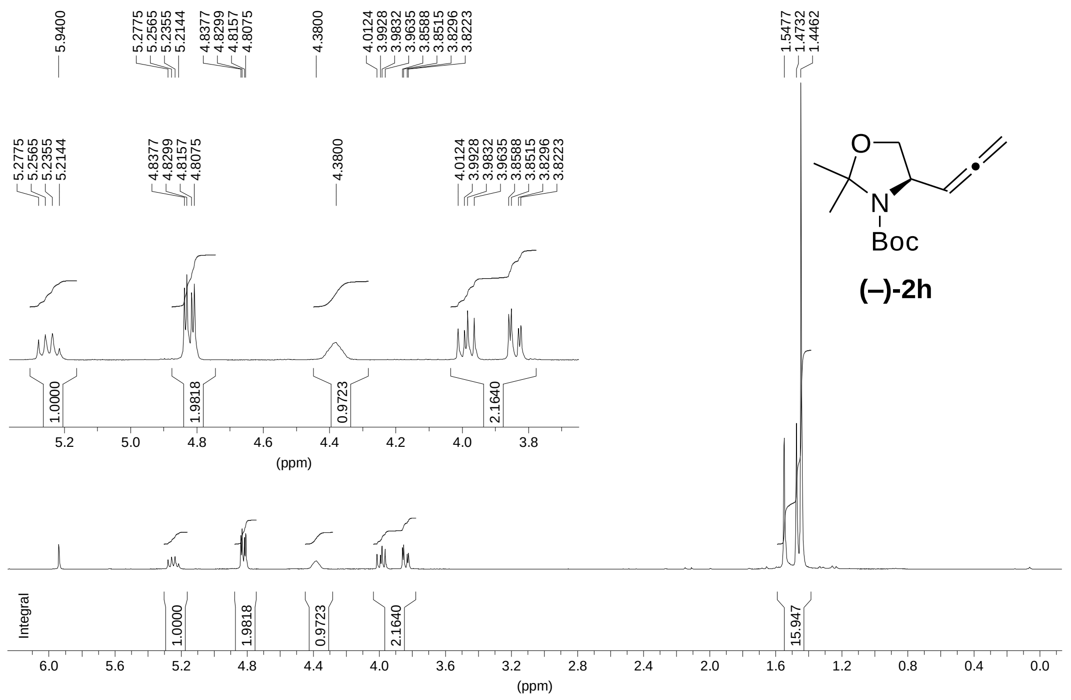
5.9400
5.2775 5.2565 5.2355 5.2144
4.8377 4.8299 4.8157 4.8075
4.3800
4.0124 3.9928 3.9832 3.9635 3.8588 3.8515 3.8296 3.8223
1.5477 1.4732 1.4462
1.0000 1.9818 0.9723 2.1640
Integral
15.947
(ppm)
Boc
(–)-2h

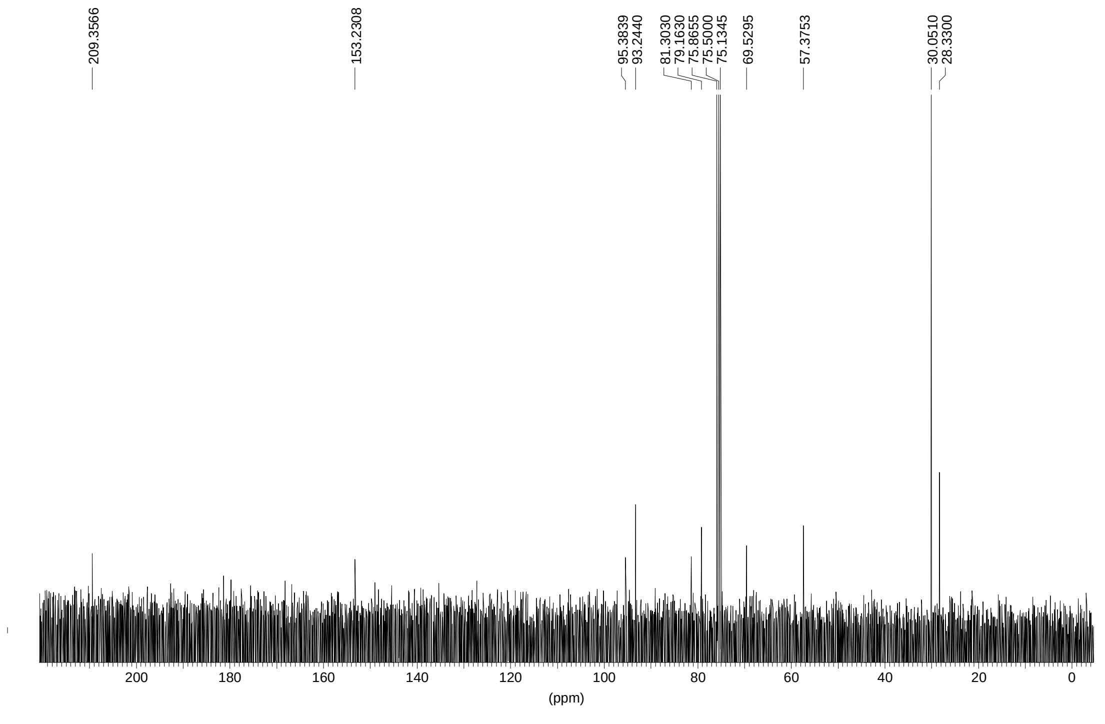
209.3566
153.2308
95.3839 93.2440
81.3030 79.1630 75.8655 75.5000 75.1345
69.5295
57.3753
30.0510 28.3300
(ppm)

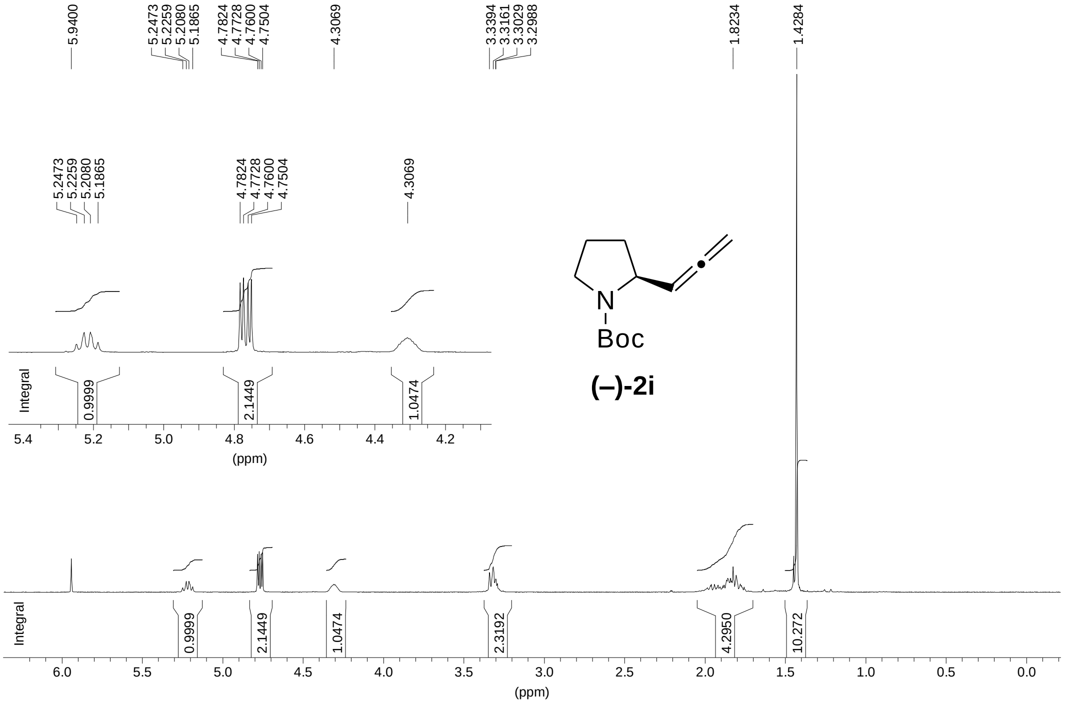
5.9400
5.2473 5.2259 5.2080 5.1865
4.7824 4.7728 4.7600 4.7504
4.3069
3.3394 3.3161 3.3029 3.2988
1.8234
1.4284
N
Boc
(–)-2i
Integral
0.9999
2.1449
1.0474
2.3192
4.2950
10.272
(ppm)

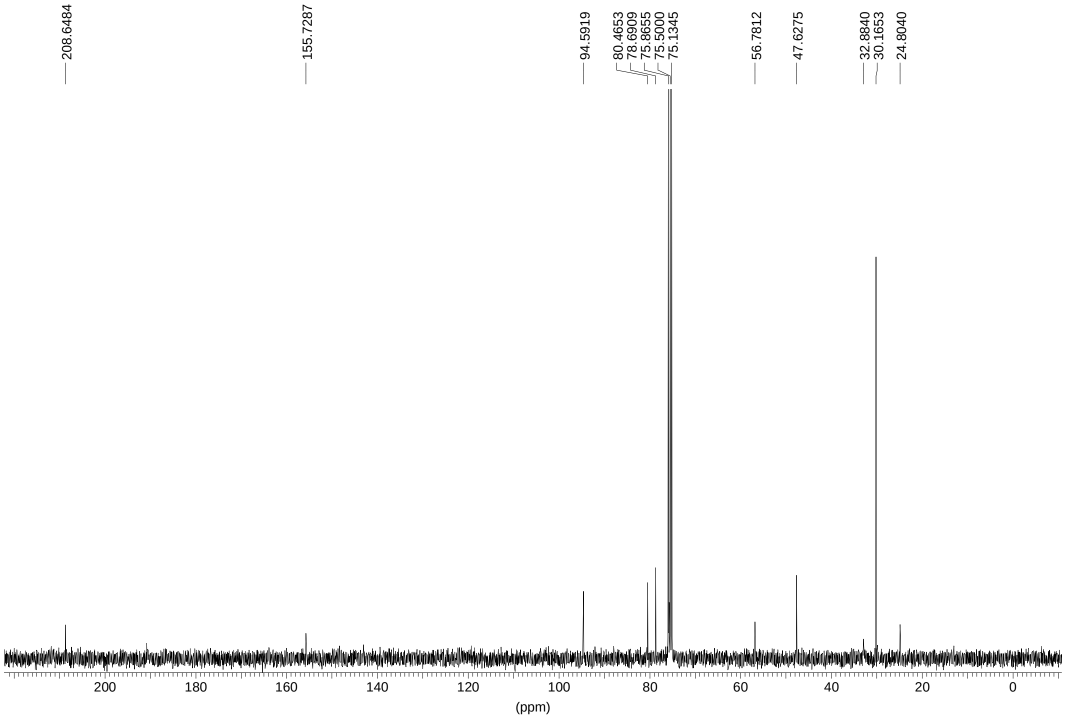
208.6484
155.7287
94.5919
80.4653 78.6909 75.8655 75.5000 75.1345
56.7812
47.6275
32.8840 30.1653 24.8040
(ppm)

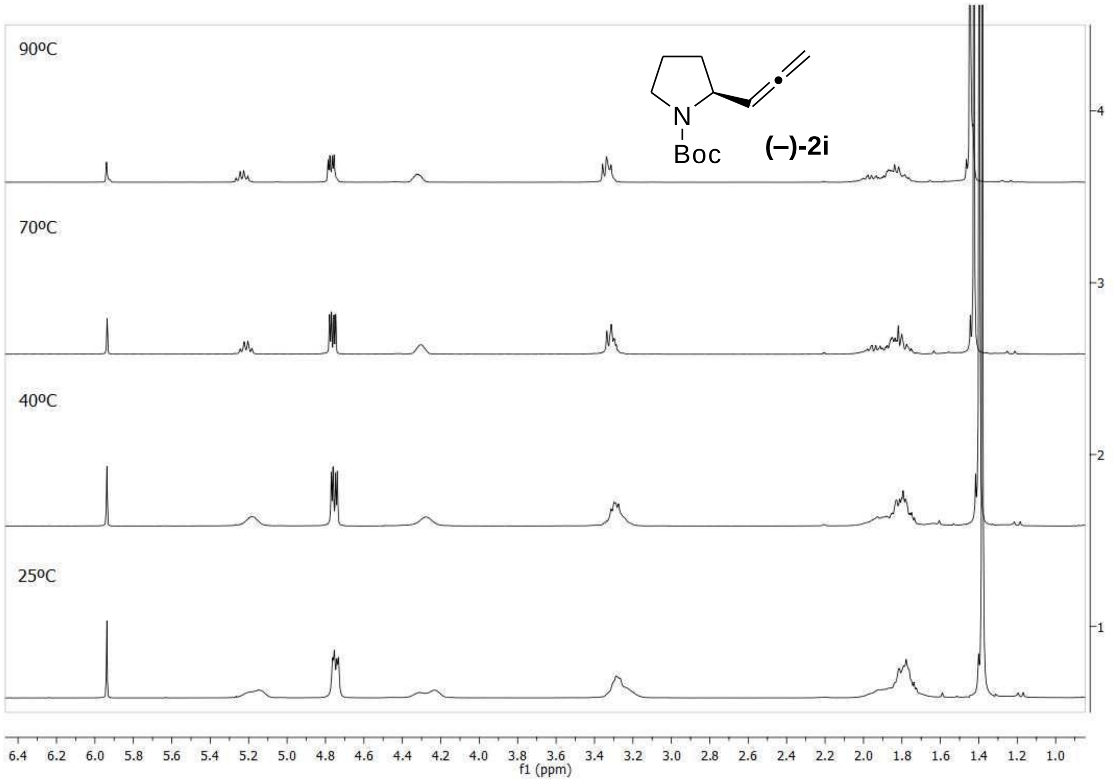
90ºC
70ºC
40ºC
25ºC
Boc
(–)-2i
f1 (ppm)

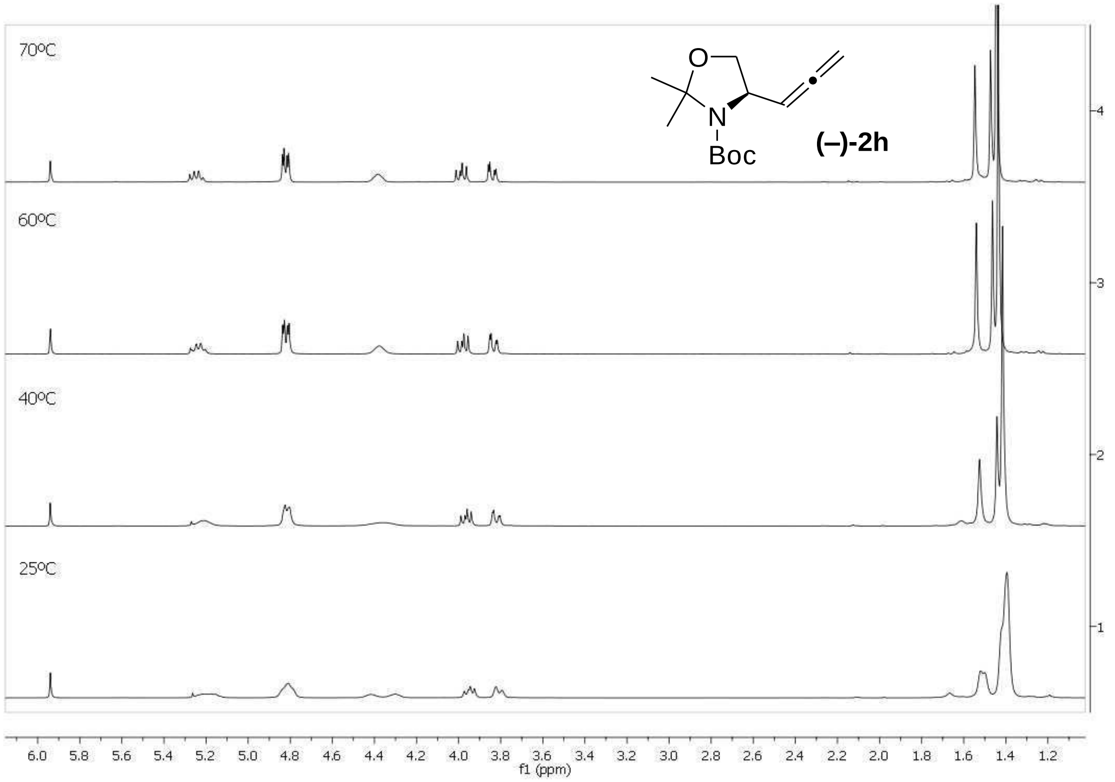
70ºC
60ºC
40ºC
25ºC
Boc
(–)-2h
f1 (ppm)

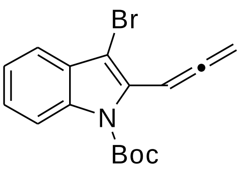
Br
N
Boc

2j

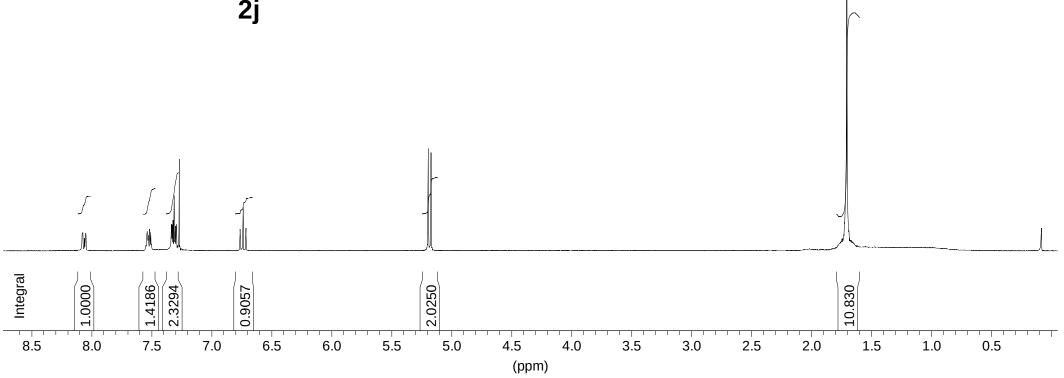
8.0734
8.0525
8.0501
8.0476
7.5368
7.5329
7.5266
7.5154
7.5081
7.5062
7.3343
7.3284
7.3202
7.3114
7.3017
7.2958
7.2905
7.2719
7.2700
6.7607
6.7373
6.7134
5.1942
5.1704
1.7044
Integral
1.0000
1.4186
2.3294
0.9057
2.0250
10.830
8.5
8.0
7.5
7.0
6.5
6.0
5.5
5.0
4.5
4.0
3.5
3.0
2.5
2.0
1.5
1.0
0.5
(ppm)

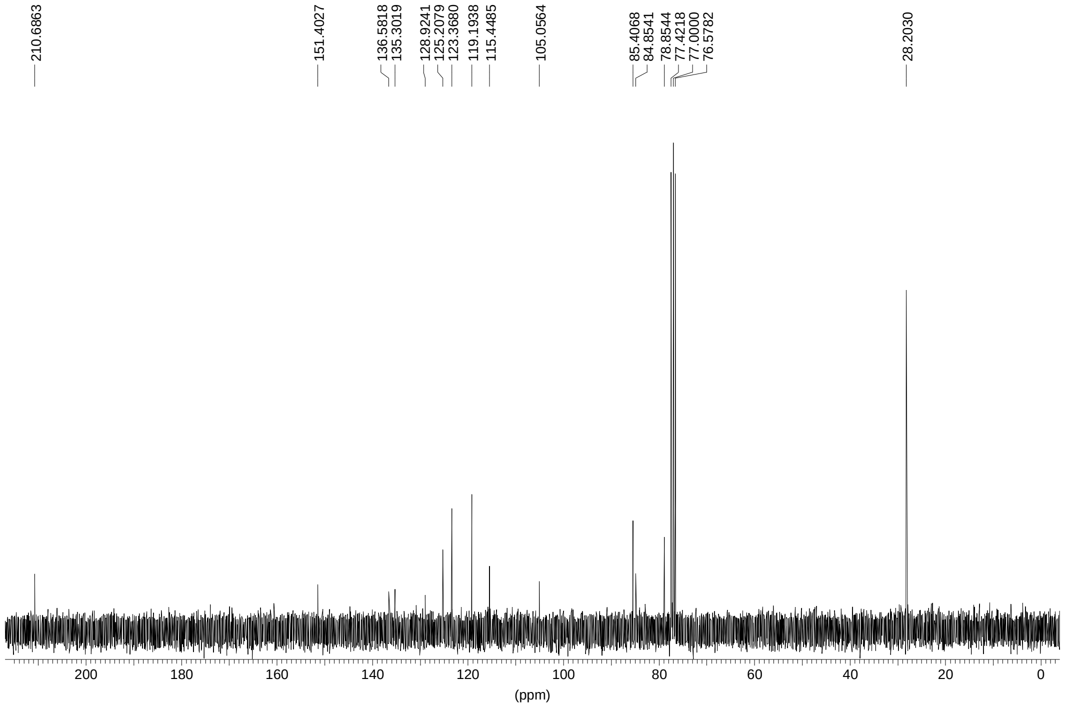
210.6863
151.4027
136.5818
135.3019
128.9241
125.2079
123.3680
119.1938
115.4485
105.0564
85.4068
84.8541
78.8544
77.4218
77.0000
76.5782
28.2030
200
180
160
140
120
100
80
60
40
20
0
(ppm)

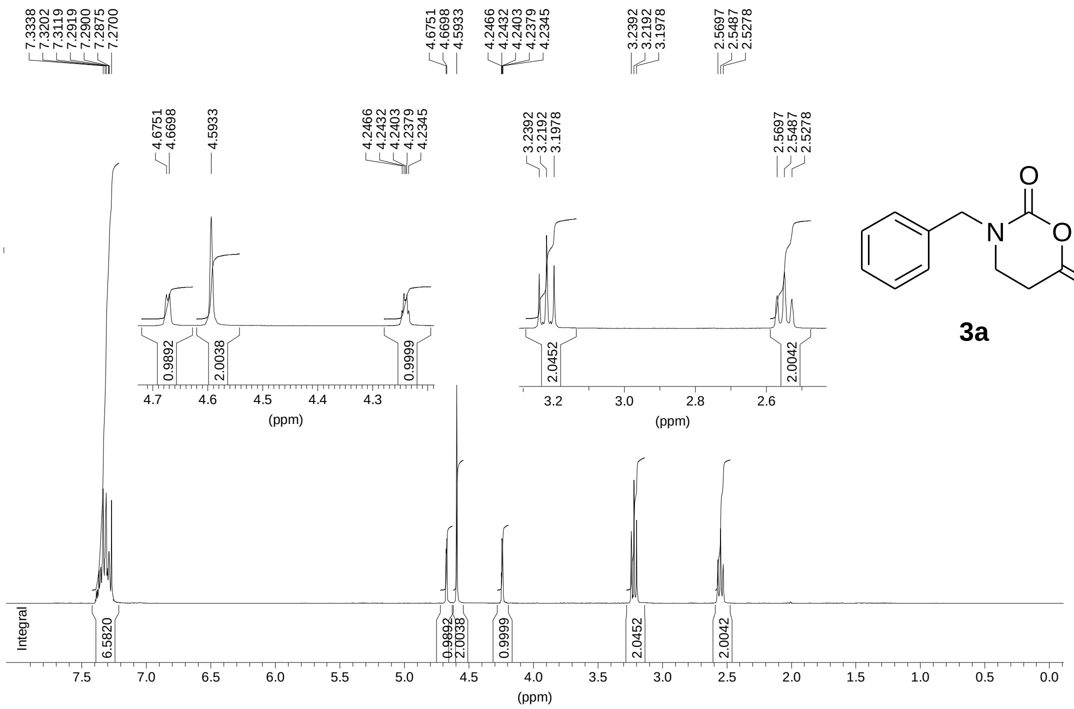

3a

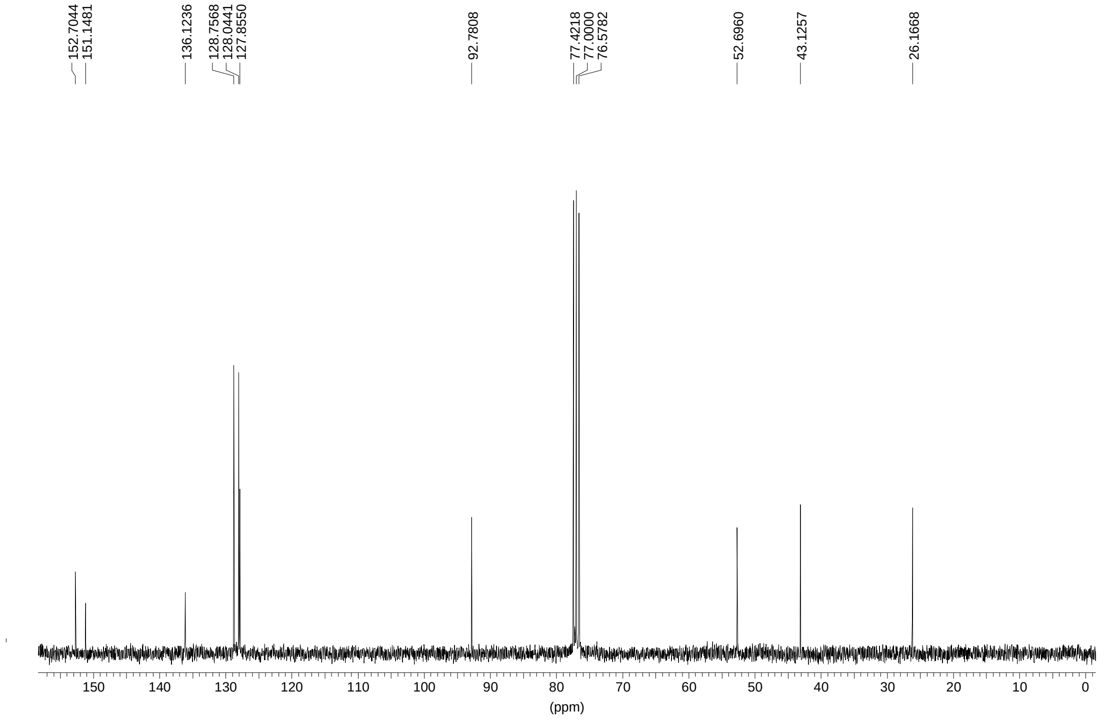

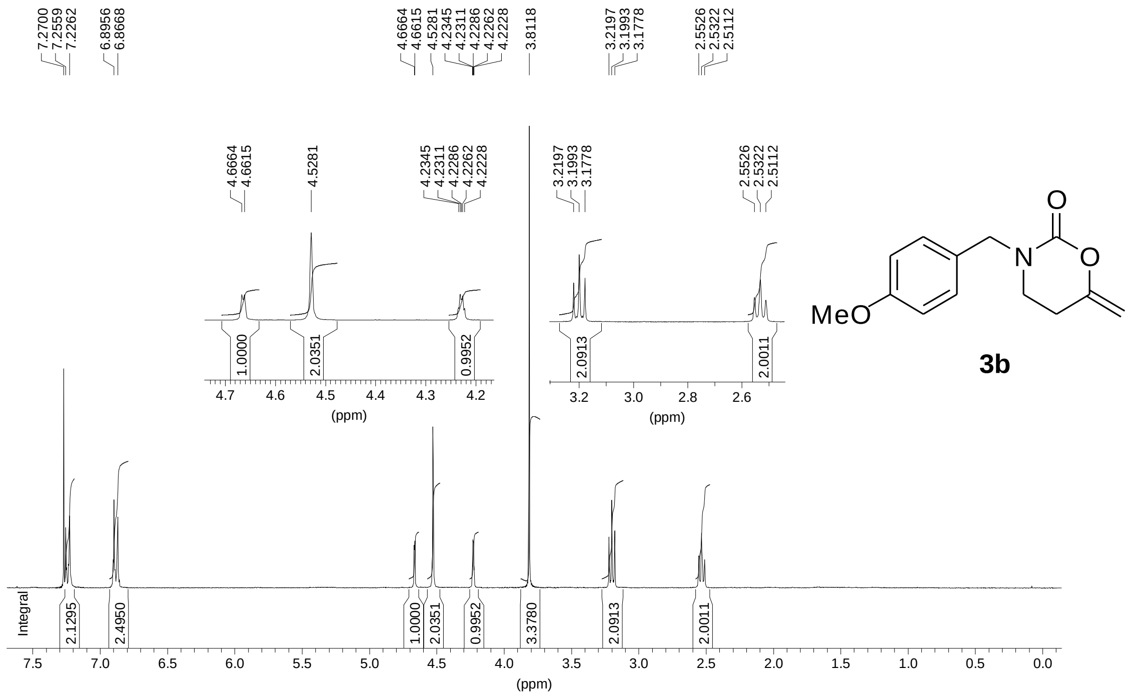
7.2700
7.2559
7.2262
6.8956
6.8668
4.6664
4.6615
4.5281
4.2345
4.2311
4.2286
4.2262
4.2228
3.8118
3.2197
3.1993
3.1778
2.5526
2.5322
2.5112
Integral
2.1295
2.4950
1.0000
2.0351
0.9952
3.3780
2.0913
2.0011
(ppm)
MeO
N
O
O

3b

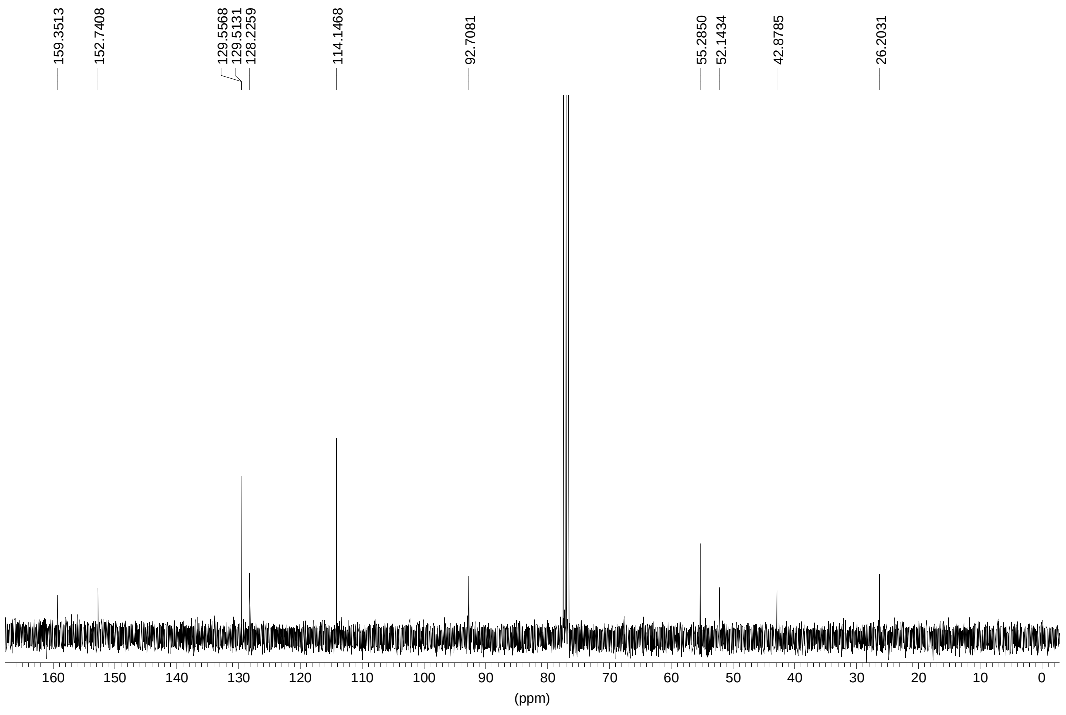
159.3513
152.7408
129.5568
129.5131
128.2259
114.1468
92.7081
55.2850
52.1434
42.8785
26.2031
(ppm)

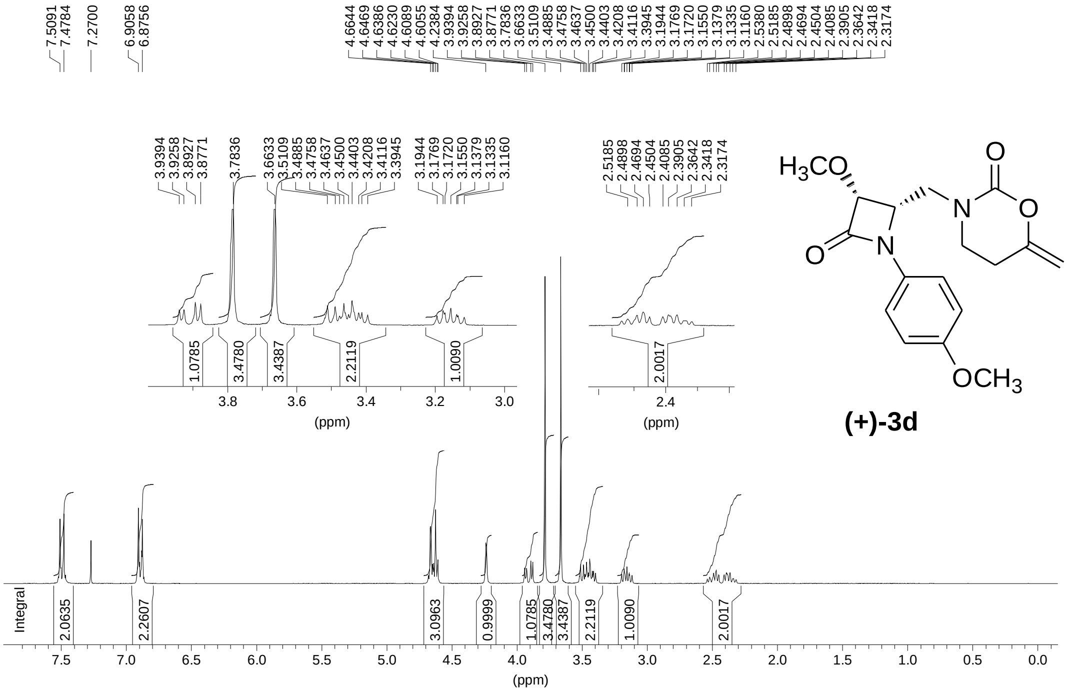

(+)-3d

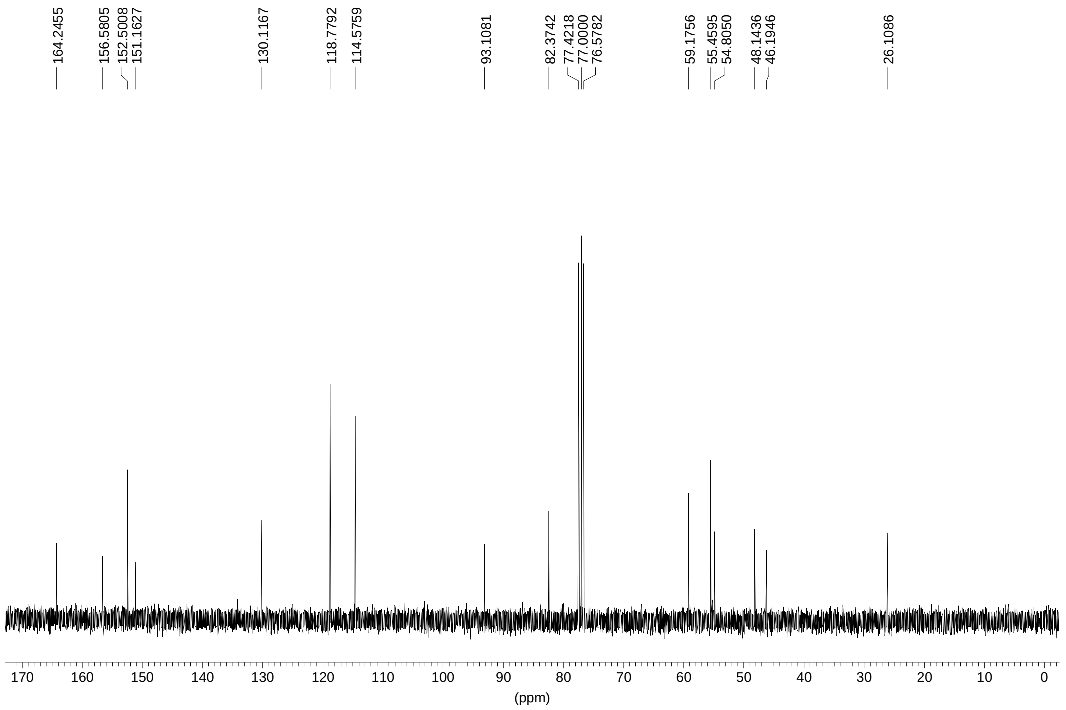

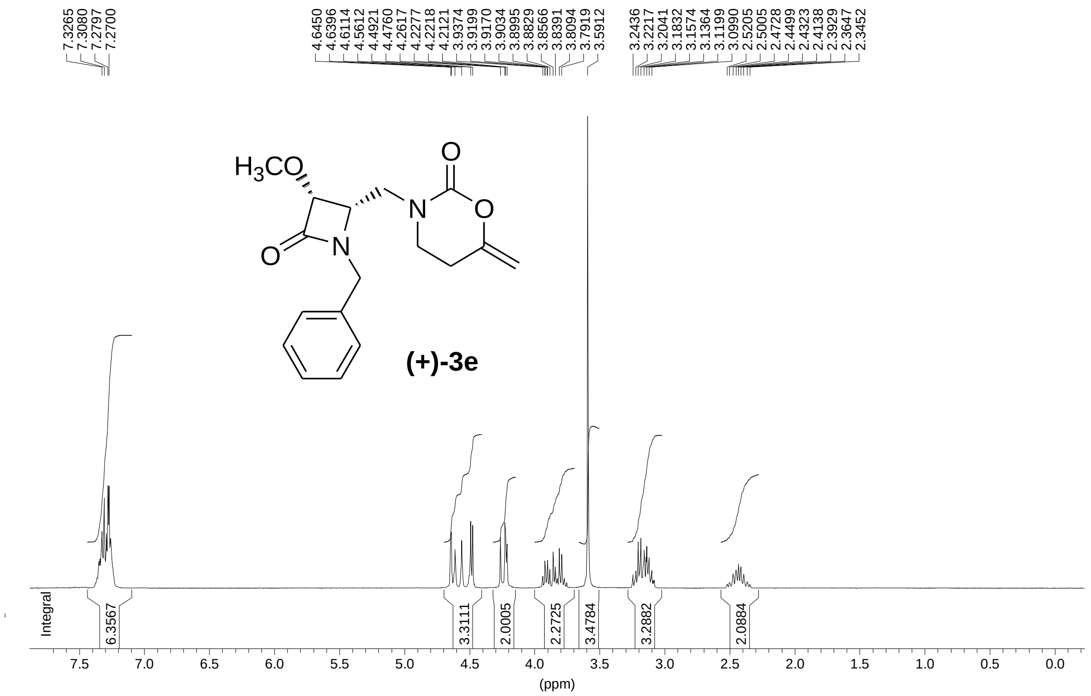
7.3265
7.3080
7.2797
7.2700
4.6450
4.6396
4.6114
4.5612
4.4921
4.4760
4.2617
4.2277
4.2218
4.2121
3.9374
3.9199
3.9170
3.9034
3.8995
3.8829
3.8566
3.8391
3.8094
3.7919
3.5912
3.2436
3.2217
3.2041
3.1832
3.1574
3.1364
3.1199
3.0990
2.5205
2.5005
2.4728
2.4499
2.4323
2.4138
2.3929
2.3647
2.3452
H_3CO
O
N
O
O
N
(+)-3e
Integral
6.3567
3.3111
2.0005
2.2725
3.4784
3.2882
2.0884
(ppm)

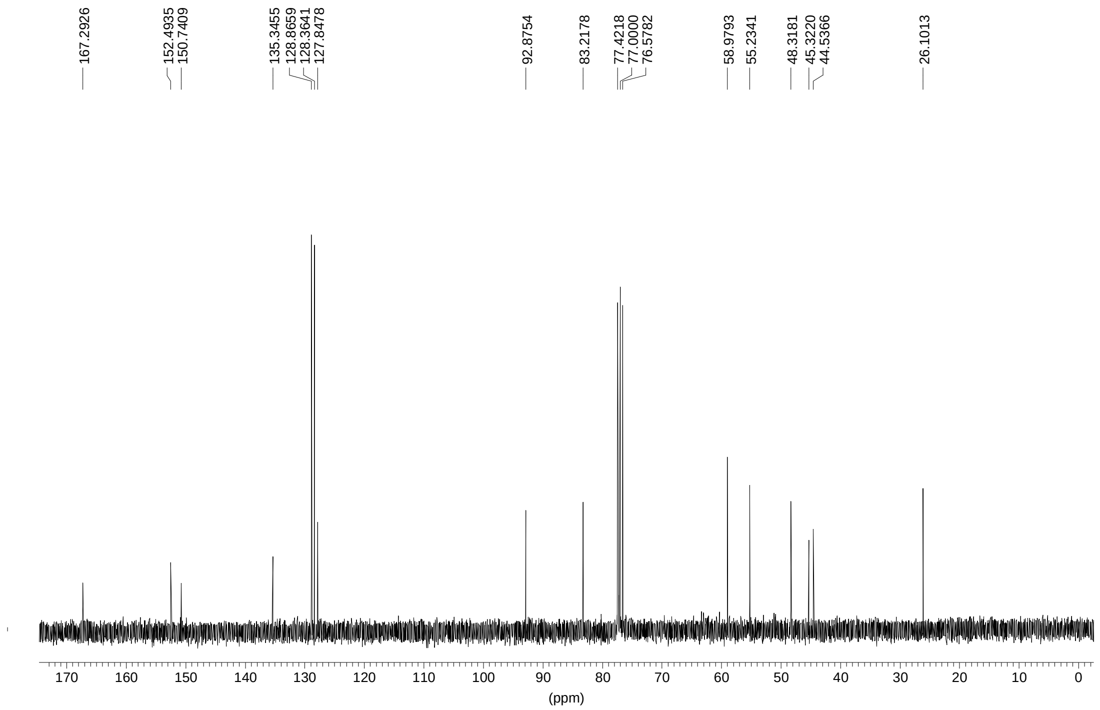
167.2926
152.4935
150.7409
135.3455
128.8659
128.3641
127.8478
92.8754
83.2178
77.4218
77.0000
76.5782
58.9793
55.2341
48.3181
45.3220
44.5366
26.1013
(ppm)

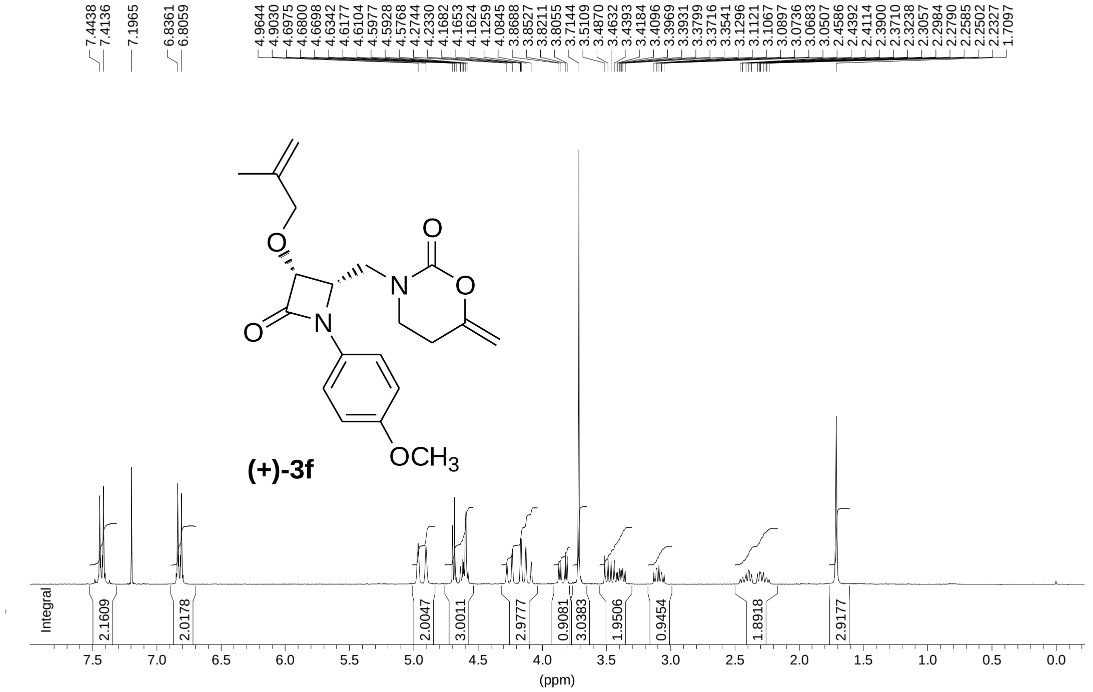
7.4438
7.4136
7.1965
6.8361
6.8059
4.9644
4.9030
4.6975
4.6800
4.6698
4.6342
4.6177
4.6104
4.5977
4.5928
4.5768
4.2744
4.2330
4.1682
4.1653
4.1624
4.1259
4.0845
3.8688
3.8527
3.8211
3.8055
3.7144
3.5109
3.4870
3.4632
3.4393
3.4184
3.4096
3.3969
3.3931
3.3799
3.3716
3.3541
3.1296
3.1121
3.1067
3.0897
3.0736
3.0683
3.0507
2.4586
2.4392
2.4114
2.3900
2.3710
2.3238
2.3057
2.2984
2.2790
2.2585
2.2502
2.2327
1.7097
O
O
N
O
O
N
OCH3
(+)-3f
Integral
2.1609
2.0178
2.0047
3.0011
2.9777
0.9081
3.0383
1.9506
0.9454
1.8918
2.9177
7.5 7.0 6.5 6.0 5.5 5.0 4.5 4.0 3.5 3.0 2.5 2.0 1.5 1.0 0.5 0.0
(ppm)

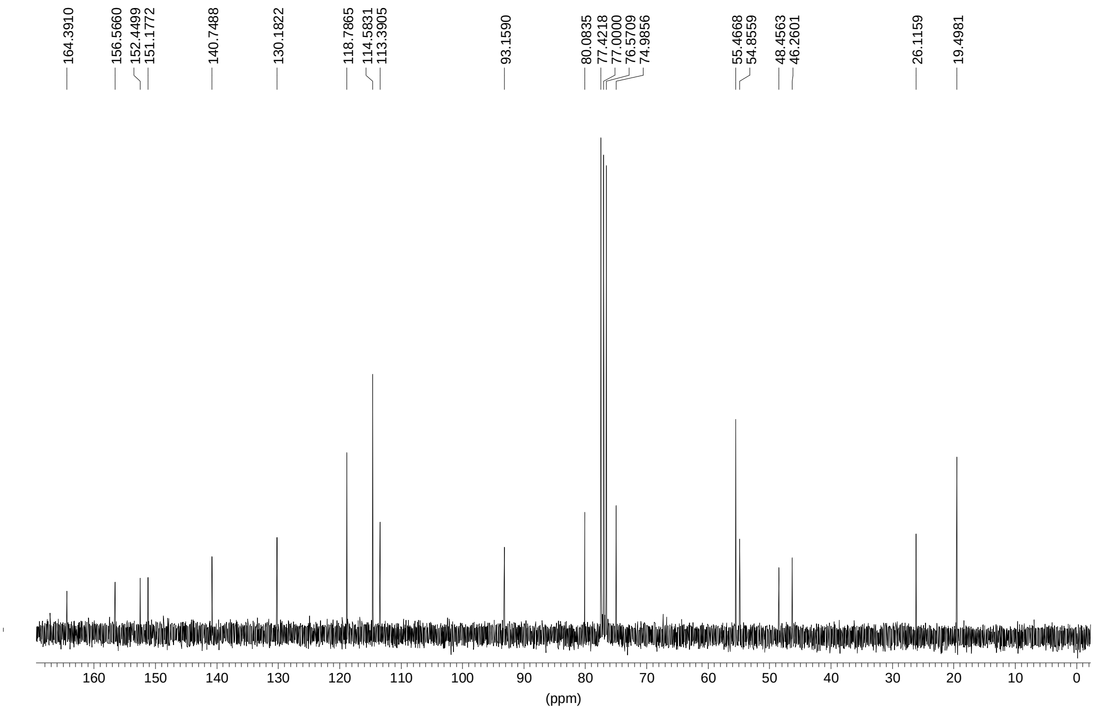
164.3910
156.5660
152.4499
151.1772
140.7488
130.1822
118.7865
114.5831
113.3905
93.1590
80.0835
77.4218
77.0000
76.5709
74.9856
55.4668
54.8559
48.4563
46.2601
26.1159
19.4981
160 150 140 130 120 110 100 90 80 70 60 50 40 30 20 10 0
(ppm)

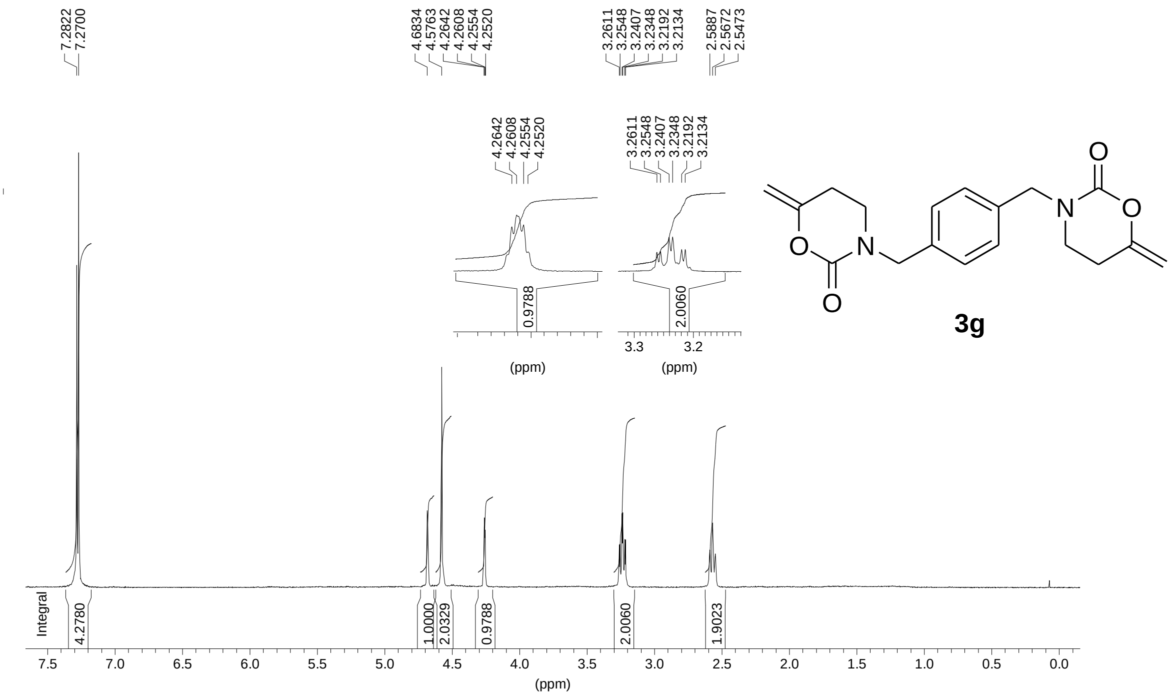
7.2822
7.2700
4.6834
4.5763
4.2642
4.2608
4.2554
4.2520
3.2611
3.2548
3.2407
3.2348
3.2192
3.2134
2.5887
2.5672
2.5473
0.9788
2.0060
3.3
3.2
(ppm)
Integral
4.2780
1.0000
2.0329
0.9788
2.0060
1.9023
7.5
7.0
6.5
6.0
5.5
5.0
4.5
4.0
3.5
3.0
2.5
2.0
1.5
1.0
0.5
0.0
(ppm)

3g

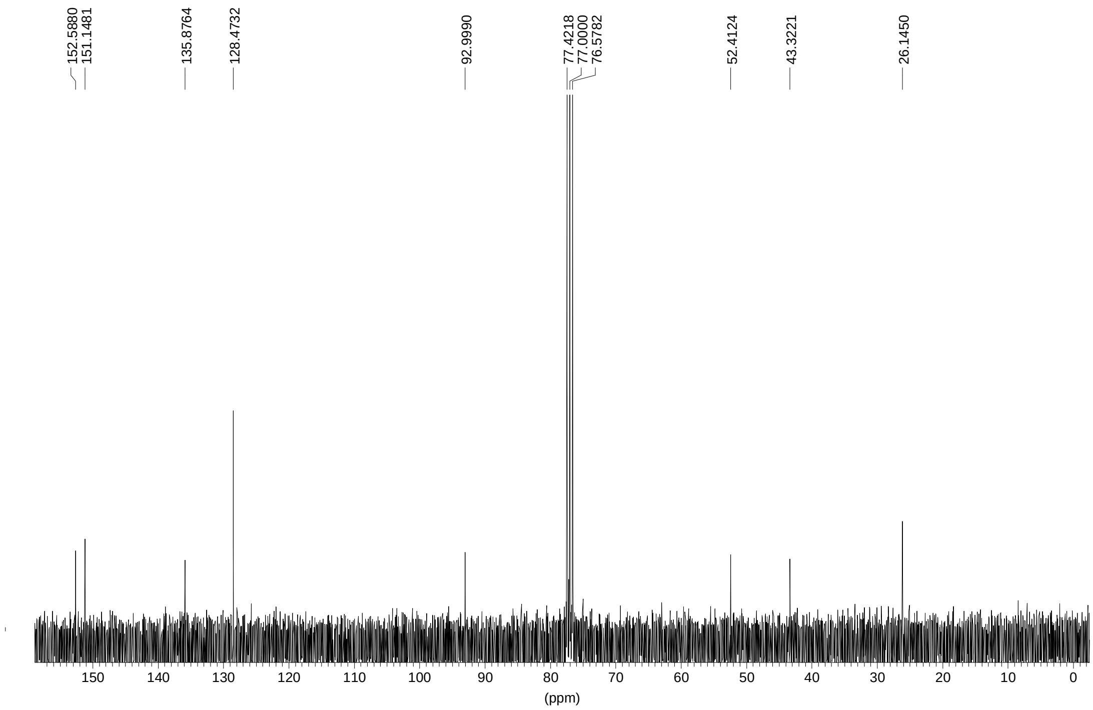
152.5880
151.1481
135.8764
128.4732
92.9990
77.4218
77.0000
76.5782
52.4124
43.3221
26.1450
150
140
130
120
110
100
90
80
70
60
50
40
30
20
10
0
(ppm)

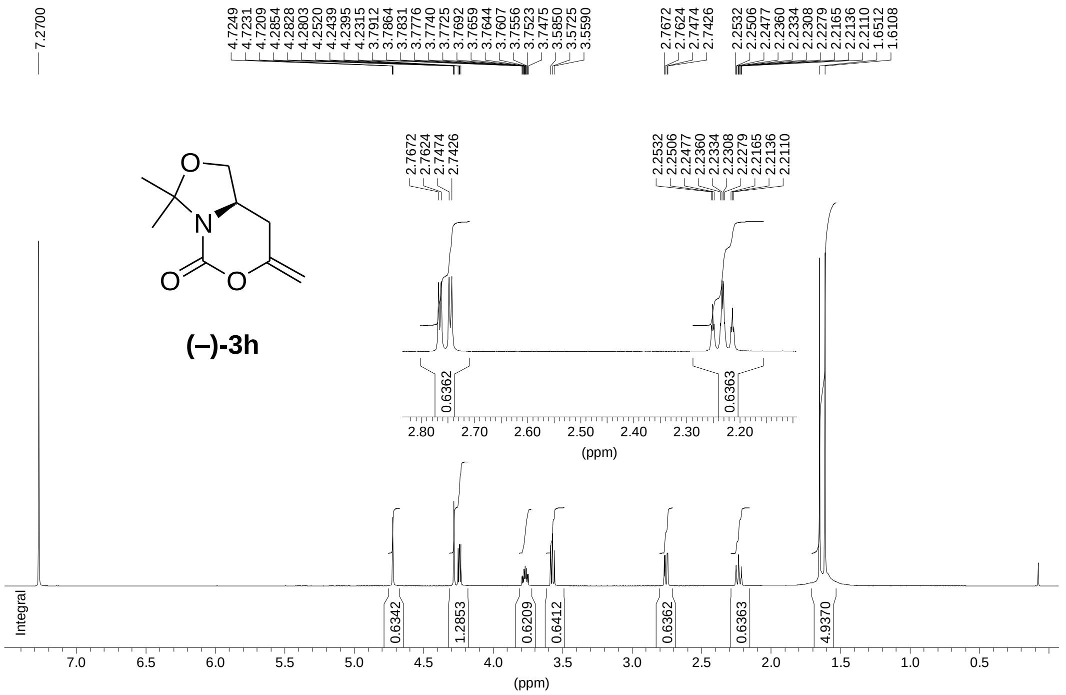
(–)-3h
Integral
(ppm)

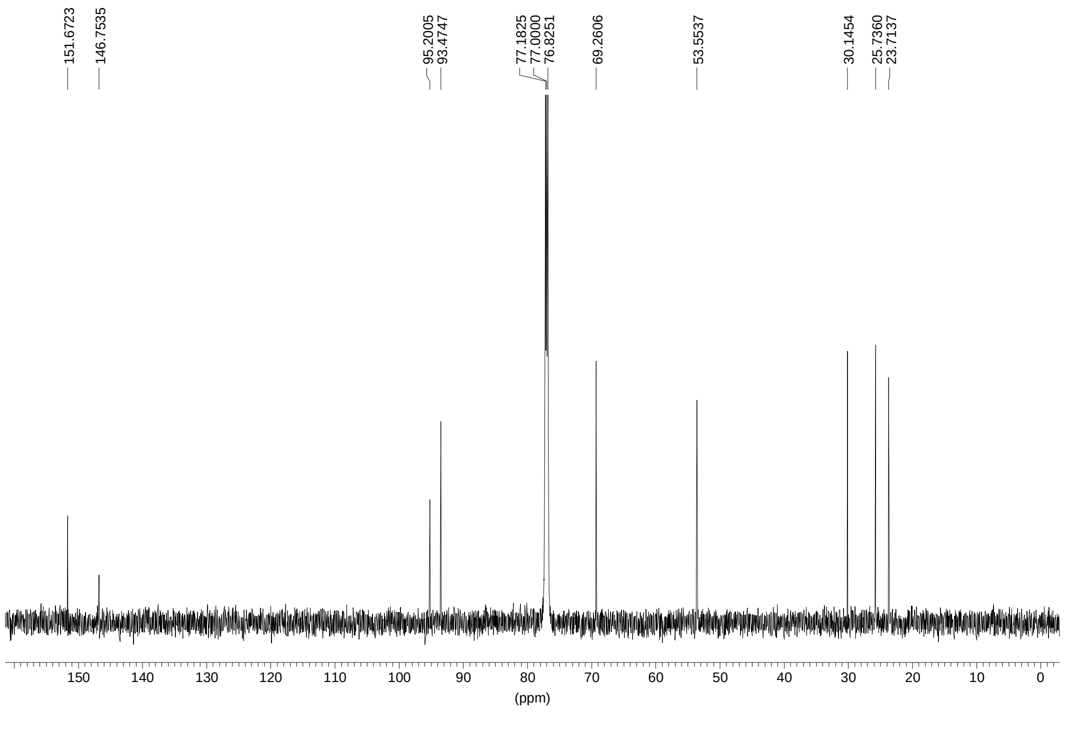
151.6723
146.7535
95.2005
93.4747
77.1825
77.0000
76.8251
69.2606
53.5537
30.1454
25.7360
23.7137
(ppm)

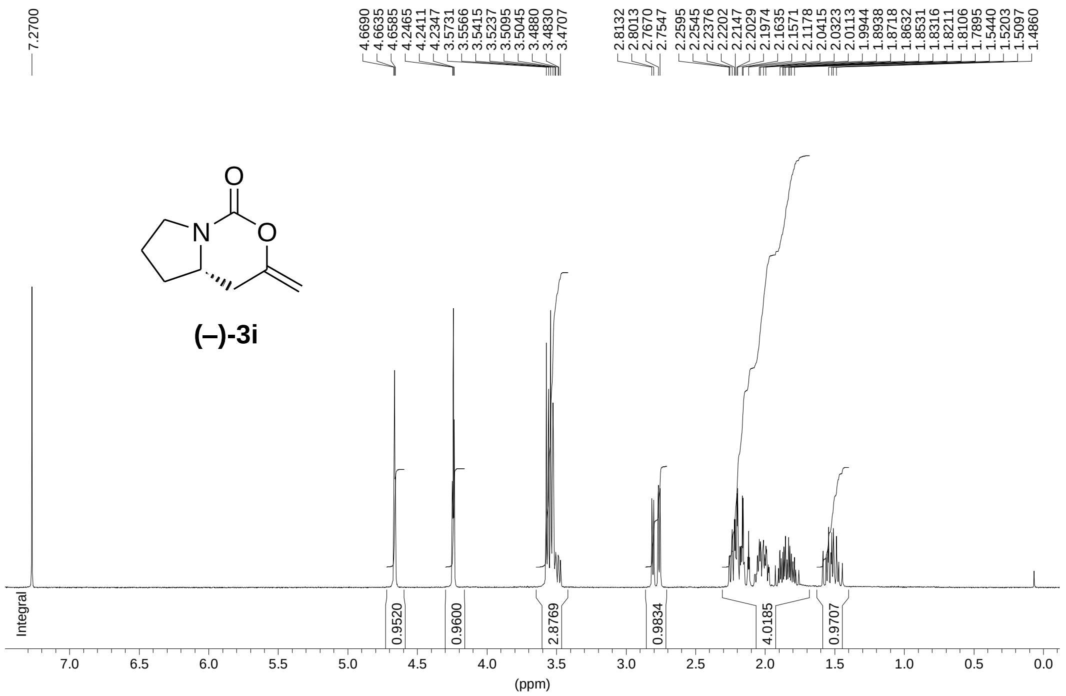
7.2700
4.6690
4.6635
4.6585
4.2465
4.2411
4.2347
3.5731
3.5566
3.5415
3.5237
3.5095
3.5045
3.4880
3.4830
3.4707
2.8132
2.8013
2.7670
2.7547
2.2595
2.2545
2.2376
2.2202
2.2147
2.2029
2.1974
2.1635
2.1571
2.1178
2.0415
2.0323
2.0113
1.9944
1.8938
1.8718
1.8632
1.8531
1.8316
1.8211
1.8106
1.7895
1.5440
1.5203
1.5097
1.4860
(–)-3i
Integral
0.9520
0.9600
2.8769
0.9834
4.0185
0.9707
(ppm)

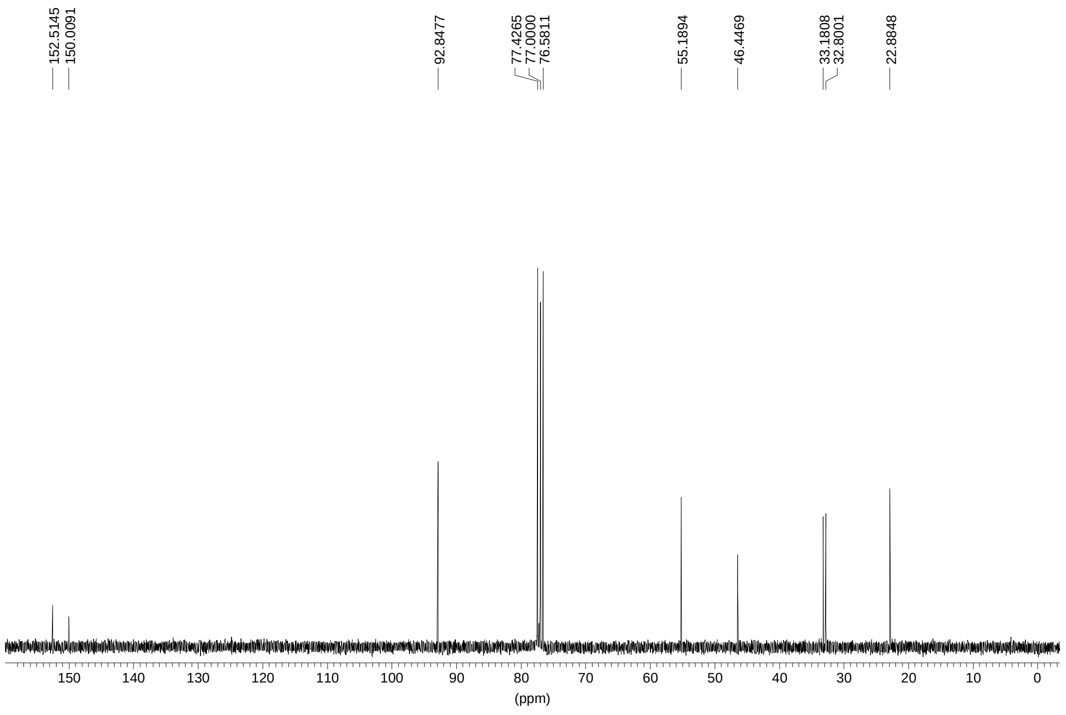
152.5145
150.0091
92.8477
77.4265
77.0000
76.5811
55.1894
46.4469
33.1808
32.8001
22.8848
(ppm)

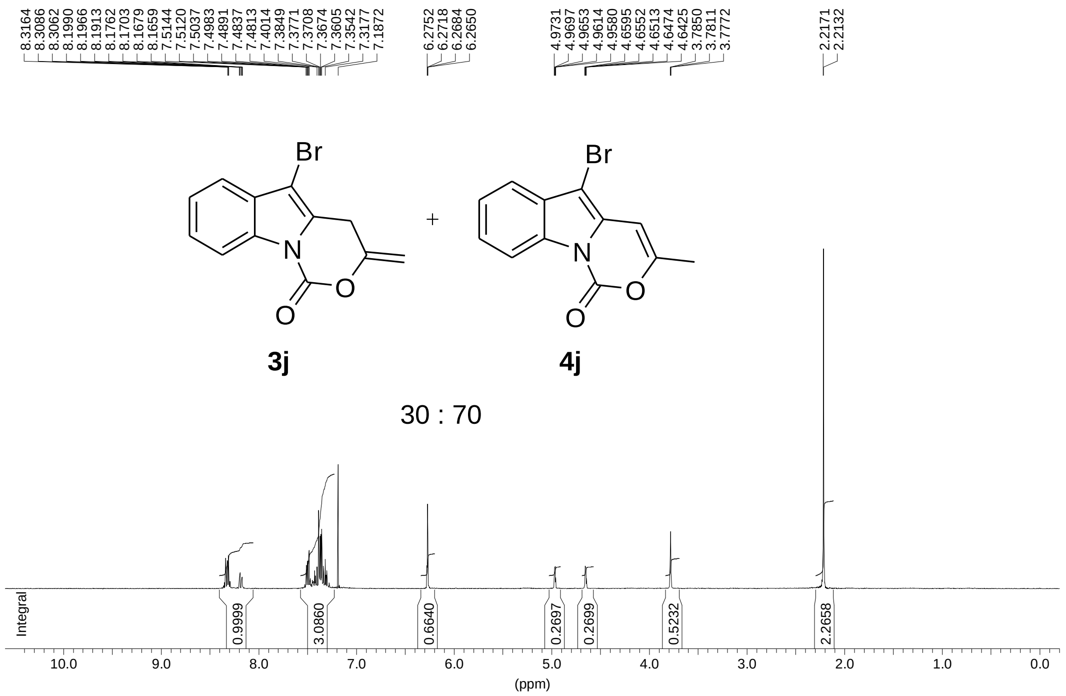
8.3164 8.3086 8.3062 8.1990 8.1966 8.1913 8.1762 8.1703 8.1679 8.1659 7.5144 7.5120 7.5037 7.4983 7.4891 7.4837 7.4813 7.4014 7.3849 7.3771 7.3708 7.3674 7.3605 7.3542 7.3177 7.1872
6.2752 6.2718 6.2684 6.2650
4.9731 4.9697 4.9653 4.9614 4.9580 4.6595 4.6552 4.6513 4.6474 4.6425 3.7850 3.7811 3.7772
2.2171 2.2132
Br
N
O
3j
4j
30 : 70
Integral
0.9999
3.0860
0.6640
0.2697
0.2699
0.5232
2.2658
10.0 9.0 8.0 7.0 6.0 5.0 4.0 3.0 2.0 1.0 0.0
(ppm)

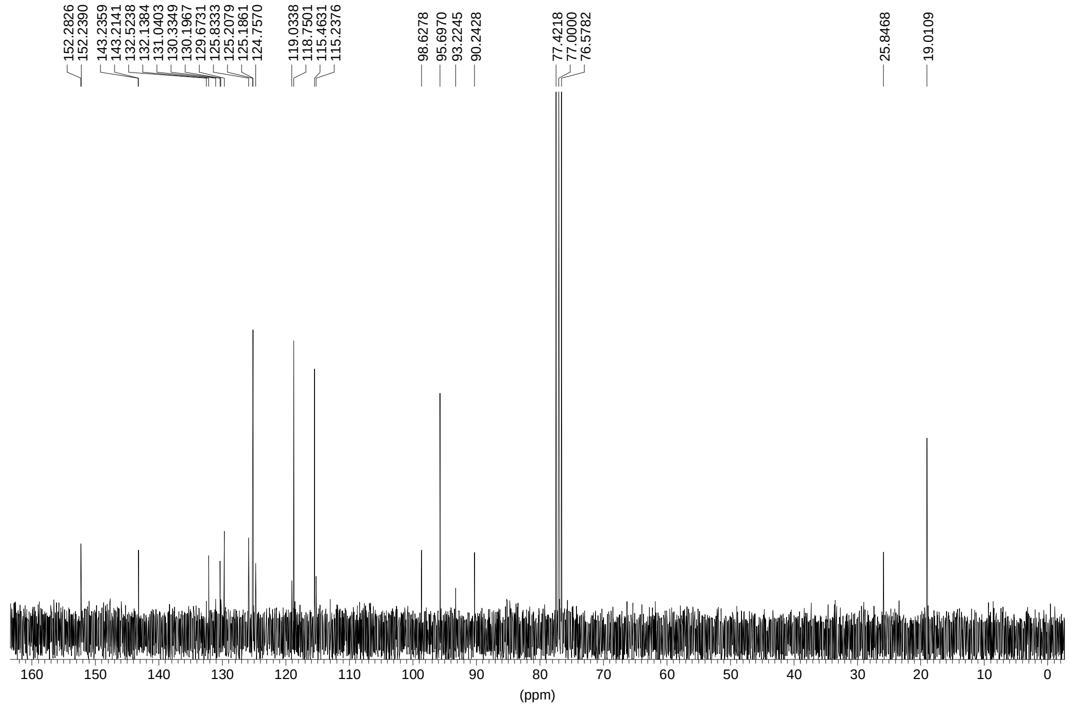
152.2826 152.2390 143.2359 143.2141 132.5238 132.1384 131.0403 130.3349 130.1967 129.6731 125.8333 125.2079 125.1861 124.7570
119.0338 118.7501 115.4631 115.2376
98.6278 95.6970 93.2245 90.2428
77.4218 77.0000 76.5782
25.8468
19.0109
160 150 140 130 120 110 100 90 80 70 60 50 40 30 20 10 0
(ppm)

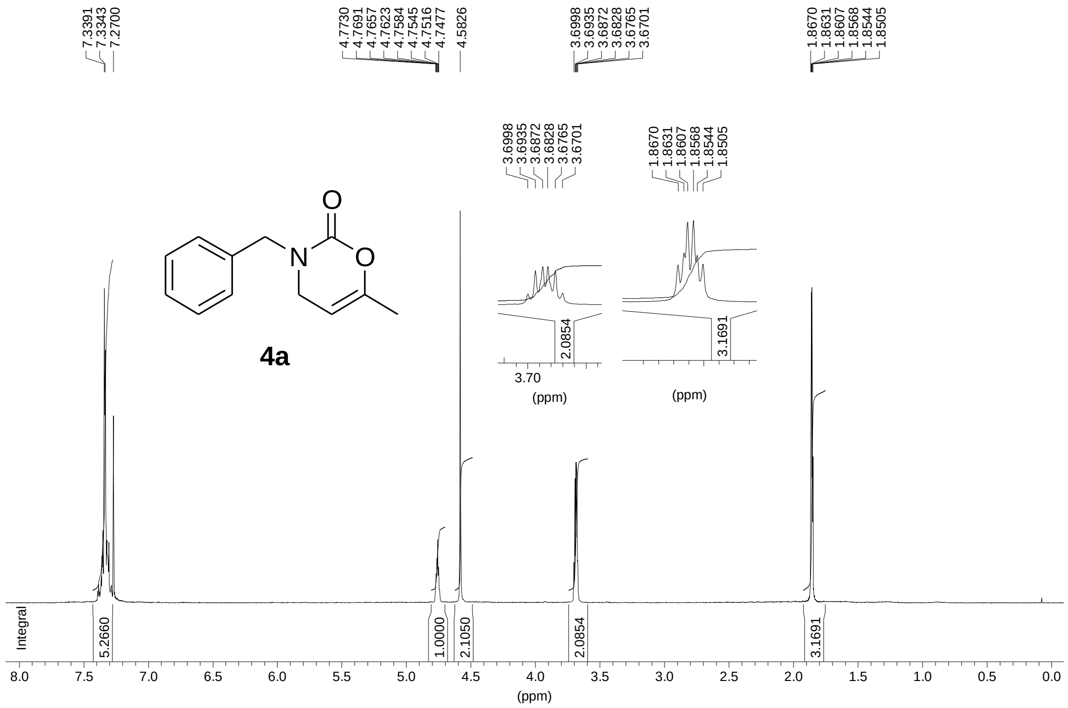
7.3391
7.3343
7.2700
4.7730
4.7691
4.7657
4.7623
4.7584
4.7545
4.7516
4.7477
4.5826
3.6998
3.6935
3.6872
3.6828
3.6765
3.6701
1.8670
1.8631
1.8607
1.8568
1.8544
1.8505
4a
2.0854
3.70
(ppm)
3.1691
Integral
5.2660
1.0000
2.1050
(ppm)

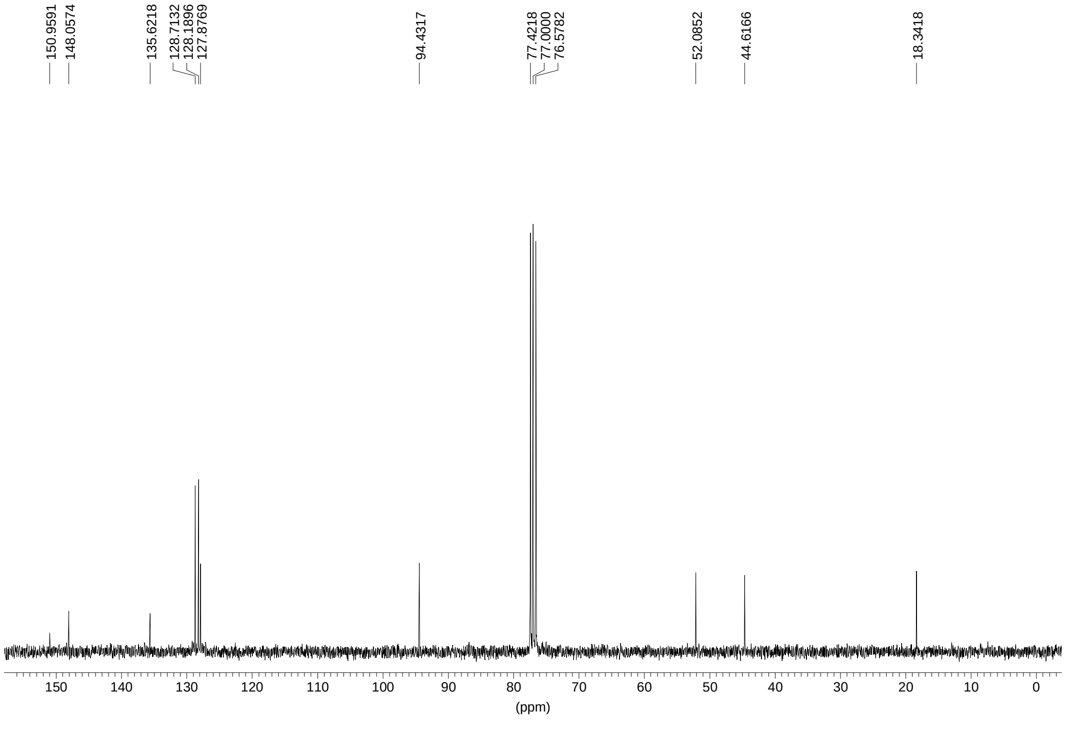
150.9591
148.0574
135.6218
128.7132
128.1896
127.8769
94.4317
77.4218
77.0000
76.5782
52.0852
44.6166
18.3418
(ppm)

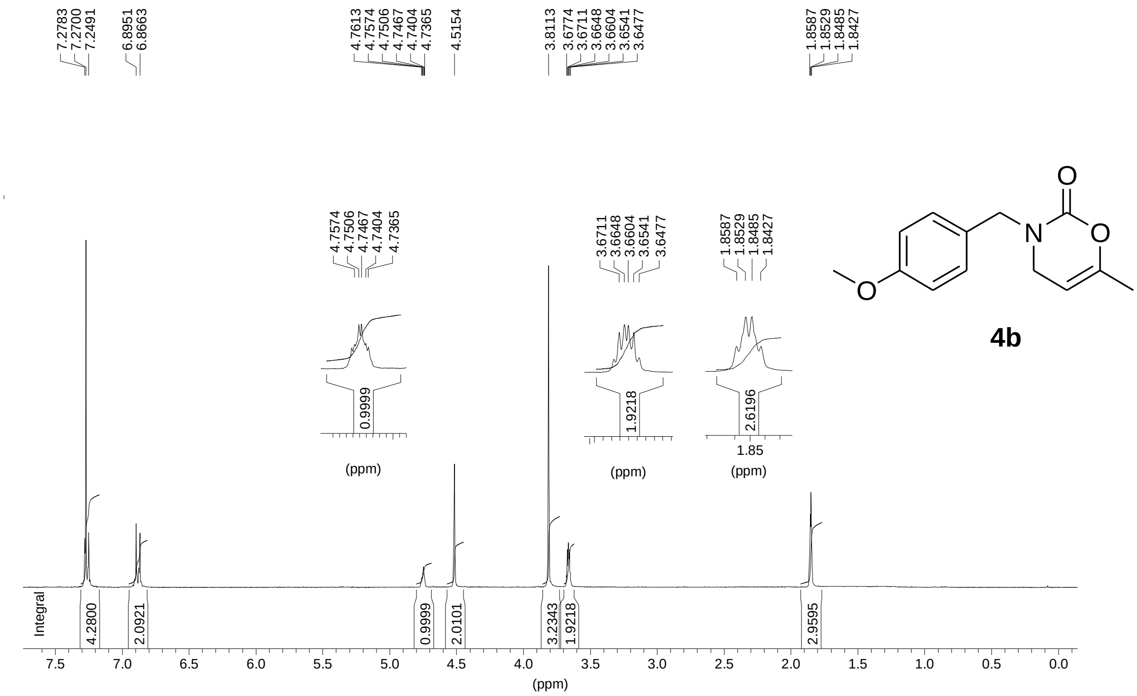
4b
Integral
(ppm)

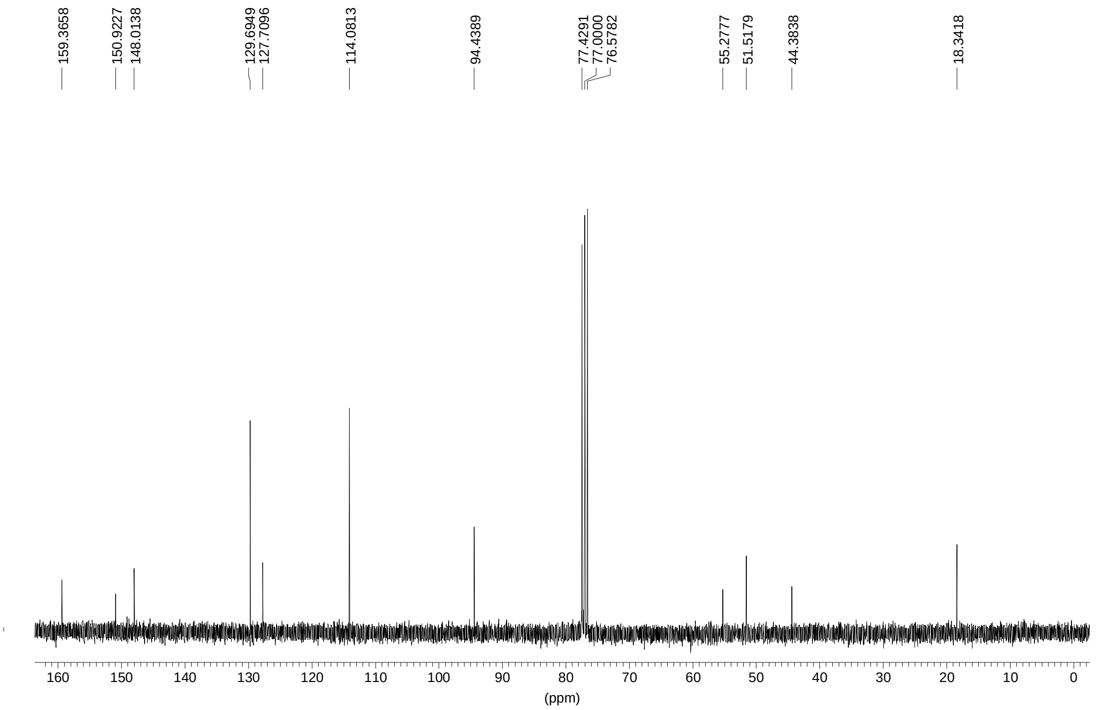
(ppm)

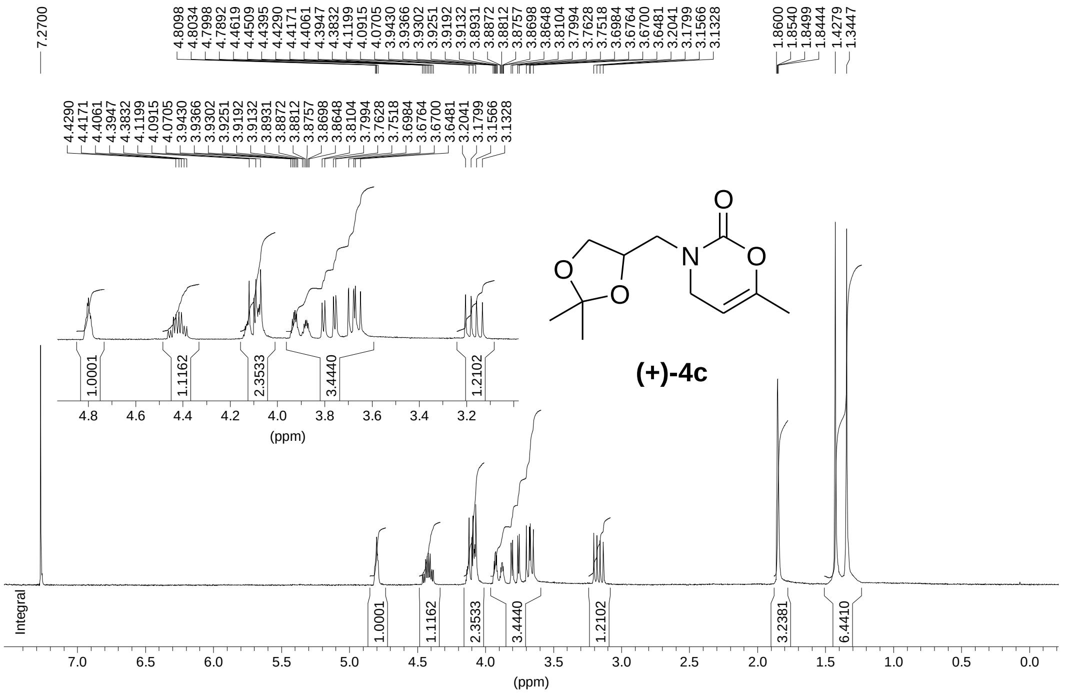
7.2700
4.8098 4.8034 4.7998 4.7892 4.4619 4.4509 4.4395 4.4290 4.4171 4.4061 4.3947 4.3832 4.1199 4.0915 4.0705 3.9430 3.9366 3.9302 3.9251 3.9192 3.9132 3.8931 3.8872 3.8812 3.8757 3.8698 3.8648 3.8104 3.7994 3.7628 3.7518 3.6984 3.6764 3.6700 3.6481 3.2041 3.1799 3.1566 3.1328
1.8600 1.8540 1.8499 1.8444 1.4279 1.3447
4.4290 4.4171 4.4061 4.3947 4.3832 4.1199 4.0915 4.0705 3.9430 3.9366 3.9302 3.9251 3.9192 3.9132 3.8931 3.8872 3.8812 3.8757 3.8698 3.8648 3.8104 3.7994 3.7628 3.7518 3.6984 3.6764 3.6700 3.6481 3.2041 3.1799 3.1566 3.1328
1.0001 1.1162 2.3533 3.4440 1.2102
(ppm)
(+)-4c
Integral
1.0001 1.1162 2.3533 3.4440 1.2102 3.2381 6.4410
(ppm)

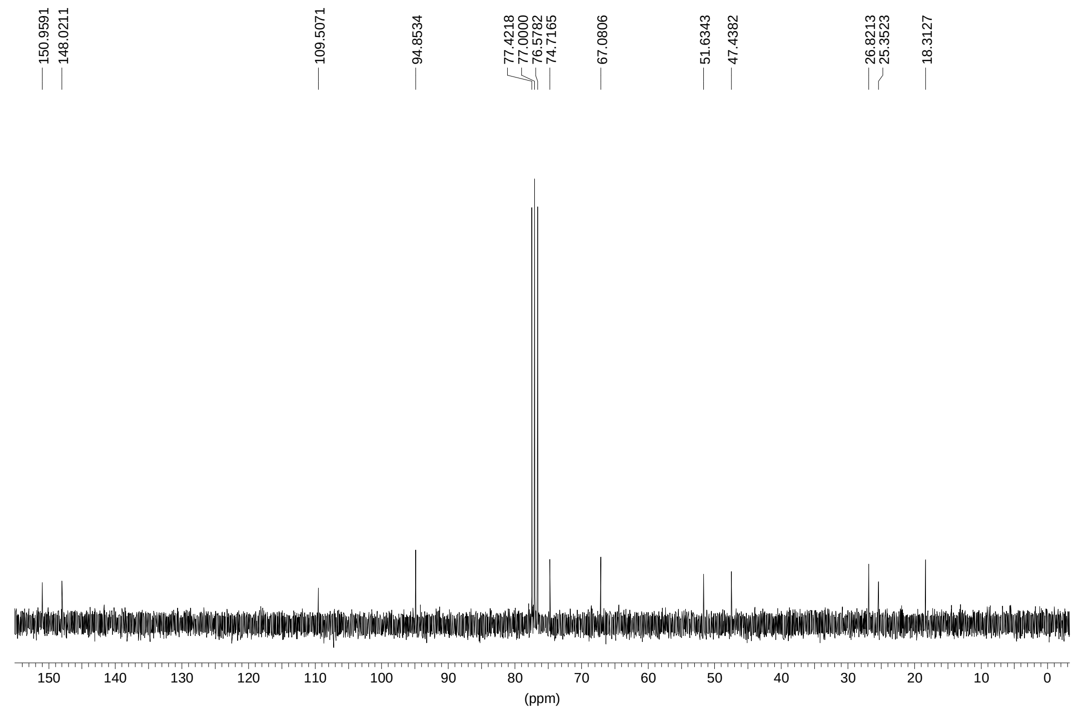
150.9591 148.0211 109.5071 94.8534 77.4218 77.0000 76.5782 74.7165 67.0806 51.6343 47.4382 26.8213 25.3523 18.3127
(ppm)

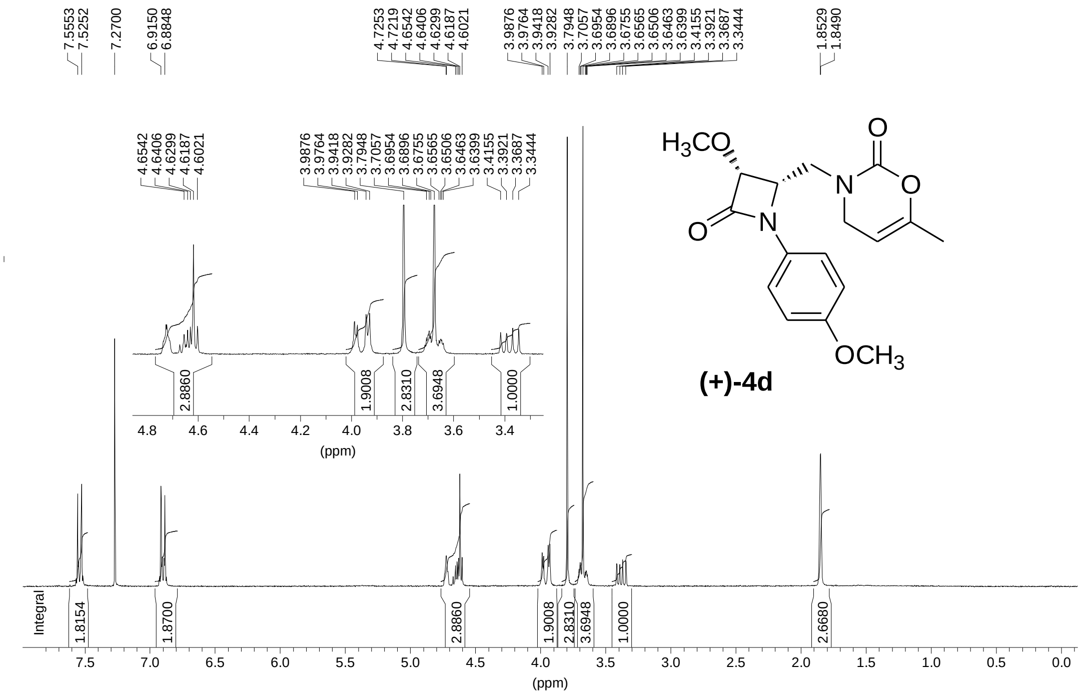
7.5553
7.5252
7.2700
6.9150
6.8848
4.7253
4.7219
4.6542
4.6406
4.6299
4.6187
4.6021
3.9876
3.9764
3.9418
3.9282
3.7948
3.7057
3.6954
3.6896
3.6755
3.6565
3.6506
3.6463
3.6399
3.4155
3.3921
3.3687
3.3444
1.8529
1.8490
H3CO
O
N
O
O
N
OCH3
(+)-4d
2.8860
1.9008
2.8310
3.6948
1.0000
(ppm)
Integral
1.8154
1.8700
2.6680
(ppm)

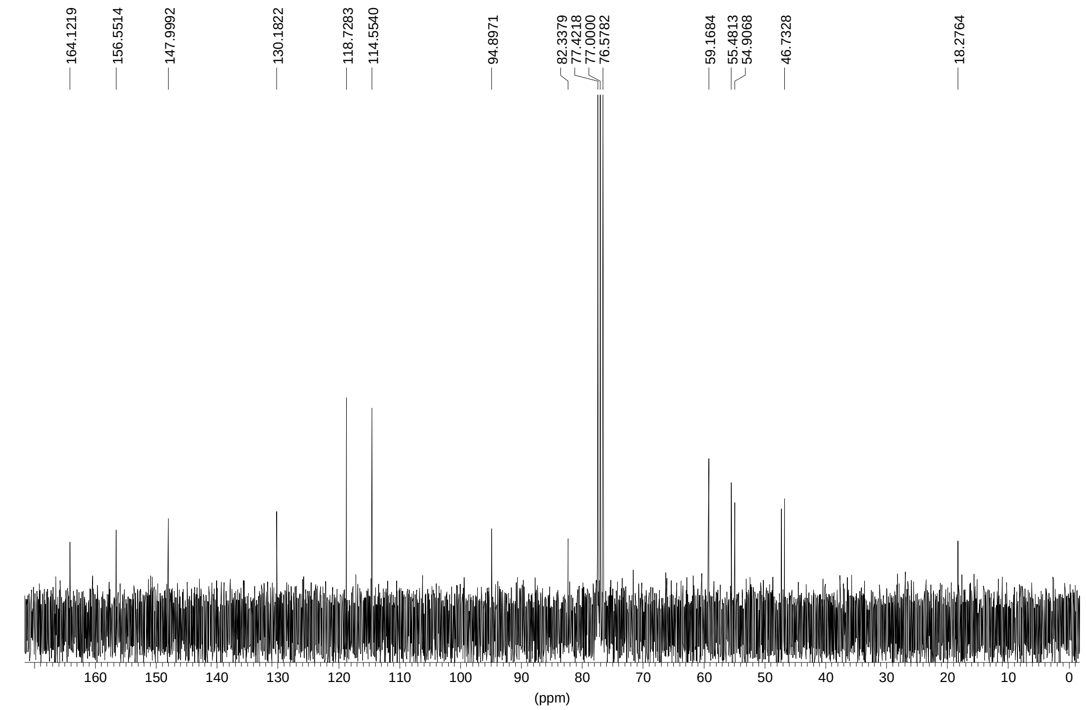
164.1219
156.5514
147.9992
130.1822
118.7283
114.5540
94.8971
82.3379
77.4218
77.0000
76.5782
59.1684
55.4813
54.9068
46.7328
18.2764
(ppm)

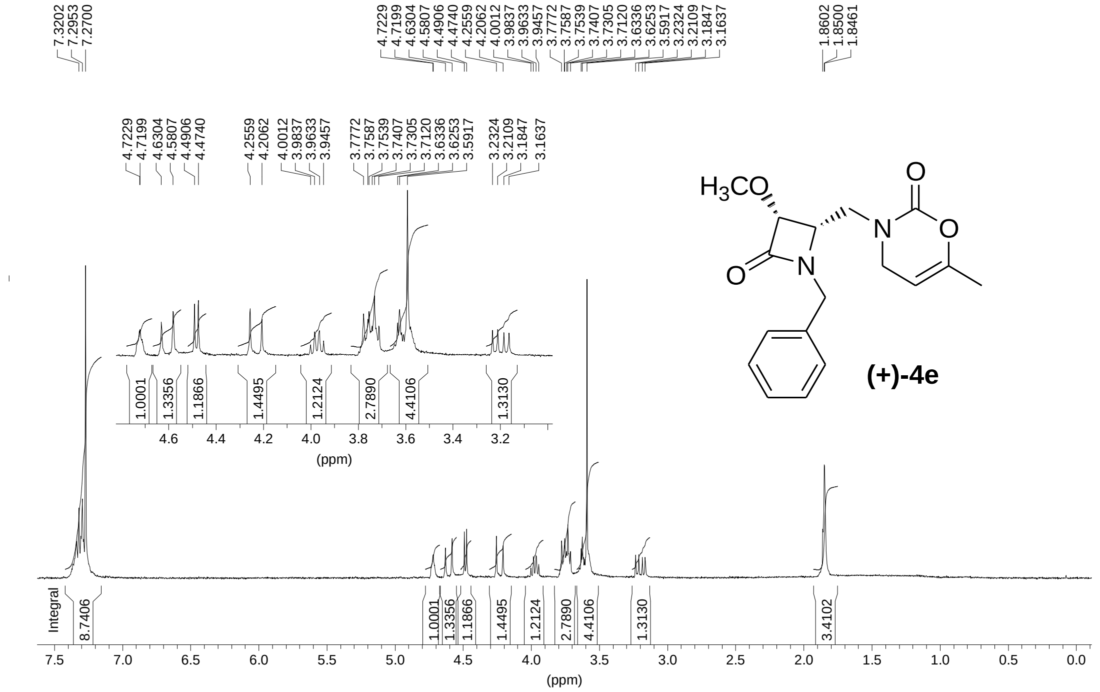
7.3202
7.2953
7.2700
4.7229
4.7199
4.6304
4.5807
4.4906
4.4740
4.2559
4.2062
4.0012
3.9837
3.9633
3.9457
3.7772
3.7587
3.7539
3.7407
3.7305
3.7120
3.6336
3.6253
3.5917
3.2324
3.2109
3.1847
3.1637
1.8602
1.8500
1.8461
H_3CO
N
O
(+)-4e
1.0001
1.3356
1.1866
1.4495
1.2124
2.7890
4.4106
1.3130
(ppm)
Integral
8.7406
3.4102
(ppm)

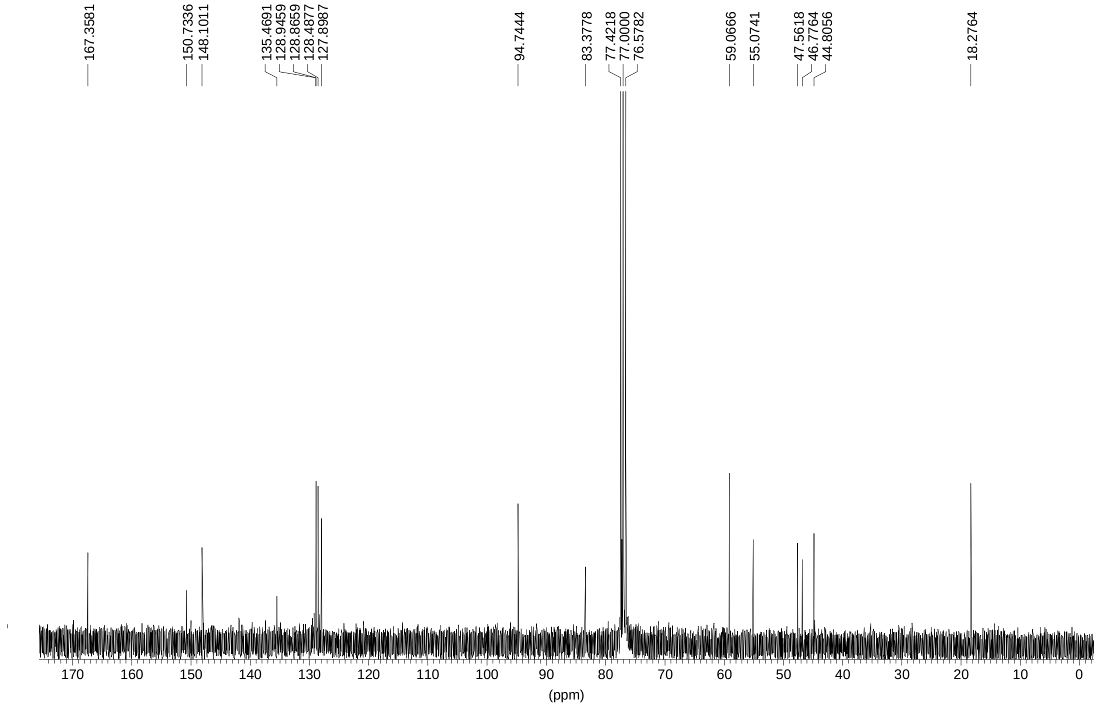
167.3581
150.7336
148.1011
135.4691
128.9459
128.8659
128.4877
127.8987
94.7444
83.3778
77.4218
77.0000
76.5782
59.0666
55.0741
47.5618
46.7764
44.8056
18.2764
(ppm)

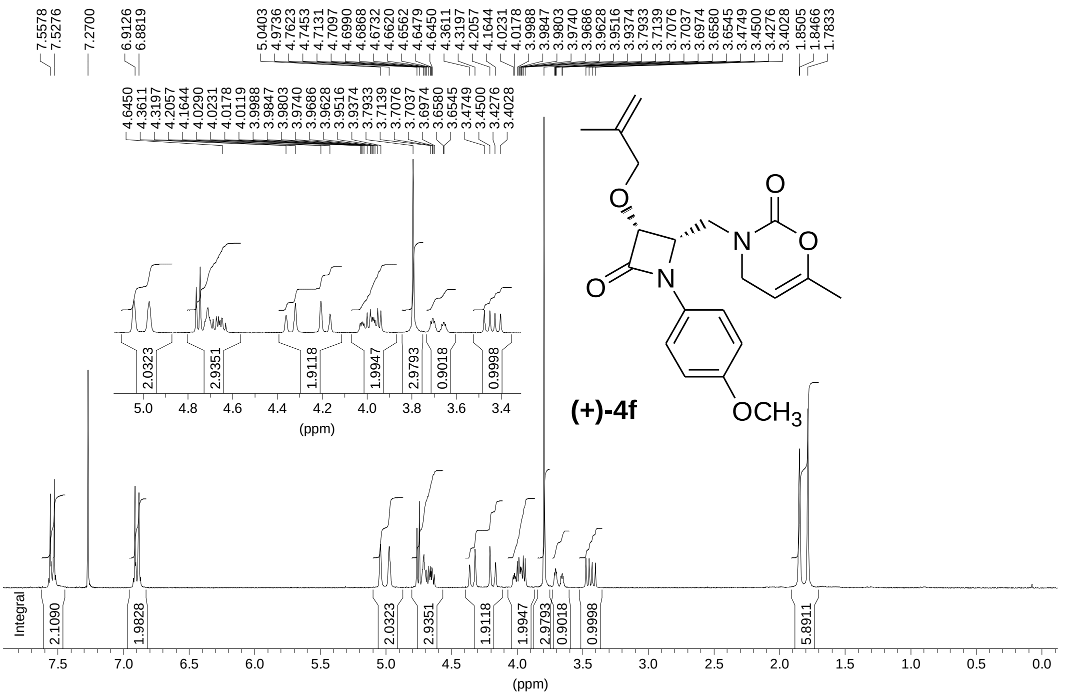

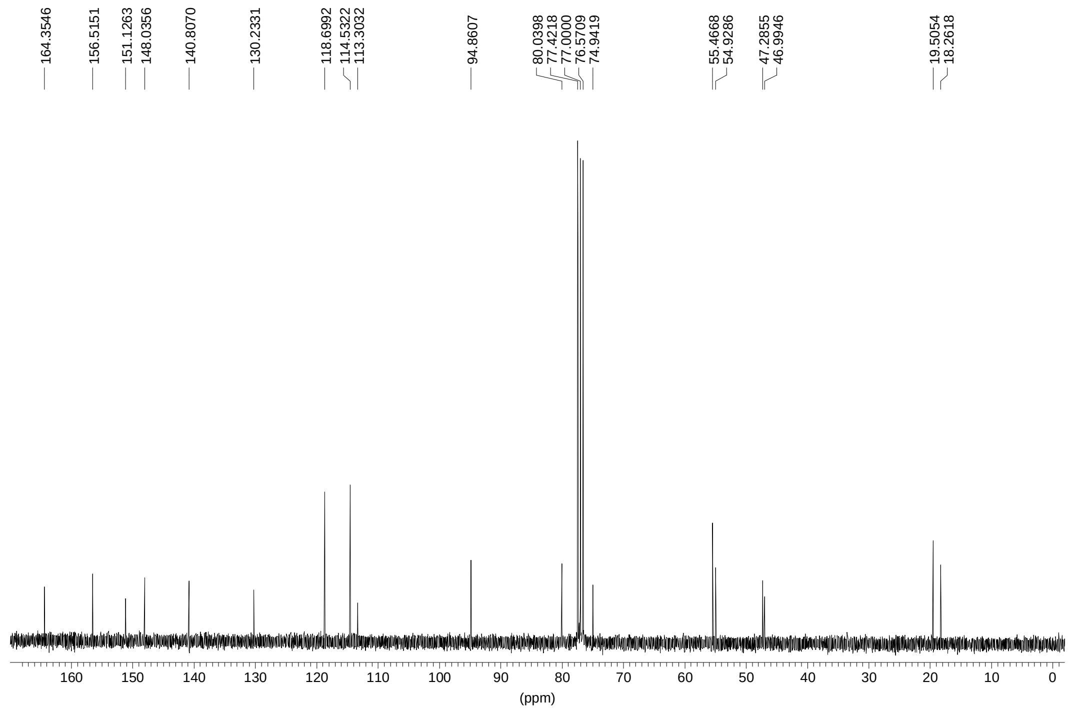

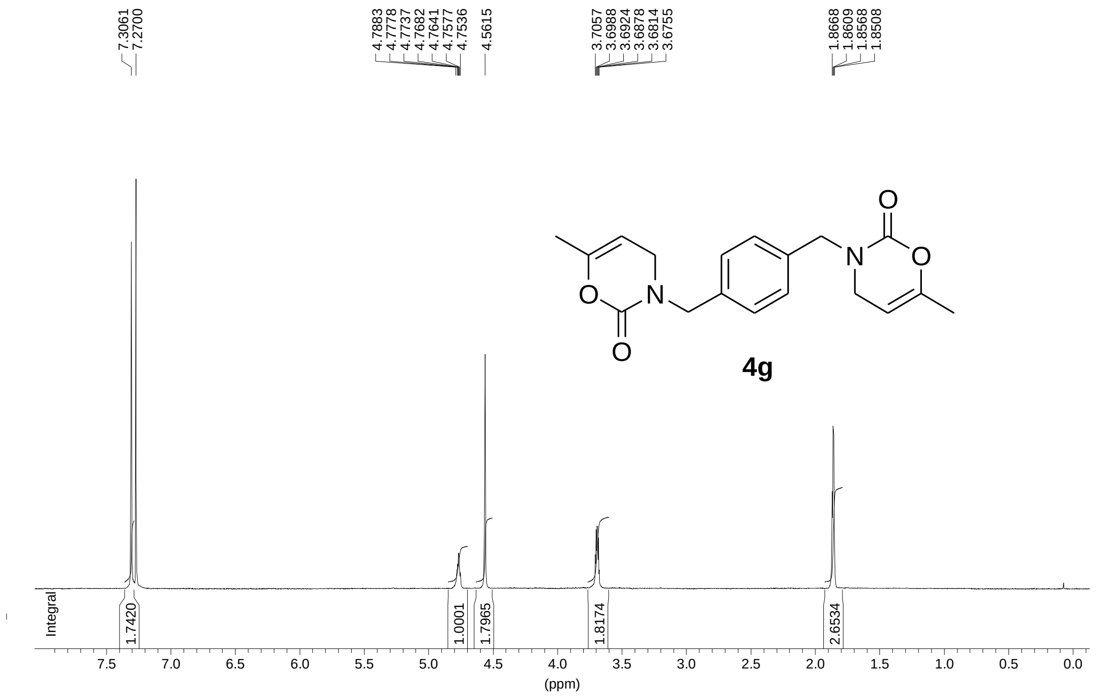
7.3061
7.2700
4.7883
4.7778
4.7737
4.7682
4.7641
4.7577
4.7536
4.5615
3.7057
3.6988
3.6924
3.6878
3.6814
3.6755
1.8668
1.8609
1.8568
1.8508
4g
Integral
1.7420
1.0001
1.7965
1.8174
2.6534
(ppm)

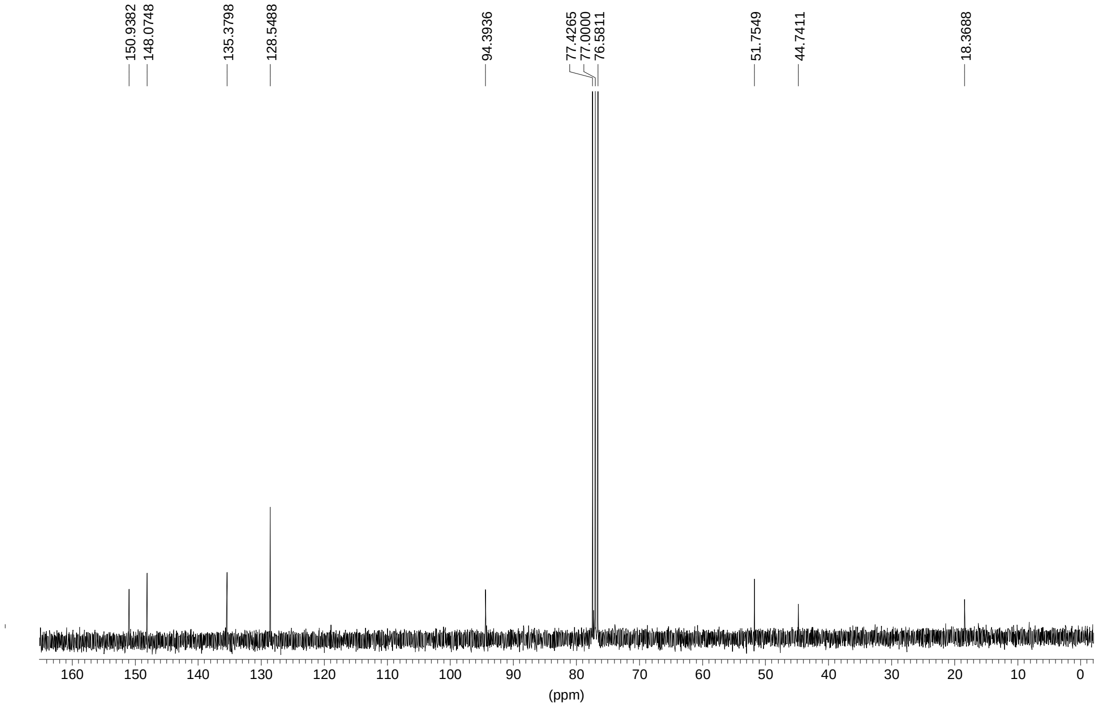
150.9382
148.0748
135.3798
128.5488
94.3936
77.4265
77.0000
76.5811
51.7549
44.7411
18.3688
(ppm)

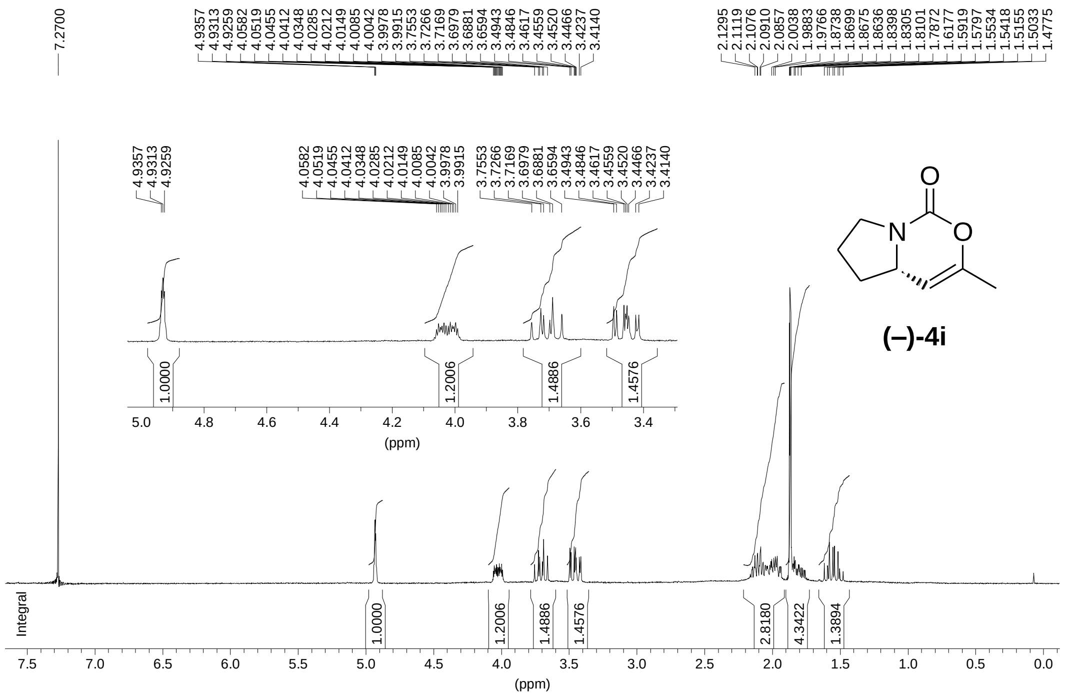
7.2700
4.9357 4.9313 4.9259 4.0582 4.0519 4.0455 4.0412 4.0348 4.0285 4.0212 4.0149 4.0085 4.0042 3.9978 3.9915 3.7553 3.7266 3.7169 3.6979 3.6881 3.6594 3.4943 3.4846 3.4617 3.4559 3.4520 3.4466 3.4237 3.4140
2.1295 2.1119 2.1076 2.0910 2.0857 2.0038 1.9883 1.9766 1.8738 1.8699 1.8675 1.8636 1.8398 1.8305 1.8101 1.7872 1.6177 1.5919 1.5797 1.5534 1.5418 1.5155 1.5033 1.4775
(–)-4i
Integral
1.0000 1.2006 1.4886 1.4576 2.8180 4.3422 1.3894
(ppm)

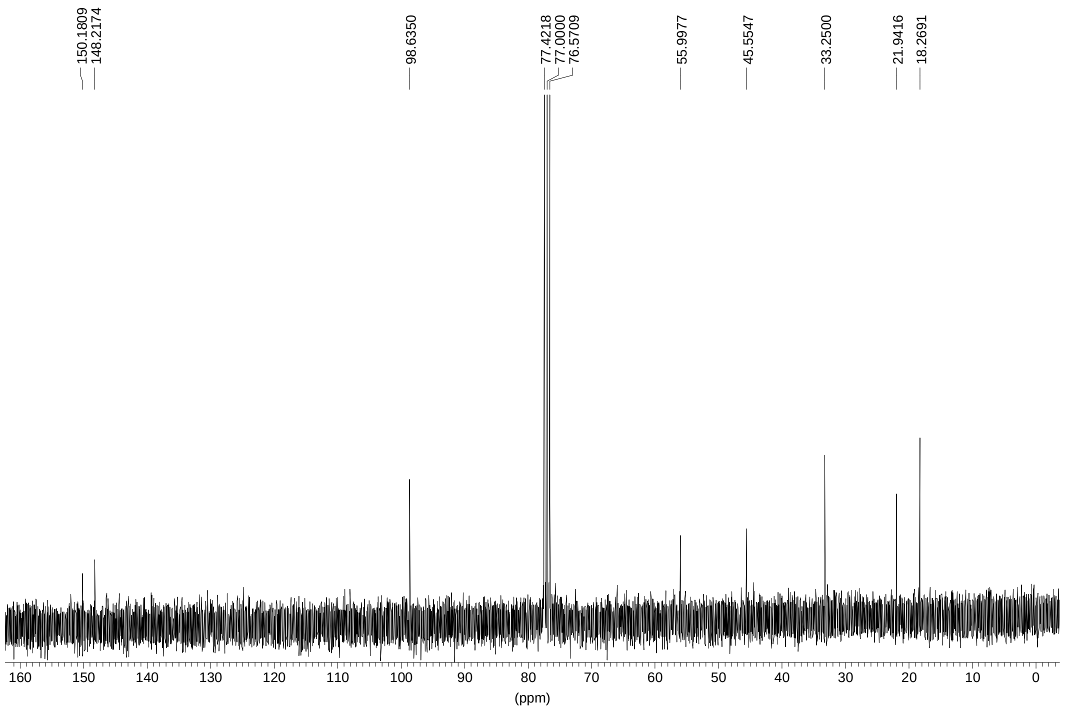
150.1809 148.2174 98.6350 77.4218 77.0000 76.5709 55.9977 45.5547 33.2500 21.9416 18.2691
(ppm)

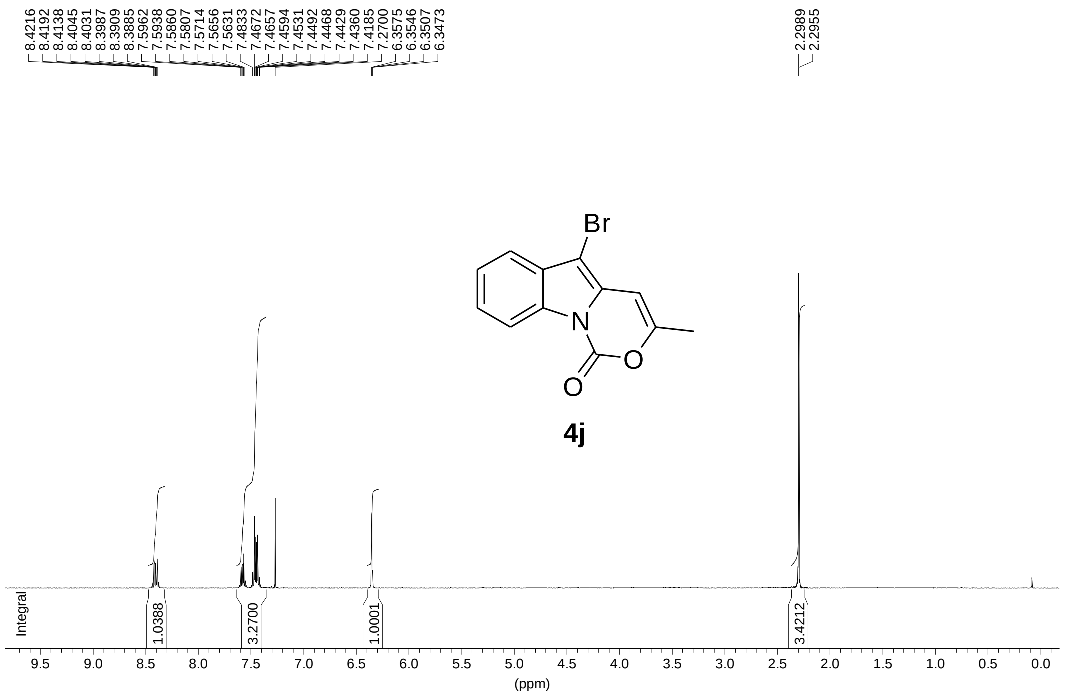
8.4216
8.4192
8.4138
8.4045
8.4031
8.3987
8.3909
8.3885
7.5962
7.5938
7.5860
7.5807
7.5714
7.5656
7.5631
7.4833
7.4672
7.4657
7.4594
7.4531
7.4492
7.4468
7.4429
7.4360
7.4185
7.2700
6.3575
6.3546
6.3507
6.3473
2.2989
2.2955
Br
N
O
O
4j
Integral
1.0388
3.2700
1.0001
3.4212
9.5 9.0 8.5 8.0 7.5 7.0 6.5 6.0 5.5 5.0 4.5 4.0 3.5 3.0 2.5 2.0 1.5 1.0 0.5 0.0
(ppm)

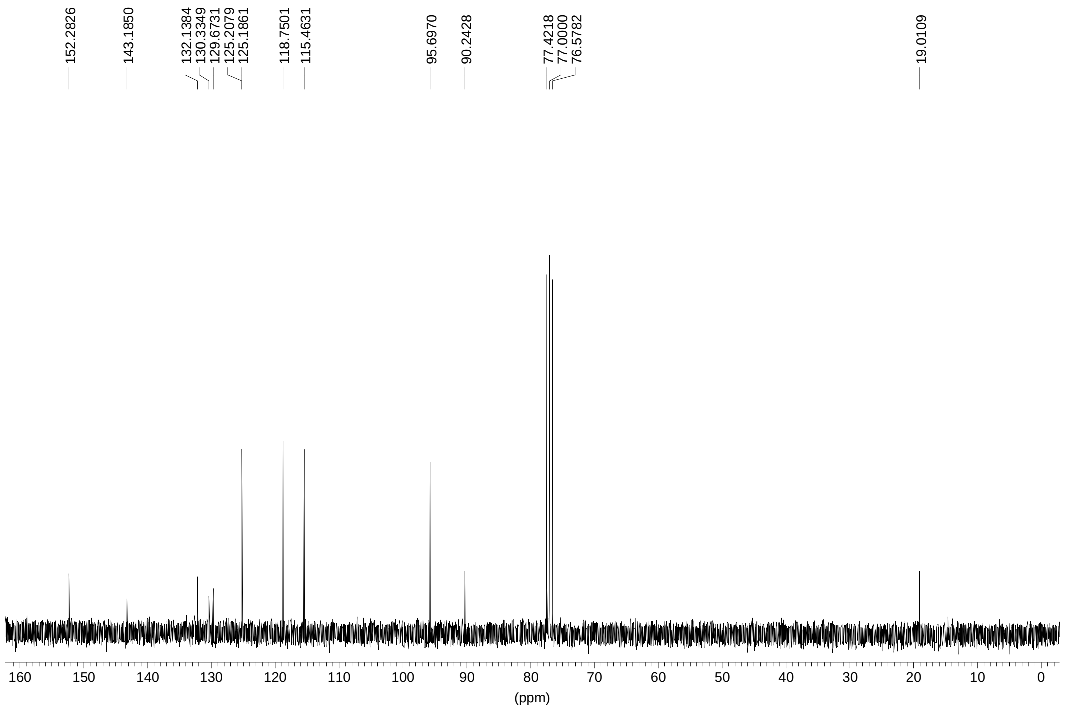
152.2826
143.1850
132.1384
130.3349
129.6731
125.2079
125.1861
118.7501
115.4631
95.6970
90.2428
77.4218
77.0000
76.5782
19.0109
160 150 140 130 120 110 100 90 80 70 60 50 40 30 20 10 0
(ppm)

References

1. *Gaussian 09,* Revision B.01; Gaussian, Inc.: Wallingford CT, 2009.
2. (a) Becke, A. D. *J. Chem. Phys*. **1993**, *98*, 5648. (b) Lee, C.; Yang, W.; Parr, R. G. *Phys. Rev. B* **1998**, *37*, 785. (c) Vosko, S. H.; Wilk, L.; Nusair, M. *Can. J. Phys*. **1980**, *58*, 1200.
3. Weigend, F.; Alhrichs, R. *Phys. Chem. Chem. Phys*. **2005**, *7*, 3297.
4. McIver, J. W.; Komornicki, A. K. *J. Am. Chem. Soc.* **1972**, *94*, 2625.
5. González, C.; Schlegel, H. B. *J. Phys. Chem*. **1990**, *94*, 5523.
6. (a) Miertuš, S.; Scrocco, E.; Tomasi, J. *Chem. Phys*. **1981**, *55*, 117. (b) Pascual-Ahuir, J. L.; Silla, E.; Tuñón, I. *J. Comp. Chem*. **1994**, *15*, 1127. (c) Barone, V.; Cossi, M. *J. Phys. Chem. A*, **1998**, *102*, 1995.
7. Y. Zhao, D. G. Truhlar, *Acc. Chem. Res*. **2008**, *41*, 157.
